# Different Behavior of 2-Substituted 3-Nitro-2*H*-chromenes in the Reaction with Stabilized Azomethine Ylides Generated from α-Iminoesters

**DOI:** 10.3390/molecules27248983

**Published:** 2022-12-16

**Authors:** Ivan A. Kochnev, Alexey Y. Barkov, Nikita S. Simonov, Maria V. Ulitko, Nikolay S. Zimnitskiy, Vladislav Y. Korotaev, Vyacheslav Y. Sosnovskikh

**Affiliations:** Institute of Natural Sciences and Mathematics, Ural Federal University, 51 Lenina Ave., 620000 Ekaterinburg, Russia

**Keywords:** 3-nitro-2*H*-chromenes, azomethine ylides, Michael addition/Mannich reaction sequence, chromeno[3,4-*c*]pyrrolidines, cytotoxicity

## Abstract

The AgOAc-catalysed reaction of 3-nitro-2-phenyl-2*H*-chromenes with stabilized azomethine ylides generated from the imines based on methyl glycinate and arylaldehydes leads to a mixture of *endo* and *endo*’ isomers of the corresponding chromeno[3,4-*c*]pyrrolidines in a ratio of 2.0–2.3:1 in 85–93% total yields as a result of a Michael addition/Mannich reaction sequence. In a similar reaction involving 2-trifluoromethyl-3-nitro-2*H*-chromenes, only *endo* chromeno[3,4-*c*]pyrrolidines are formed in 85–94% yields. 3-Nitro-2-(trichloromethyl)-2*H*-chromenes under the same conditions react with these azomethine ylides to give the corresponding Michael adducts as individual *anti*-isomers with the *cis*,*trans*-configuration of the chromane ring in 40–67% yields. Some 4-CF_3_-substituted chromano[3,4-*c*]pyrrolidines exhibited high cytotoxic activity against HeLa human cervical carcinoma cells.

## 1. Introduction

**Figure 1:**
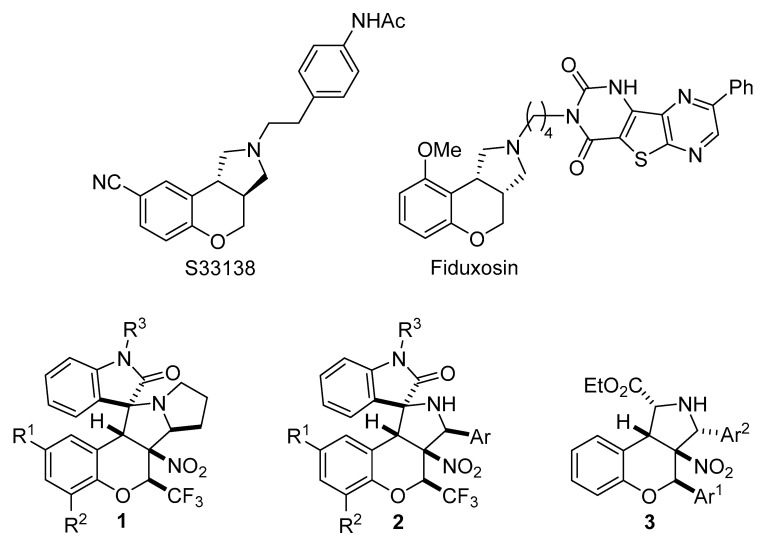
Some bioactive chromenopyrroli(zi)dines.

The chromeno[3,4-*c*]pyrrolidine scaffold is the main structural element of a number of bioactive molecules with important pharmaceutical properties. For example, the *trans*-chromeno[3,4-*c*]pyrrolidine derivative S33138 is a dopamine D_3_ receptor antagonist and a potential drug for the treatment of CNS disorders such as schizophrenia and Parkinson’s disease [1], while its *cis*-derivative, fiduxosin, is an α_1_-adrenoceptor antagonist and a promising drug for the treatment of benign prostatic hyperplasia [2] (Figure 1). It was recently reported that spirooxindole derivatives of chromenopyrroli(zi)dines **1** and **2** show high antitumor activity against human cervical carcinoma and human rhabdomyosarcoma cancer cells along with low cytotoxicity against normal human dermal fibroblast [3,4,5]. Fused prolinates **3** have been successfully tested as antimycobacterial agents against the *M. tuberculosis* H37Rv strain [6]. Therefore, the development of regio- and stereoselective methods for the synthesis of novel Δ^3^-fused chromenopyrrolidine derivatives is an urgent task.

A convenient one-pot atom-economical method for the synthesis of functionalized pyrrolidines is based on the reaction of electron-deficient alkenes with stabilized azomethine ylides generated in situ from Schiff bases [7,8,9,10,11,12]. Due to the high regio- and stereoselectivity of reactions involving these ylides, this approach is an indispensable tool in the synthesis of complex heterocyclic molecules containing up to four new chiral centers with the required arrangement and spatial orientation of substituents from relatively simple and commercially available precursors. When amino acid esters are used as the amino component of the Schiff base, prolinates are formed as reaction products. These reactions are usually carried out in the presence of catalytic amounts of a Brønsted base and transition metal or lithium salt as a Lewis acid. Lewis acids increase the stereoselectivity of the process by stabilizing the W-conformation of the ylide [13,14].

**Scheme 1:**
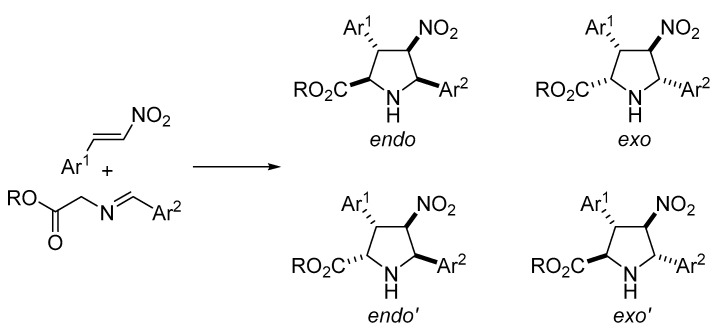
Possible diastereomers generated from α-iminoesters and *trans*-nitrostyrenes.

Being readily available and highly reactive substrates, conjugate nitroalkenes are widely used as dipolarophiles for the stereoselective synthesis of nitroprolinates [15,16,17,18]. In the reactions of *trans*-nitrostyrene with iminoesters, four diastereomers can be formed, classified as *endo*, *exo*, *endo’* and *exo’* isomers (Scheme 1).

Depending on the type of catalyst, *endo* [19,20,21,22], *exo* [23,24] or *exo’* [25] isomers can be obtained as a result of a diastereo- and enantioselective concerted 1,3-dipolar cycloaddition or of a Michael addition/Mannich reaction sequence. Heating without a Lewis acid and a base gave the mixtures of *endo*, *exo*, *endo*’ [26] or *endo* and *exo* [27] isomers.

**Scheme 2:**
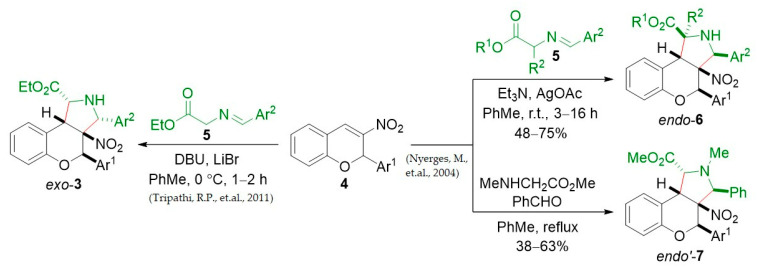
Reactions of 2-aryl-substituted 3-nitro-2*H*-chromenes **4** with azomethine ylides generated from amino acid esters and arylaldehydes [6,31].

Due to the presence of a β-nitrostyrene moiety, 3-nitro-2*H*-chromenes can also react with azomethine ylides to form chromenopyrroli(zi)dine derivatives [28,29,30]. There are only four reports on the reactions of nitrochromenes with stabilized azomethine ylides based on amino acid esters and arylaldehydes [6,31,32,33] (Scheme 2). It was reported that the reaction between 2-aryl-substituted nitrochromenes **4** and Schiff bases **5** in the presence of DBU and LiBr resulted in adducts *exo*-**3** [6], while the same reaction in the presence of Et_3_N and AgOAc led to products **6** as individual *endo* isomers [31]. If the ylide from methyl sarcosinate and benzaldehyde was used as a reagent, only adducts endo*’*-**7** were obtained [31]. In the works [32,33], the synthesis of products *endo*-**6** (Ar^2^ = Ph, R^1^ = Et, R^2^ = CO_2_Et) from chromenes **4** and the corresponding iminoester **5** in the presence of a chiral base [32] or by a three-component reaction involving 3-nitro-2-phenyl-2*H*-chromene, diethyl 2-aminomalonate and benzaldehyde without a catalyst [33] has been described.

Our group’s science research is focused on the development of methods for Δ^3^-carbo- and heteroannulation of 2-trifluoromethyl-substituted 3-nitro-2*H*-chromenes using available ambiphilic reagents [3,4,5,34,35]. The introduction of the electron-withdrawing CF_3_-group in position 2 of 3-nitro-2*H*-chromene not only activates the double bond but also increases the stereoselectivity of their reactions with nucleophiles and ambiphiles. Furthermore, the replacement of the methyl group by the trifluoromethyl one in the bioactive molecule can lead to an increase in pharmacological properties due to enhanced lipophilicity and metabolic stability [36,37,38].

In this work, the behavior of 2-phenyl- and 2-trifluoro(trichloro)methyl-substituted 3-nitro-2*H*-chromenes **4** in the reaction with stabilized azomethine ylides generated from α-iminoesters **5** in the presence of Et_3_N and AgOAc have been compared and cytotoxic activity of some 4-phenyl- and 4-trifluoromethyl-substituted chromeno[3,4-*c*]pyrrolidine derivatives has been studied.

## 2. Results and Discussion

**Scheme 3:**
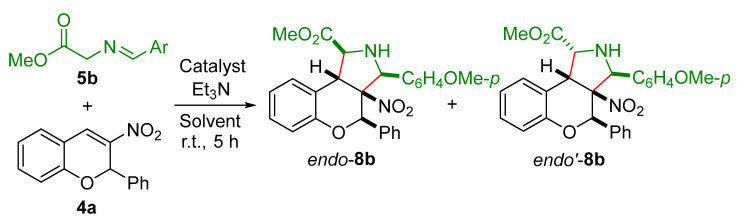
The reaction of nitrochromene **4a** with azomethine **5b**.

To obtain 4-phenyl-substituted chromeno[3,4-*c*]pyrrolidines **8**, we used the Nyerges group’s method [31], but the amount of AgOAc was reduced from 150 to 10 mol%. The reaction between chromene **4a** and imine ester **5b** (Ar = 4-MeOC_6_H_4_) in the presence of Et_3_N and AgOAc in toluene at room temperature for 5 h led to the mixture of *endo*-**8b** and *endo’*-**8b** isomers in a ratio of 2.1:1 in 60% total yield (Scheme 3, Table 1, entry 1). It motivated us to optimize the conditions for this reaction. Replacing toluene with acetonitrile or tetrahydrofuran led to an increase in the yield of the target product to 91–92% (Table 1, entries 2–3). The best yield of adduct **8b** (93%) was achieved when the reaction was carried out in dichloromethane (DCM) (Table 1, entry 4). The use of CuI as a catalyst proved to be less efficient (Table 1, entries 5–8). Regardless of the nature of the catalyst and solvent, the ratio of stereoisomers remained unchanged.

**Scheme 4:**
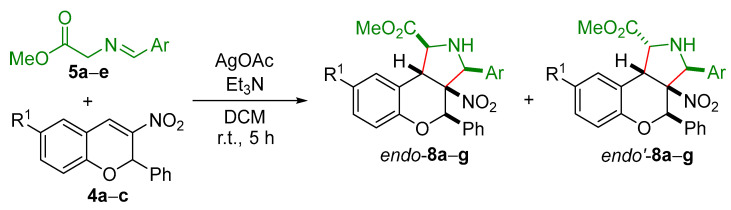
Synthesis of 4-Ph-substituted chromeno[3,4-*c*]pyrrolidines **8**.

Under optimized conditions, chromeno[3,4-*c*]pyrrolidines **8a−g** were obtained as mixtures of *endo* and *endo’* isomers in a 2.0–2.3:1 ratio with 85–93% total yields (Scheme 4, Table 2). The donor-acceptor properties of substituents in chromene **4** and in the aryl fragment of the α-iminoester **5** had no significant effect on the yields of products **8** and the diastereoselectivity of the reaction. Individual isomers *endo*-**8a−g** and *endo’*-**8a−g** in 54–61% and 20–27% yields, respectively, were prepared after the purification of crude products by column chromatography.

**Scheme 5:**
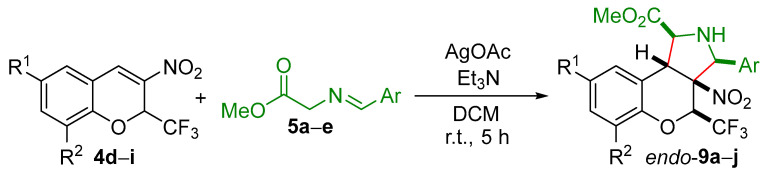
Synthesis of 4-CF_3_-substituted chromeno[3,4-*c*]pyrrolidines **9**.

Next, we examined 2-trifluoromethyl-substituted chromenes **4d−i** in the reaction with iminoesters **5a−e**. It was found that under the same conditions, adducts *endo*-**9a−j** are formed in 85–94% yields as the only reaction products (Scheme 5, Table 3). Other isomers were not detected in the reaction mixtures by ^19^F NMR spectroscopy. The product yields also did not depend on the nature of the substituents in the starting chromenes **4** and Schiff bases **5**.

**Scheme 6:**
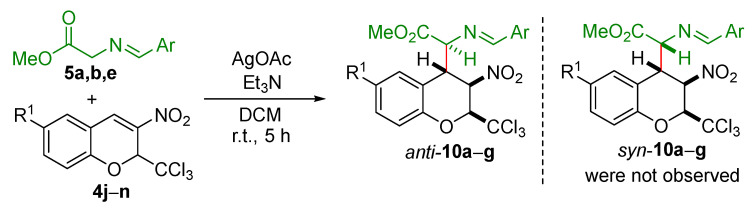
Synthesis of 2-CCl_3_-substituted chromanes **10**.

3-Nitro-2-(trichloromethyl)-2*H*-chromenes **4j−n** under the same conditions react with iminoesters **5a**,**b**,**e** to give the corresponding Michael adducts **10a−g** in 40–67% yields as individual *anti* isomers with the *cis*,*trans* configuration of the chromane ring (Scheme 6, Table 4). The lowest yield (40%) was observed in the reaction involving nitrochromene **4n** with the MeO group in position 6. The isomers *syn*-**10** were not observed in the reaction mixtures.

**Scheme 7:**
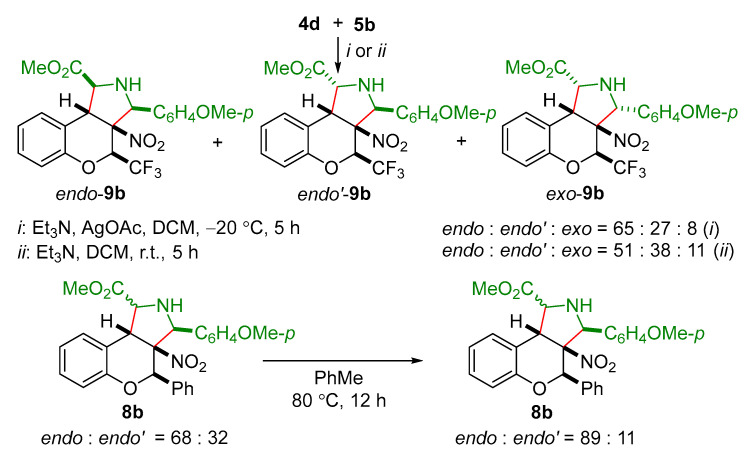
Control experiments.

To understand the reason for such different stereoselectivity of the reactions involving 2-Ph- and 2-CF_3_-substituted chromenes **4**, additional experiments have been performed. If the reaction between chromene **4d** and iminoesters **5b** was carried out at −20 °C, the mixture of the products *endo*-**9b**, *endo’*-**9b** and *exo*-**9b** was obtained in a ratio of 65:27:8, respectively (Scheme 7). When this process was carried out at room temperature without AgOAc, the content of the isomer *endo’*-**9b** increased to 38% (*endo*-**9b**:*endo’*-**9b**:*exo*-**9b** = 51:38:11), and the total yield decreased to 42%. If the crude mixture of compounds *endo*-**8b** *endo’*-**8b** was heated in toluene for 12 h, the content of the *endo’* isomer was reduced to 11%.

**Scheme 8:**
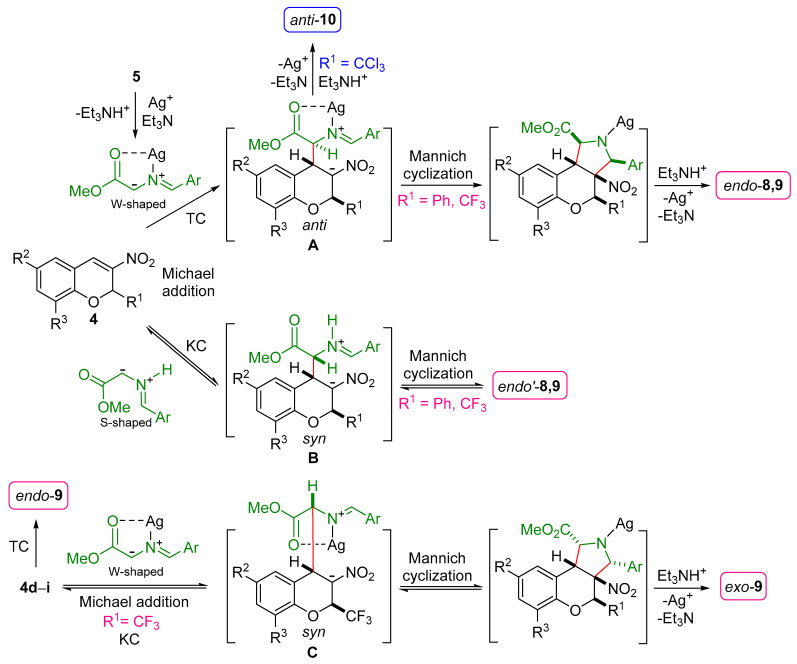
Proposed mechanism for the AgOAc-catalyzed reaction of chromenes **4** with iminoesters **5**.

Thus, chromeno[3,4-*c*]pyrrolidines *endo*-**8**,**9** and *exo*-**9** are formed as a result of the Michael addition of W-shaped ylides to chromenes **4** followed by Mannich cyclization through intermediates **A** and **C**. (Scheme 8). A similar process involving S-shaped ylides leads to minor products *endo’*-**8**,**9** through intermediate **B**. Apparently, S-ylides are formed in the presence of a slight excess of Et_3_N relative to AgOAc in the reaction mixture. If chromene **4** contains a trichloromethyl group at position 2, closing the pyrrolidine ring is impossible due to steric repulsions between the CCl_3_ and Ar substituents. In this case, the end products of the reaction are Michael adducts *anti*-**10**.

The addition of azomethine ylides to chromenes **4** occurs reversibly. The isomers *endo’*-**8**,**9** and *exo*-**9** are kinetic control products (KC) and convert into the thermodynamically more stable isomers *endo*-**8**,**9** at higher temperatures (TC). In the case of more reactive 2-CF_3_-chromenes **4**, the reverse reaction proceeds even at room temperature under the reaction conditions.

**Figure 2:**
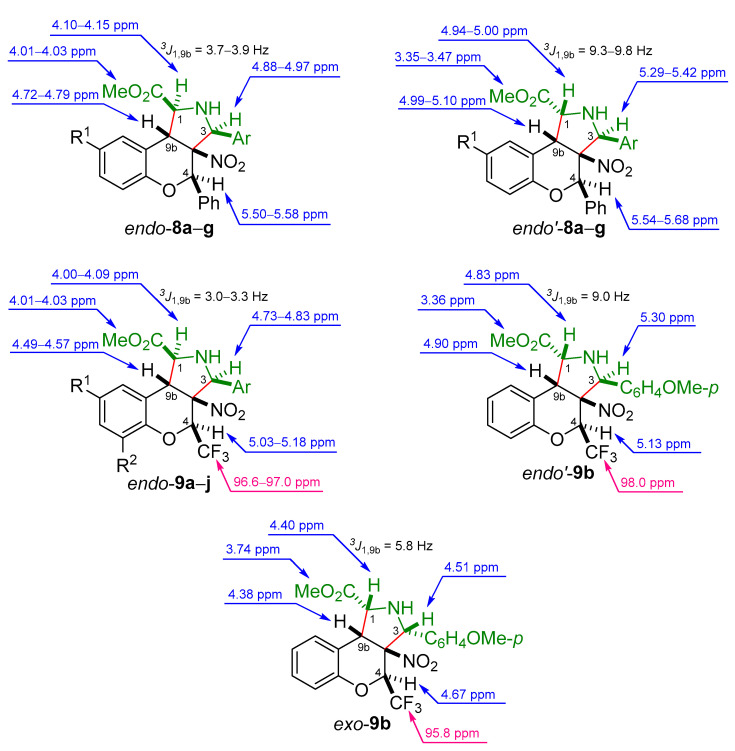
Characteristic chemical shifts of stereoisomeric chromeno[3,4-*c*]pyrrolidines **8** and **9** in ^1^H (in blue) and ^19^F (in magenta) NMR spectra in CDCl_3_.

The structure and relative configuration of compounds **8**–**10** were confirmed by 1D and 2D NMR spectroscopy and X-ray single-crystal analysis. In the ^1^H NMR spectra of chromeno[3,4-*c*]pyrrolidines **8** and **9**, signals of the H-1, H-3, H-4 and H-9b characteristic protons are observed (Figure 2). In the spectra of isomers, *endo-***8a−g** and *endo*-**9a−j** acquired in CDCl_3_, the signal of the H-1 proton manifested as a doublet or a doublet of doublets at 4.00–4.15 ppm with the spin-spin coupling constant ^3^*J*_1,9b_ = 3.0–3.9 Hz. A doublet of the H-9b proton in these isomers manifested in the range of 4.49–4.79 ppm. In the spectra of isomers *endo’-***8a−g** and *endo’-***9b**, both of these protons are observed as doublets in the range of 4.83–5.00 and 4.90–5.10 ppm, respectively, with the coupling constant ^3^*J*, = 9.3–9.8 Hz. In the spectra of *endo* isomers, the signal of the H-3 proton manifested at 4.73–4.97 ppm, while in the spectra of *endo’* isomers, this proton is deshielded and is observed at 5.29–5.42 ppm. The signal of the H-4 proton manifested as a singlet in the range of 5.50–5.68 ppm in the spectra of adducts *endo*-**8a−g** and *endo’*-**8a−g** or as a quartet at 5.03–5.18 ppm with the coupling constant ^3^*J*_H,F_ = 6.8–7.0 Hz in the spectra of adducts *endo*-**9a−j** and *endo’*-**9b**. In the ^1^H NMR spectrum of isomer *exo-***9b**, signals of the H-1, H-3, H-4 and H-9b characteristic protons are observed at 4.40, 4.51, 4.38 and 4.67 ppm, respectively. The coupling constant ^3^*J*_1,9b_ is 5.8 Hz. The ^19^F NMR spectra of isomers *endo*-**9a−j**, *endo’*-**9b** and *exo-***9b** contain doublets of CF_3_-group at 96.6–97.0, 98.0 and 95.8 ppm with coupling constants 6.8–7.0, 7.0 and 7.2 Hz, respectively.

**Figure 3:**
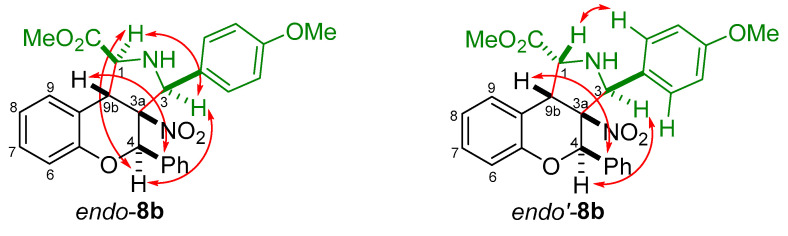
The main correlations in the 2D ^1^H−^1^H NOESY spectra of *endo*-**8b** and *endo’*-**8b**.

In the 2D ^1^H−^1^H NOESY spectrum of compound *endo*-**8b**, the cross-peaks H-1↔H-3, H-1↔H-4, H-3↔H-4 and H-9b↔H*_o_* Ph are observed, which indicate the *cis* arrangement of the H-1, H-3 and H-4 hydrogen atoms relative to the fused tricyclic system (Figure 3). The 2D ^1^H−^1^H NOESY spectrum of compound *endo’*-**8b** has shown the cross-peak H-1↔H-2,6 4-MeOC_6_H_4_ along with the cross-peaks H-3↔H-4 and H-9b↔H*_o_* Ph, which indicate the *trans* arrangement of the H-1 and H-3 atoms and the *cis* arrangement of the H-3 and H-4 atoms. The cross-peak H-9b↔H-3 is not observed in the spectra of both isomers.

**Figure 4:**
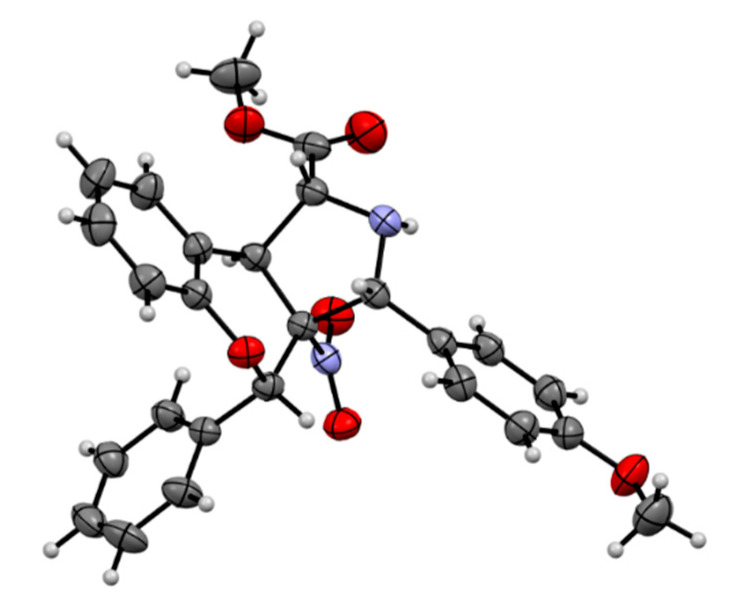
Molecular structure of *endo*-**8b** (ORTEP drawing, 50% probability level).

**Figure 5:**
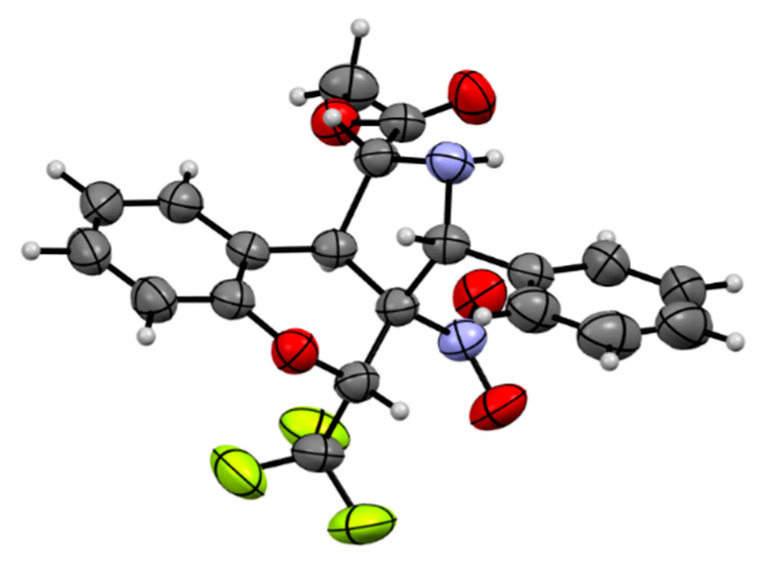
Molecular structure of *endo*-**9a** (ORTEP drawing, 50% probability level).

The relative configuration of *endo* chromeno[3,4-*c*]pyrrolidines **8** and **9** was unambiguously confirmed by single crystal X-ray diffraction analysis of compounds *endo*-**8b** and *endo*-**9a** (Figure 4 and Figure 5). In both molecules, the H-1, H-3, and H-4 atoms are located on one side of the condensed tricyclic system, with the 4-Ph or 4-CF_3_-group occupying the axial position, while the nitro group is in the equatorial positions. The pyran and pyrrolidine rings have half-chair and twist conformations, respectively.

In the ^1^H NMR spectra of 2-CCl_3_-substituted chromanes **10a−g**, the signals of the H-2*’*, H-3*’* and H-4*’* protons of the chromane ring in the range of 5.18–5.30, 6.25–6.29 and 4.08–4.14 ppm, respectively, with spin-spin coupling constants ^3^*J*_2*’*,3*’*_ ≈ ^3^*J*_3*’*,4*’*_ ≈ 1.0–1.8 Hz, and a singlet of the vinylic proton at 7.87–7.99 ppm are observed (see Supplementary Materials for NMR spectra).

**Figure 6:**
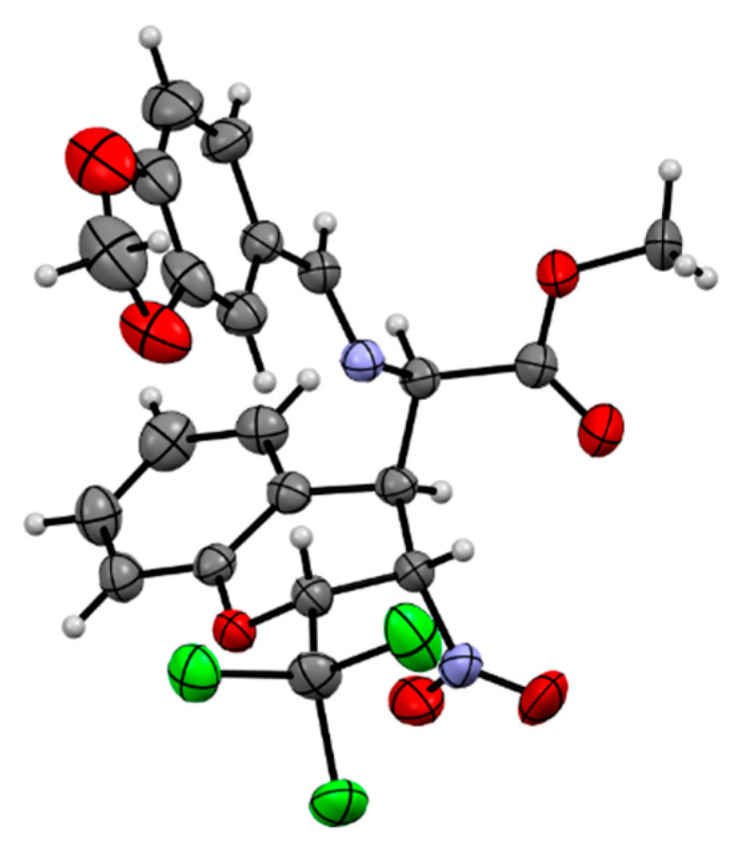
Molecular structure of **10c** (ORTEP drawing, 30% probability level).

The structure and relative configuration of chromane **10c** was unambiguously confirmed by single crystal X-ray diffraction analysis (Figure 6). In this molecule, the nitro and CF_3_ groups are located on the same side of the pyran ring, with the latter occupying the equatorial position. The iminoester fragment and the nitro group are arranged *trans*-diaxially. The pyran ring has a distorted half-chair conformation.

For representative chromeno[3,4-*c*]pyrrolidines *endo*-**8b**,**e−g** and *endo*-**9e**,**f−j**, their in vitro cytotoxic activity against HeLa cervical cancer and human dermal fibroblast cells (HDF) was evaluated. The known cytotoxin camptothecin [39] was used for comparison (Table 5). Of all the tested 2-Ph-substituted chromeno[3,4-*c*]pyrrolidines **8**, only *endo-***8e** bearing a benzo[*d*][1,3]dioxol-5-yl substituent at position 3 showed noticeable cytotoxic activity against HeLa cells. Compound *endo*-**9j** with a *p*-methoxyphenyl substituent at position 3 and the EtO group at position 6 is cytotoxic to HeLa and HDF cells. Compound *endo*-**9b** with a *p*-methoxyphenyl group at position 3 exhibited a high antitumor activity along with low toxicity and is a promising drug candidate.

In summary, it has been found that the addition of azomethine ylides derived from α-iminoesters to 2-Ph- and 2-CF_3_-substituted 3-nitro-2*H*-chromenes proceeds as a reversible Michael addition/Mannich reaction sequence. The reaction of these ylides with 2-CCl_3_-chromenes stops at the Michael addition step. The stereochemistry of chromenoprolinates can be controlled by varying the temperature and solvent. One-pot stereoselective approaches to the synthesis of 4-(trifluoromethyl)-substituted chromeno[3,4-*c*]pyrrolidines and methyl 2-(arylideneamino)-2-(2-(trichloromethyl)chroman-4-yl)acetates from available reagents have been developed. Some 4-CF_3_-substituted chromeno[3,4-*c*]pyrrolidine derivatives have shown high antitumor activity and are of undoubted interest in medicinal chemistry.

## 3. Materials and Methods

### 3.1. General

IR spectra were recorded on a Shimadzu IRSpirit-T spectrometer (Shimadzu Corp., Kyoto, Japan) using an attenuated total reflectance (ATR) unit (FTIR mode, diamond prism), and the absorbance maxima (*ν*) are reported in cm^–1^. NMR spectra were recorded on Bruker Avance III-500 (work frequencies: ^1^H—500 MHz, ^19^F—471 MHz, ^13^C—126 MHz) and Bruker DRX-400 (Bruker BioSpin GmbH, Ettlingen, Germany, work frequencies: ^1^H—400 MHz; ^19^F—376 MHz) spectrometers in CDCl_3_. The chemical shifts (*δ*) are reported in ppm relative to the internal standard TMS (^1^H NMR), C_6_F_6_ (^19^F NMR), and residual signal of the solvent (^13^C NMR). 2D NMR spectra were acquired on Bruker AVANCE NEO (600 MHz) and Bruker AVANCE 400 spectrometers. The HRMS spectra were obtained using the UHR-QqTOF maXis Impact HD mass spectrometer. Melting points were determined on an SMP40 apparatus. Column chromatography was performed on silica gel (Merck 60, 70–230 mesh, Darmstadt, Germany). All solvents used were dried and distilled by standard procedures. The starting chromenes **4a−c** and **4d−n** were prepared according to described procedures [40,41]. Schiff bases **5** were obtained according to the described procedure [42].

### 3.2. Synthesis of Compounds ***8a–g***

General procedure. A mixture of the appropriate 3-nitro-2-phenyl-2*H*-chromene **4** (0.5 mmol), azomethine **5** (0.55 mmol), Et_3_N (7 μL, 5 mg, 0.05 mmol) and AgOAc (5.8 mg, 0.05 mmol) was stirred in dichloromethane (2 mL) for 5 h at room temperature (TLC control, EtOAc−hexane (1:2)). Upon completion of the reaction, the residue was evaporated under reduced pressure to complete dryness. The residue was purified by silica gel column chromatography (eluent−EtOAc−hexane (1:2)) to give products *endo*-**8** and *exo’*-**8**.

Methyl(1S*,3S*,3aS*,4R*,9bR*)-3a-nitro-3,4-diphenyl-1,2,3,3a,4,9b-hexahydrochromeno[3,4-c]pyrrole-1-carboxylate (*endo*-**8a**). Yield 120 mg (56%), white powder, mp 183–185 °C. IR (ATR) ν 3381 (NH), 1748 (C=O), 1547, 1340 (NO_2_). ^1^H NMR (500 MHz) *δ* 3.14 (dd, 1H, *J* = 10.8, 7.8 Hz, NH), 4.02 (s, 3H, MeO_2_C), 4.15 (dd, *J* = 7.8, 3.8 Hz, 1H, H-1), 4.79 (d, *J* = 3.8 Hz, 1H, H-9b), 4.97 (d, *J* = 10.8 Hz, 1H, H-3), 5.58 (s, 1H, H-4), 6.82 (d, *J* = 8.2 Hz, 1H, H-6), 7.07 (t, *J* = 7.6 Hz, 1H, H-8), 7.11–7.21 (m, 6H, H-7, H Ph), 7.35–7.45 (m, 2H, H Ph), 7.55 (d, *J* = 7.6 Hz, 1H, H-9); ^13^C NMR (126 MHz) *δ* 46.0, 53.0, 68.6, 70.5, 75.5, 96.7, 118.3, 123.3, 125.1, 126.9 (2C), 128.3 (2C), 128.5 (2C), 128.8, 128.88, 128.90, 129.0 (2C), 129.6, 133.9, 135.1, 149.9, 172.4. HRMS (ESI) *m/z*: [M + H]^+^ calcd for C_25_H_23_N_2_O_5_ 431.1601, found 431.1595.

Methyl(1R*,3S*,3aS*,4R*,9bR*)-3a-nitro-3,4-diphenyl-1,2,3,3a,4,9b-hexahydrochromeno[3,4-c]pyrrole-1-carboxylate (*endo’-***8a**). Yield 50 mg (23%), beige powder, mp 90–92 °C. IR (ATR) ν 3350 (NH), 1735 (C=O), 1542, 1355 (NO_2_). ^1^H NMR (500 MHz) *δ* 2.78 (br. s, 1H, NH), 3.36 (s, 3H, MeO_2_C), 5.00 (d, *J* = 9.8 Hz, 1H, H-1), 5.06 (d, *J* = 9.8 Hz, 1H, H-9b), 5.38 (s, 1H, H-3), 5.68 (s, 1H, H-4), 6.81 (d, *J* = 8.2, 1.2 Hz, 1H, H-6), 6.96 (td, *J* = 7.6, 1.2 Hz, 1H, H-8), 7.11 (ddd, *J* = 8.2, 7.8, 1.4 Hz, 1H, H-7), 7.22–7.34 (m, 11H, H-9, H Ph); ^13^C NMR (126 MHz) *δ* 45.2, 51.7, 64.3, 68.6, 77.5, 97.8, 118.1, 120.6, 121.9, 127.3 (2C), 128.2 (2C), 128.4 (2C), 128.7 (2C), 128.87, 128.92, 129.0, 129.7, 134.8, 136.7, 152.6, 173.3. HRMS (ESI) *m/z*: [M + H]^+^ calcd for C_25_H_23_N_2_O_5_ 431.1601, found 431.1604.

Methyl(1S*,3S*,3aS*,4R*,9bR*)-3-(4-methoxyphenyl)-3a-nitro-4-phenyl-1,2,3,3a,4,9b-hexahydrochromeno[3,4-c]pyrrole-1-carboxylate (endo-**8b**). Yield 145 mg (63%), white powder, mp 159–161 °C. IR (ATR) ν 3323 (NH), 1745 (C=O), 1536, 1361 (NO_2_). ^1^H NMR (600 MHz) δ 3.09 (br. s, 1H, NH), 3.83 (s, 3H, MeO), 4.02 (s, 3H, MeO_2_C), 4.13 (br. s, 1H, H-1), 4.77 (d, *J* = 3.7 Hz, 1H, H-9b), 4.93 (d, *J* = 4.9 Hz, 1H, H-3), 5.53 (s, 1H, H-4), 6.81 (d, *J* = 8.1 Hz, 1H, H-6), 6.95 (d, *J* = 8.6 Hz, 2H, H-3,5 4-MeOC_6_H_4_), 7.06 (td, *J* = 7.5, 0.9 Hz, 1H, H-8), 7.12–7.20 (m, 6H, H-7, H Ph), 7.30 (d, *J* = 8.6 Hz, 2H, H-2,6 4-MeOC_6_H_4_), 7.55 (d, *J* = 7.6 Hz, 1H, H-9); ^13^C NMR (151 MHz) *δ* 45.9 (C-9b), 53.2 (MeO_2_C), 55.3 (MeO), 68.4 (C-1), 70.1 (C-3), 75.5 (C-4), 96.4 (C-3a), 114.4 (C-3,5 4-MeOC_6_H_4_), 118.4 (C-6), 123.3 (C-8), 124.9 (C-9a), 125.3 (C-7), 128.1 (C-2,6 4-MeOC_6_H_4_), 128.3 (C-2,6 Ph), 128.5 (C-3,5 Ph), 128.7 (C-9), 128.9 (C-4 Ph, C-1 4-MeOC_6_H_4_), 135.0 (C-1 Ph), 149.8 (C-5a), 160.6 (C-4 4-MeOC_6_H_4_), 172.3 (C=O). HRMS (ESI) *m/z*: [M + H]^+^ calcd for C_26_H_25_N_2_O_6_ 461.1707, found 461.1710.

Methyl(1R*,3S*,3aS*,4R*,9bR*)-3-(4-methoxyphenyl)-3a-nitro-4-phenyl-1,2,3,3a,4,9b-hexahydrochromeno[3,4-c]pyrrole-1-carboxylate (endo’-**8b**). Yield 48 mg (21%), beige powder, mp 132–133 °C. IR (ATR) ν 3356 (NH), 1732 (C=O), 1543, 1351 (NO_2_). ^1^H NMR (600 MHz) *δ* 2.74 (br. s, 1H, NH), 3.35 (s, 3H, MeO_2_C), 3.78 (s, 3H, MeO), 4.98 (d, *J* = 9.8 Hz, 1H, H-1), 5.04 (d, *J* = 9.8 Hz, 1H, H-9b), 5.35 (s, 1H, H-3), 5.64 (s, 1H, H-4), 6.80 (dd, *J* = 8.2, 1.0 Hz, 1H, H-6), 6.85 (d, *J* = 8.7 Hz, 2H, H-3,5 4-MeOC_6_H_4_), 6.95 (td, *J* = 7.6, 1.0 Hz, 1H, H-8), 7.10 (ddd, *J* = 8.2, 7.6, 1.0 Hz, 1H, H-7), 7.21 (d, *J* = 8.7 Hz, 2H, H-2,6 4-MeOC_6_H_4_), 7.23 (d, *J* = 8.0 Hz, 1H, H-9), 7.25–7.28 (m, 5H, H Ph); ^13^C NMR (151 MHz) *δ* 45.2, 51.9, 55.3, 64.4, 68.6, 77.5, 97.9, 114.2 (2C), 118.3, 120.9, 122.1, 128.3 (2C), 128.36, 128.40, 128.5 (2C), 128.6 (2C), 129.0, 129.8, 134.9, 152.6, 160.3, 173.4. HRMS (ESI) *m/z*: [M + H]^+^ calcd for C_26_H_25_N_2_O_6_ 461.1707, found 461.1706.

Methyl(1S*,3S*,3aS*,4R*,9bR*)-3-(3,4-dimethoxyphenyl)-3a-nitro-4-phenyl-1,2,3,3a,4,9b-hexahydrochromeno[3,4-c]pyrrole-1-carboxylate (endo-**8c**). Yield 132 mg (54%), white powder, mp 191–193 °C. IR (ATR) ν 3327 (NH), 1732 (C=O), 1535, 1341 (NO_2_). ^1^H NMR (500 MHz) *δ* 3.12 (br. s, 1H, NH), 3.90 (s, 3H, MeO), 3.91 (s, 3H, MeO), 4.02 (s, 3H, MeO_2_C), 4.14 (d, *J* = 3.8 Hz, 1H, H-1), 4.77 (d, *J* = 3.8 Hz, 1H, H-9b), 4.92 (c, 1H, H-3), 5.55 (s, 1H, H-4), 6.81 (dd, *J* = 7.5, 1.0 Hz, 1H, H-6), 6.85 (d, *J* = 1.8 Hz, 1H, H-2 (MeO)_2_C_6_H_3_), 6.90 (d, *J* = 8.3 Hz, 1H, H-5 (MeO)_2_C_6_H_3_), 6.95 (dd, *J* = 8.3, 1.8 Hz, 1H, H-6 (MeO)_2_C_6_H_3_), 7.06 (td, *J* = 7.5, 1.0 Hz, 1H, H-8), 7.12–7.21 (m, 6H, H-7, H Ph), 7.54 (d, *J* = 7.5 Hz, 1H, H-9); ^13^C NMR (126 MHz) *δ* 45.9, 53.0, 55.9, 56.1, 68.4, 70.5, 75.7, 96.4, 109.8, 111.3, 118.3, 119.6, 123.2, 125.1, 126.3, 128.3 (2C), 128.5 (2C), 128.8, 128.9 (2C), 135.1, 149.3, 149.9, 150.1, 172.5. HRMS (ESI) *m/z*: [M + H]^+^ calcd for C_27_H_27_N_2_O_6_ 491.1813, found 491.1814.

Methyl (1R*,3S*,3aS*,4R*,9bR*)-3-(3,4-dimethoxyphenyl)-3a-nitro-4-phenyl-1,2,3,3a,4,9b-hexahydrochromeno[3,4-c]pyrrole-1-carboxylate (endo’-**8c**). Yield 66 mg (27%), beige powder, mp 113–115 °C. IR (ATR) ν 3338 (NH), 1732 (C=O), 1536, 1340 (NO_2_). ^1^H NMR (500 MHz) *δ* 2.73 (br. s, 1H, NH), 3.36 (s, 3H, MeO_2_C), 3.80 (s, 3H, MeO), 3.85 (s, 3H, MeO), 4.96 (d, *J* = 9.5 Hz, 1H, H-1), 5.07 (d, *J* = 9.5 Hz, 1H, H-9b), 5.40 (s, 1H, H-3), 5.59 (s, 1H, H-4), 6.71 (d, *J* = 1.8 Hz, 1H, H-2 (MeO)_2_C_6_H_3_), 6.78–6.83 (m, 2H, H-6, H-5 (MeO)_2_C_6_H_3_), 6.90 (dd, *J* = 8.0, 1.8 Hz, 1H, H-6 (MeO)_2_C_6_H_3_), 6.96 (t, *J* = 7.6 Hz, 1H, H-8), 7.10 (t, *J* = 7.8 Hz, 1H, H-7), 7.23–7.31 (m, 6H, H-9, H Ph); ^13^C NMR (126 MHz) *δ* 45.4, 51.7, 55.8, 56.0, 64.0, 68.4, 77.8, 97.9, 110.5, 111.0, 118.2, 119.7, 122.0, 127.9, 128.1 (2C), 128.3, 128.6 (2C), 128.9 (2C), 129.7, 134.9, 149.0, 149.6, 152.6, 173.5. HRMS (ESI) *m/z*: [M + H]^+^ calcd for C_27_H_27_N_2_O_6_ 491.1813, found 491.1806.

Methyl (1S*,3S*,3aS*,4R*,9bR*)-3a-nitro-4-phenyl-3-(3,4,5-trimethoxyphenyl)-1,2,3,3a,4,9b-hexahydrochromeno[3,4-c]pyrrole-1-carboxylate (endo-**8d**). Yield 143 mg (55%), white powder, mp 200–202 °C. IR (ATR) ν 3356 (NH), 1735 (C=O), 1537, 1332 (NO_2_). ^1^H NMR (500 MHz) *δ* 3.02 (br. s, 1H, NH), 3.87 (s, 3H, MeO), 3.89 (s, 6H, MeO), 4.02 (s, 3H, MeO_2_C), 4.13 (d, *J* = 3.8 Hz, 1H, H-1), 4.80 (d, *J* = 3.8 Hz, 1H, H-9b), 4.89 (c, 1H, H-3), 5.59 (s, 1H, H-4), 6.58 (s, 2H, H-2,6 3,4,5-(MeO)_3_C_6_H_2_), 6.82 (d, *J* = 8.1 Hz, 1H, H-6), 7.07 (t, *J* = 7.6 Hz, 1H, H-8), 7.13–7.24 (m, 6H, H-7, H Ph), 7.53 (d, *J* = 7.6 Hz, 1H, H-9); ^13^C NMR (126 MHz) *δ* 45.8, 52.9 (3C), 56.3, 68.1, 70.2, 75.7, 96.3, 104.1 (2C), 118.1, 123.1, 124.7, 128.1 (2C), 128.3 (2C), 128.6, 128.77, 128.80, 129.8, 134.9, 138.9, 149.9, 153.4 (2C), 172.4. HRMS (ESI) *m/z*: [M + H]^+^ calcd for C_28_H_29_N_2_O_8_ 521.1918, found 521.1921.

Methyl (1R*,3S*,3aS*,4R*,9bR*)-3a-nitro-4-phenyl-3-(3,4,5-trimethoxyphenyl)-1,2,3,3a,4,9b-hexahydrochromeno[3,4-c]pyrrole-1-carboxylate (endo’-**8d**). This product was not isolated in pure form. ^1^H NMR (400 MHz) *δ* 3.02 (br. s, 1H, NH), 3.39 (s, 3H, MeO_2_C), 3.77 (s, 6H, MeO), 3.81 (s, 3H, MeO), 4.94 (d, *J* = 9.3 Hz, 1H, H-1), 5.10 (d, *J* = 9.3 Hz, 1H, H-9b), 5.42 (c, 1H, H-3), 5.54 (s, 1H, H-4), 6.46 (s, 2H, H-2,6 3,4,5-(MeO)_3_C_6_H_2_), 6.79 (d, *J* = 8.2 Hz, 1H, H-6), 6.98 (td, *J* = 7.6, 1.1 Hz, 1H, H-8), 7.10 (t, *J* = 8.1 Hz, 1H, H-7), 7.21–7.37 (m, 6H, H-9, H Ph).

Methyl (1S*,3S*,3aS*,4R*,9bR*)-3-(benzo[d][1,3]*dioxol-5-yl)-3a-nitro-4-phenyl-1,2,3,3a,4,9b-hexahydrochromeno[3,4-c]pyrrole-1-carboxylate (endo*-**8e**). Yield 133 mg (56%), white powder, mp 177–179 °C. IR (ATR) ν 3328 (NH), 1744 (C=O), 1536, 1346 (NO_2_). ^1^H NMR (500 MHz) *δ* 3.00 (dd, *J* = 10.2, 7.7 Hz, 1H, NH), 4.01 (s, 3H, MeO_2_C), 4.11 (dd, *J* = 7.7, 3.8 Hz, 1H, H-1), 4.77 (d, *J* = 3.8 Hz, 1H, H-9b), 4.89 (d, *J* = 10.2 Hz, 1H, H-3), 5.57 (s, 1H, H-4), 5.99 (d, *J* = 1.4 Hz, 1H, OCH_2_O), 6.00 (d, *J* = 1.4 Hz, 1H, OCH_2_O), 6.80 (dd, *J* = 8.1, 1.1 Hz, 1H, H-6), 6.85 (d, *J* = 8.0 Hz, 1H, H-7 benzo[*d*][1,3]dioxol-5-yl), 6.86 (d, *J* = 1.6 Hz, 1H, H-4 benzo[*d*][1,3]dioxol-5-yl), 6.88 (dd, *J* = 8.1, 1.6 Hz, 1H, H-6 benzo[*d*][1,3]dioxol-5-yl), 7.06 (td, *J* = 7.6, 1.1 Hz, 1H, H-8), 7.11–7.22 (m, 6H, H-7, H Ph), 7.53 (d, *J* = 7.6 Hz, 1H, H-9); ^13^C NMR (126 MHz) *δ* 45.8, 53.0, 68.3, 70.2, 75.5, 96.3, 101.4, 107.0, 108.6, 118.3, 120.8, 123.2, 125.1, 127.6, 128.3 (2C), 128.5 (2C), 128.8, 128.9 (2C), 135.1, 148.3, 148.7, 149.9, 172.4. HRMS (ESI) *m/z*: [M + H]^+^ calcd for C_26_H_23_N_2_O_7_ 475.1500, found 475.1486.

Methyl (1R*,3S*,3aS*,4R*,9bR*)-3-(benzo[d][1,3]*dioxol-5-yl)-3a-nitro-4-phenyl-1,2,3,3a,4,9b-hexahydrochromeno[3,4-c]pyrrole-1-carboxylate (endo’*-**8e**). Yield 47 mg (20%), beige powder, mp 111–113 °C. IR (ATR) ν 3362 (NH), 1731 (C=O), 1543, 1340 (NO_2_). ^1^H NMR (500 MHz) *δ* 2.69 (br. s, 1H, NH), 3.36 (s, 3H, MeO_2_C), 4.95 (d, *J* = 9.7 Hz, 1H, H-1), 5.04 (d, *J* = 9.7 Hz, 1H, H-9b), 5.32 (br. s, 1H, H-3), 5.64 (s, 1H, H-4), 5.94 (d, *J* = 1.3 Hz, 1H, OCH_2_O), 5.95 (d, *J* = 1.3 Hz, 1H, OCH_2_O), 6.73–6.82 (m, 4H, H-6, H-4,6,7 benzo[*d*][1,3]dioxol-5-yl), 6.96 (td, *J* = 7.5, 1.0 Hz, 1H, H-8), 7.10 (td, *J* = 7.6, 1.2 Hz, 1H, H-8), 7.22 (dd, *J* = 7.6, 1.2 Hz, 1H, H-9), 7.25–7.29 (m, 5H, H Ph); ^13^C NMR (126 MHz) *δ* 45.0, 51.7, 64.1, 68.4, 77.5, 97.6, 101.2, 107.5, 108.3, 118.2, 120.7, 121.2, 121.9, 128.2 (2C), 128.4 (2C), 128.87, 128.92, 129.7, 130.4, 134.7, 147.9, 148.2, 152.5, 173.4. HRMS (ESI) *m/z*: [M + H]^+^ calcd for C_26_H_23_N_2_O_7_ 475.1500, found 475.1503.

Methyl(1S*,3S*,3aS*,4R*,9bR*)-8-bromo-3-(4-methoxyphenyl)-3a-nitro-4-phenyl-1,2,3,3a,4,9b-hexahydrochromeno[3,4-c]pyrrole-1-carboxylate (endo-**8f**). Yield 164 mg (61%), white powder, mp 208–210 °C. IR (ATR) ν 3359 (NH), 1755 (C=O), 1547, 1362 (NO_2_). ^1^H NMR (500 MHz) *δ* 3.07 (dd, *J* = 10.6, 7.7 Hz, 1H, NH), 3.82 (s, 3H, MeO), 4.03 (s, 3H, MeO_2_C), 4.10 (dd, *J* = 7.7, 3.8 Hz, 1H, H-1), 4.74 (d, *J* = 3.8 Hz, 1H, H-9b), 4.88 (d, *J* = 10.6 Hz, 1H, H-3), 5.53 (s, 1H, H-4), 6.69 (d, *J* = 8.7 Hz, 1H, H-6), 6.94 (d, *J* = 8.7 Hz, 2H, H-3,5 4-MeOC_6_H_4_), 7.11–7.15 (m, 2H, H Ph), 7.16–7.22 (m, 3H, H Ph), 7.24 (dd, *J* = 8.7, 2.5 Hz, 1H, H-7), 7.28 (d, *J* = 8.7 Hz, 2H, H-2,6 4-MeOC_6_H_4_), 7.67 (d, *J* = 2.5 Hz, 1H, H-9); ^13^C NMR (126 MHz) *δ* 45.8, 53.2, 55.3, 68.3, 70.3, 75.7, 96.0, 114.4 (2C), 115.4, 120.2, 125.4, 127.3, 128.0 (2C), 128.2 (2C), 128.6 (2C), 129.0, 131.4, 131.9, 134.7, 149.1, 160.6, 172.1. HRMS (ESI) *m/z*: [M + H]^+^ calcd for C_26_H_24_BrN_2_O_6_ 539.0812, found 539.0809.

Methyl(1R*,3S*,3aS*,4R*,9bR*)-8-bromo-3-(4-methoxyphenyl)-3a-nitro-4-phenyl-1,2,3,3a,4,9b-hexahydrochromeno[3,4-c]pyrrole-1-carboxylate (endo’-**8f**). Yield 57 mg (21%), beige powder, mp 188–190 °C. IR (ATR) ν 3382 (NH), 1715 (C=O), 1546, 1362 (NO_2_). ^1^H NMR (500 MHz) *δ* 2.72 (br. s, 1H, NH), 3.47 (s, 3H, MeO_2_C), 3.78 (s, 3H, MeO), 4.96 (d, *J* = 9.8 Hz, 1H, H-1), 4.99 (d, *J* = 9.8 Hz, 1H, H-9b), 5.29 (s, 1H, H-3), 5.63 (s, 1H, H-4), 6.70 (d, *J* = 8.7 Hz, 1H, H-6), 6.85 (d, *J* = 8.7 Hz, 2H, H-3,5 4-MeOC_6_H_4_), 7.21 (d, *J* = 8.7 Hz, 2H, H-2,6 4-MeOC_6_H_4_), 7.23–7.29 (m, 7H, H-7,9, Ph); ^13^C NMR (126 MHz) *δ* 44.7, 51.9, 55.2, 63.9, 68.3, 77.5, 97.2, 114.1 (2C), 120.0, 122.9, 124.3, 128.26 (2C), 128.32 (2C), 128.5 (2C), 129.0, 131.6, 131.8, 132.3, 134.4, 151.2, 160.0, 170.4. HRMS (ESI) *m/z*: [M + H]^+^ calcd for C_26_H_24_BrN_2_O_6_ 539.0812, found 539.0810.

Methyl (1S*,3S*,3aS*,4R*,9bR*)-8-methoxy-3-(4-methoxyphenyl)-3a-nitro-4-phenyl-1,2,3,3a,4,9b-hexahydrochromeno[3,4-c]pyrrole-1-carboxylate (endo-**8g**). Yield 140 mg (57%), white powder, mp 173–175 °C. IR (ATR) ν 3364 (NH), 1752 (C=O), 1544, 1365 (NO_2_). ^1^H NMR (400 MHz) *δ* 3.09 (t, *J* = 8.7 Hz, 1H, NH), 3.79 (s, 3H, MeO), 3.82 (s, 3H, MeO), 4.02 (s, 3H, MeO_2_C), 4.12 (dd, *J* = 6.7, 3.9 Hz, 1H, H-1), 4.72 (d, *J* = 3.9 Hz, 1H, H-9b), 4.93 (d, *J* = 10.4 Hz, 1H, H-3), 5.50 (s, 1H, H-4), 6.69 (dd, *J* = 8.8, 2.5 Hz, 1H, H-7), 6.72 (d, *J* = 8.8 Hz, 1H, H-6), 6.94 (d, *J* = 8.7 Hz, 2H, H-3,5 4-MeOC_6_H_4_), 7.07 (d, *J* = 2.5 Hz, 1H, H-9), 7.11–7.21 (m, 5H, H Ph), 7.29 (d, *J* = 8.7 Hz, 2H, H-2,6 4-MeOC_6_H_4_); ^13^C NMR (126 MHz) *δ* 46.4, 53.0, 55.2, 55.6, 68.4, 70.3, 75.7, 96.7, 113.1, 114.4 (2C), 114.7, 119.1, 125.6, 126.0, 128.0 (2C), 128.3 (2C), 128.4 (2C), 128.8, 135.1, 143.6, 155.3, 160.6, 172.3. HRMS (ESI) *m/z*: [M + H]^+^ calcd for C_27_H_27_N_2_O_7_ 491.1813, found 491.1812.

Methyl (1R*,3S*,3aS*,4R*,9bR*)-8-methoxy-3-(4-methoxyphenyl)-3a-nitro-4-phenyl-1,2,3,3a,4,9b-hexahydrochromeno[3,4-c]pyrrole-1-carboxylate (endo’-**8g**). Yield 54 mg (22%), beige powder, mp 95–97 °C. IR (ATR) ν 3344 (NH), 1733 (C=O), 1542, 1358 (NO_2_). ^1^H NMR (500 MHz) *δ* 2.73 (br. s, 1H, NH), 3.40 (s, 3H, MeO_2_C), 3.76 (s, 3H, MeO), 3.79 (s, 3H, MeO), 4.97 (d, *J* = 9.7 Hz, 1H, H-1), 5.01 (d, *J* = 9.7 Hz, 1H, H-9b), 5.36 (s, 1H, H-3), 5.59 (s, 1H, H-4), 6.66 (dd, *J* = 8.8, 2.8 Hz, 1H, H-7), 6.71 (d, *J* = 8.8 Hz, 1H, H-6), 6.75 (d, *J* = 2.8 Hz, 1H, H-9), 6.86 (d, *J* = 8.7 Hz, 2H, H-3,5 4-MeOC_6_H_4_), 7.20–7.26 (m, 7H, H-2,6 4-MeOC_6_H_4_, H Ph); ^13^C NMR (126 MHz) *δ* 45.5, 51.7, 55.2, 55.7, 64.3, 68.6, 77.4, 98.0, 114.0, 114.1 (2C), 115.0, 118.9, 121.6, 128.2 (2C), 128.36 (2C), 128.44 (2C), 128.8, 130.1, 134.9, 146.2, 154.4, 160.2, 173.2. HRMS (ESI) *m/z*: [M + H]^+^ calcd for C_27_H_27_N_2_O_7_ 491.1813, found 491.1811.

### 3.3. Synthesis of Compounds ***9a–j***

General procedure. A mixture of the appropriate 3-nitro-2-(trifluoromethyl)-2*H*-chromene **4** (0.5 mmol), azomethine **5** (0.55 mmol), Et_3_N (7 μL, 5 mg, 0.05 mmol) and AgOAc (5.8 mg, 0.05 mmol) was stirred in dichloromethane (2 mL) for 5 h at room temperature (TLC control, EtOAc−hexane (1:3)). Upon completion of the reaction, the residue was evaporated under reduced pressure to complete dryness. The residue was purified by silica gel column chromatography (eluent−EtOAc−hexane (1:3)) to give products *endo*-**9**.

Methyl (1S*,3S*,3aS*,4S*,9bR*)-3a-nitro-3-phenyl-4-(trifluoromethyl)-1,2,3,3a,4,9b-hexahydrochromeno[3,4-c]pyrrole-1-carboxylate (endo-**9a**). Yield 190 mg (90%), beige powder, mp 125–127 °C. IR (ATR) ν 3320 (NH), 1743 (C=O), 1546, 1361 (NO_2_). ^1^H NMR (500 MHz) *δ* 3.13 (dd, *J* = 11.3, 7.5 Hz, 1H, NH), 4.02 (s, 3H, MeO_2_C), 4.09 (dd, *J* = 7.5, 3.1 Hz, 1H, H-1), 4.57 (d, *J* = 3.1 Hz, 1H, H-9b), 4.83 (d, *J* = 11.3 Hz, 1H, H-3), 5.11 (q, *J* = 7.0 Hz, 1H, H-4), 7.07 (dd, *J* = 8.2, 1.0 Hz, 1H, H-6), 7.18 (ddd, *J* = 8.2, 7.6, 1.0 Hz, 1H, H-8), 7.23–7.32 (m, 3H, H-7, H Ph), 7.43–7.47 (m, 3H, H Ph), 7.52 (d, *J* = 7.7 Hz, 1H, H-9); ^19^F NMR (471 MHz) *δ* 96.6 (d, *J* = 7.0 Hz, CF_3_); ^13^C NMR (126 MHz) *δ* 45.6, 53.1, 68.4, 70.2, 72.3 (q, ^2^*J*_CF_ = 31.8 Hz, C-4), 93.6, 117.6, 123.3 (q, ^1^*J*_CF_ = 288.9 Hz, CF_3_), 124.0, 124.4, 126.5 (2C), 129.09, 129.14, 129.2 (2C), 130.0, 132.6, 149.0, 171.9. HRMS (ESI) *m/z*: [M + H]^+^ calcd for C_20_H_18_F_3_N_2_O_5_ 423.1162, found 423.1160.

Methyl (1S*,3S*,3aS*,4S*,9bR*)-3-(4-methoxyphenyl)-3a-nitro-3-phenyl-4-(trifluoromethyl)-1,2,3,3a,4,9b-hexahydrochromeno[3,4-c]pyrrole-1-carboxylate (endo-**9b**). Yield 208 mg (92%), white powder, mp 163–165 °C. IR (ATR): ν 3321 (NH), 1742 (C=O), 1547, 1362 (NO_2_). ^1^H NMR (500 MHz) *δ* 3.07 (dd, *J* = 11.2, 7.6 Hz, 1H, NH), 3.83 (s, 3H, MeO), 4.02 (s, 3H, MeO_2_C), 4.07 (dd, *J* = 7.6, 3.0 Hz, 1H, H-1), 4.54 (d, *J* = 3.0 Hz, 1H, H-9b), 4.79 (d, *J* = 11.2 Hz, 1H, H-3), 5.07 (q, *J* = 7.0 Hz, 1H, H-4), 6.95 (d, *J* = 8.7 Hz, 2H, H-3,5 4-MeOC_6_H_4_), 7.06 (dd, *J* = 8.1, 1.0 Hz, 1H, H-6), 7.15 (td, *J* = 7.6, 1.0 Hz, 1H, H-8), 7.22 (d, *J* = 8.7 Hz, 2H, H-2,6 4-MeOC_6_H_4_), 7.28 (td, *J* = 8.1, 1.0 Hz, 1H, H-7), 7.51 (dd, *J* = 7.6, 1.0 Hz, 1H, H-9); ^19^F NMR (471 MHz) δ 96.7 (d, *J* = 7.0 Hz, CF_3_); ^13^C NMR (126 MHz) *δ* 45.5, 53.1, 55.3, 68.4, 70.0, 72.4 (q, ^2^*J*_CF_ = 31.4 Hz, C-4), 93.5, 114.6 (2C), 117.5, 123.4 (q, ^1^*J*_CF_ = 288.7 Hz, CF_3_), 124.0, 124.4 (2C), 127.7 (2C), 129.1 (2C), 149.0, 160.9, 172.0. HRMS (ESI) *m/z*: [M + H]^+^ calcd for C_21_H_20_F_3_N_2_O_6_ 453.1268, found 453.1272.

Methyl (1R*,3S*,3aS*,4S*,9bR*)-3-(4-methoxyphenyl)-3a-nitro-3-phenyl-4-(trifluoromethyl)-1,2,3,3a,4,9b-hexahydrochromeno[3,4-c]pyrrole-1-carboxylate (endo’-**9b**). This product was obtained according to the general procedure at −20 °C for 5 h and was not isolated in pure form. ^1^H NMR (400 MHz) *δ* 2.67 (br. s, 1H), 3.36 (s, 3H, MeO_2_C), 3.83 (s, 3H, MeO), 4.83 (d, *J* = 9.0 Hz, 1H, H-1), 4.90 (d, *J* = 9.0 Hz, 1H, H-9b), 5.13 (q, *J* = 7.0 Hz, 1H, H-4), 5.30 (s, 1H, H-3), 6.94 (d, *J* = 8.7 Hz, 2H, H-3,5 4-MeOC_6_H_4_), 6.98 (dd, *J* = 8.0, 1.0 Hz, 1H, H-6), 7.02–7.08 (m, 2H, H-7,8), 7.15 (dd, *J* = 7.6, 1.4 Hz, 1H, H-9), 7.27 (d, *J* = 8.7 Hz, 2H, H-2,6 4-MeOC_6_H_4_); ^19^F NMR (376 MHz) *δ* 98.0 (d, *J* = 7.0 Hz, CF_3_).

Methyl (1R*,3R*,3aS*,4S*,9bR*)-3-(4-methoxyphenyl)-3a-nitro-3-phenyl-4-(trifluoromethyl)-1,2,3,3a,4,9b-hexahydrochromeno[3,4-c]pyrrole-1-carboxylate (exo-**9b**). This product was obtained according to the general procedure without AgOAc and was not isolated in pure form. ^1^H NMR (400 MHz) *δ* 3.02 (dd, *J* = 10.8, 10.0 Hz, 1H, NH), 3.74 (s, 3H, CO_2_Me), 3.87 (s, 3H, MeO), 4.07 (dd, *J* = 10.0, 5.8 Hz, 1H, H-1), 4.38 (d, *J* = 5.8 Hz, 1H, H-9b), 4.51 (d, *J* = 10.8 Hz, 1H, H-3), 4.67 (q, *J* = 7.2 Hz, 1H, H-4) (other signals overlapped with signals of major isomers). ^19^F NMR (471 MHz) *δ* 95.8 (d, *J* = 7.2 Hz, CF_3_).

Methyl (1S*,3S*,3aS*,4S*,9bR*)-3-(3,4-dimethoxyphenyl)-3a-nitro-3-phenyl-4-(trifluoromethyl)-1,2,3,3a,4,9b-hexahydrochromeno[3,4-c]pyrrole-1-carboxylate (endo-**9c**). Yield 222 mg (92%), white powder, mp 150–152 °C. IR (ATR) ν 3386 (NH), 1745 (C=O), 1547, 1363 (NO_2_). ^1^H NMR (400 MHz) *δ* 3.07 (dd, *J* = 10.8, 7.5 Hz, 1H, NH), 3.83 (s, 6H, 2MeO), 4.03 (s, 3H, MeO_2_C), 4.07 (dd, *J* = 7.5, 3.0 Hz, 1H, H-1), 4.55 (d, *J* = 3.0 Hz, 1H, H-9b), 4.78 (d, *J* = 10.8 Hz, 1H, H-3), 5.10 (q, *J* = 7.0 Hz, 1H, H-4), 6.76 (d, *J* = 1.8 Hz, 1H, H-2 3,4-(MeO)_2_C_6_H_3_), 6.87 (dd, *J* = 8.3, 1.8 Hz, 1H, H-6 3,4-(MeO)_2_C_6_H_3_), 6.91 (d, *J* = 8.3 Hz, H-5 3,4-(MeO)_2_C_6_H_3_), 7.06 (dd, *J* = 8.2, 1.0 Hz, 1H, H-6), 7.17 (td, *J* = 7.6, 1.0 Hz, 1H, H-8), 7.28 (td, *J* = 8.2, 1.0 Hz, 1H, H-7), 7.51 (dd, *J* = 7.6, 1.0 Hz, 1H, H-9); ^19^F NMR (376 MHz) *δ* 96.9 (d, *J* = 7.0 Hz, CF_3_); ^13^C NMR (126 MHz) *δ* 45.4, 53.1, 55.9, 56.1, 68.3, 70.3, 72.4 (q, ^2^*J*_CF_ = 31.5 Hz, C-4), 93.4, 109.3, 111.5, 117.6, 119.3, 123.4 (q, ^1^*J*_CF_ = 288.9 Hz, CF_3_), 124.0, 124.4, 124.9, 129.08, 129.11, 149.0, 149.6, 150.4, 172.0. HRMS (ESI) *m/z*: [M + H]^+^ calcd for C_22_H_22_F_3_N_2_O_7_ 483.1374, found 483.1374.

Methyl (1S*,3S*,3aS*,4S*,9bR*)-3a-nitro-4-(trifluoromethyl)-3-(3,4,5-trimethoxyphenyl)-1,2,3,3a,4,9b-hexahydrochromeno[3,4-c]pyrrole-1-carboxylate (endo-**9d**). Yield 218 mg (85%), white powder, mp 115–117 °C. IR (ATR) ν 3326 (NH), 1747 (C=O), 1549, 1334 (NO_2_). ^1^H NMR (400 MHz) *δ* 3.01 (dd, *J* = 10.4, 7.2 Hz, 1H, NH), 3.88 (s, 3H, MeO), 3.89 (s, 6H, 2MeO), 4.03 (s, 3H, MeO_2_C), 4.07 (dd, *J* = 7.2, 3.1 Hz, 1H, H-1), 4.56 (d, *J* = 3.1 Hz, 1H, H-9b), 4.75 (d, *J* = 10.4 Hz, 1H, H-3), 5.13 (q, *J* = 7.0 Hz, 1H, H-4), 6.50 (s, 2H, H-2,6 3,4,5-(MeO)_2_C_6_H_2_), 7.07 (dd, *J* = 8.2, 1.2 Hz, 1H, H-6), 7.18 (td, *J* = 7.7, 1.2 Hz, 1H, H-8), 7.29 (td, *J* = 8.2, 1.0 Hz, 1H, H-7), 7.51 (dd, *J* = 7.7, 1.0 Hz, 1H, H-9); ^19^F NMR (376 MHz) *δ* 96.9 (d, *J* = 7.0 Hz, CF_3_); ^13^C NMR (126 MHz) *δ* 45.3, 53.1, 56.4 (2C), 60.9, 68.3, 70.5, 72.5 (q, ^2^*J*_CF_ = 31.6 Hz, C-4), 93.3, 103.8 (2C), 117.6, 123.3 (q, ^1^*J*_CF_ = 288.8 Hz, CF_3_), 124.0, 124.5, 128.2, 129.1, 129.2, 149.0, 153.8 (3C), 172.0. HRMS (ESI) *m/z*: [M + H]^+^ calcd for C_23_H_24_F_3_N_2_O_8_ 513.1479, found 513.1472.

Methyl (1S*,3S*,3aS*,4S*,9bR*)-3-(benzo[d][1,3]*dioxol-5-yl)-3a-nitro-4-(trifluoromethyl)-1,2,3,3a,4,9b-hexahydrochromeno[3,4-c]pyrrole-1-carboxylate (endo*-**9e**). Yield 203 mg (87%), white powder, mp 168–170 °C. IR (ATR) ν 3336 (NH), 1736 (C=O), 1544, 1362 (NO_2_). ^1^H NMR (400 MHz) *δ* 2.97 (dd, *J* = 10.7, 7.6 Hz, 1H, NH), 4.01 (s, 3H, MeO_2_C), 4.05 (dd, *J* = 7.6, 3.0 Hz, 1H, H-1), 4.55 (d, *J* = 3.0 Hz, 1H, H-9b), 4.75 (d, *J* = 10.7 Hz, 1H, H-3), 5.11 (q, *J* = 7.0 Hz, 1H, H-4), 6.01 (d, *J* = 1.4 Hz, 1H, OCH_2_O), 6.02 (d, *J* = 1.4 Hz, 1H, OCH_2_O), 6.76–6.82 (m, 2H, H-4,7 benzo[*d*][1,3]dioxol-5-yl), 6.85 (d, *J* = 7.9 Hz, 1H, H-6 benzo[*d*][1,3]dioxol-5-yl), 7.05 (dd, *J* = 8.2, 1.1 Hz, 1H, H-6), 7.17 (td, *J* = 7.7, 1.1 Hz, 1H, H-8), 7.27 (td, *J* = 8.2, 1.1 Hz, 1H, H-7), 7.50 (d, *J* = 7.7, 1.1 Hz, 1H, H-9); ^19^F NMR (376 MHz) *δ* 96.7 (d, *J* = 7.0 Hz, CF_3_); ^13^C NMR (126 MHz) *δ* 45.3, 53.1, 68.2, 70.0, 72.3 (q, ^2^*J*_CF_ = 31.4 Hz, C-4), 93.3, 101.6, 106.6, 108.8, 117.6, 120.5, 123.4 (q, ^1^*J*_CF_ = 289.0 Hz, CF_3_), 124.0, 124.4, 126.3, 129.09, 129.13, 148.5, 148.99, 149.02, 171.9. HRMS (ESI) *m/z*: [M + H]^+^ calcd for C_21_H_18_F_3_N_2_O_7_ 467.1061, found 467.1064.

Methyl (1S*,3S*,3aS*,4S*,9bR*)-8-chloro-3-(4-methoxyphenyl)-3a-nitro-4-(trifluoromethyl)-1,2,3,3a,4,9b-hexahydrochromeno[3,4-c]pyrrole-1-carboxylate (endo-**9f**). Yield 219 mg (90%), white powder, mp 191–193 °C. IR (ATR) ν 3316 (NH), 1745 (C=O), 1547, 1360 (NO_2_). ^1^H NMR (500 MHz) *δ* 3.06 (dd, *J* = 11.0, 7.7 Hz, 1H, NH), 3.83 (s, 3H, MeO), 4.01–4.05 (m, 4H, MeO_2_C, H-1), 4.50 (d, *J* = 3.3 Hz, 1H, H-9b), 4.74 (d, *J* = 11.0 Hz, 1H, H-3), 5.07 (q, *J* = 6.9 Hz, 1H, H-4), 6.95 (d, *J* = 8.6 Hz, 2H, H-3,5 4-MeOC_6_H_4_), 7.01 (d, *J* = 8.7 Hz, 1H, H-6), 7.20 (d, *J* = 8.6 Hz, 2H, H-2,6 4-MeOC_6_H_4_), 7.24 (dd, *J* = 8.7, 2.3 Hz, 1H, H-7), 7.49 (d, *J* = 2.3 Hz, 1H, H-9); ^19^F NMR (471 MHz) *δ* 96.8 (d, *J* = 6.9 Hz, CF_3_); ^13^C NMR (126 MHz, CDCl_3_) *δ* 45.3, 53.3, 55.3, 68.1, 70.1, 72.5 (q, ^2^*J*_CF_ = 31.7 Hz, C-4), 93.1, 114.7 (2C), 119.1, 123.2 (q, ^1^*J*_CF_ = 288.9 Hz, CF_3_), 124.1, 125.8, 127.7 (2C), 128.7, 129.4, 129.5, 147.7, 161.0, 171.6. HRMS (ESI) *m/z*: [M + H]^+^ calcd for C_21_H_19_ClF_3_N_2_O_6_ 487.0878, found 487.0879.

Methyl (1S*,3S*,3aS*,4S*,9bR*)-8-bromo-3-(4-methoxyphenyl)-3a-nitro-4-(trifluoromethyl)-1,2,3,3a,4,9b-hexahydrochromeno[3,4-c]pyrrole-1-carboxylate (endo-**9g**). Yield 250 mg (94%), white powder, mp 191–192 °C. IR (ATR) ν 3315 (NH), 1746 (C=O), 1546, 1361 (NO_2_). ^1^H NMR (500 MHz) *δ* 3.06 (br. s, 1H, NH), 3.83 (s, 3H, MeO), 4.01–4.05 (m, 4H, MeO_2_C, H-1), 4.50 (d, *J* = 3.3 Hz, 1H, H-9b), 4.73 (s, 1H, H-3), 5.07 (q, *J* = 6.9 Hz, 1H, H-4), 6.94 (d, *J* = 8.7 Hz, 2H, H-3,5 4-MeOC_6_H_4_), 6.97 (d, *J* = 8.8 Hz, 1H, H-6), 7.20 (d, *J* = 8.7 Hz, 2H, H-2,6 4-MeOC_6_H_4_), 7.38 (dd, *J* = 8.8, 2.2 Hz, 1H, H-7), 7.63 (d, *J* = 2.2 Hz, 1H, H-9); ^19^F NMR (471 MHz) *δ* 96.8 (d, *J* = 6.9 Hz, CF_3_); ^13^C NMR (126 MHz) *δ* 45.2, 53.3, 55.3, 68.1, 70.1, 72.4 (q, ^2^*J*_CF_ = 31.7 Hz, C-4), 93.0, 114.6 (2C), 116.8, 119.4, 123.2 (q, ^1^*J*_CF_ = 288.9 Hz, CF_3_), 124.0, 126.2, 127.7 (2C), 131.7, 132.3, 148.2, 160.9, 171.6. HRMS (ESI) *m/z*: [M + H]^+^ calcd for C_21_H_19_BrF_3_N_2_O_6_ 531.0373, found 531.0374.

Methyl (1S*,3S*,3aS*,4S*,9bR*)-6,8-dibromo-3-(4-methoxyphenyl)-3a-nitro-4-(trifluoromethyl)-1,2,3,3a,4,9b-hexahydrochromeno[3,4-c]pyrrole-1-carboxylate (endo-**9h**). Yield 284 mg (93%), white powder, mp 189–191 °C. IR (ATR) ν 3321 (NH), 1750 (C=O), 1547, 1362 (NO_2_). ^1^H NMR (400 MHz) *δ* 3.07 (dd, *J* = 10.8, 7.7 Hz, 1H, NH), 3.80 (s, 3H, MeO), 4.00 (dd, *J* = 7.7, 3.2 Hz, 1H, H-1), 4.03 (s, 3H, MeO_2_C), 4.53 (d, *J* = 3.2 Hz, 1H, H-9b), 4.72 (d, *J* = 10.8 Hz, 1H, H-3), 5.18 (q, *J* = 6.8 Hz, 1H, H-4), 6.96 (d, *J* = 8.6 Hz, 2H, H-3,5 4-MeOC_6_H_4_), 7.22 (d, *J* = 8.6 Hz, 2H, H-2,6 4-MeOC_6_H_4_), 7.61 (d, *J* = 1.9 Hz, 1H, H-9), 7.67 (d, *J* = 1.9 Hz, 1H, H-7); ^19^F NMR (376 MH) δ 97.0 (d, *J* = 6.8 Hz, CF_3_); ^13^C NMR (126 MHz) *δ* 45.5, 53.5, 55.3, 68.1, 70.3, 73.1 (q, ^2^*J*_CF_ = 32.2 Hz, C-4), 93.2, 112.9 114.8 (2C), 116.9, 122.9 (q, ^1^*J*_CF_ = 288.5 Hz, CF_3_), 123.8, 127.5, 127.7 (2C), 130.9, 135.3, 145.5, 161.1, 171.3. HRMS (ESI) *m/z*: [M + H]^+^ calcd for C_21_H_18_Br_2_F_3_N_2_O_6_ 608.9478, found 608.9475.

Methyl (1S*,3S*,3aS*,4S*,9bR*)-8-methoxy-3-(4-methoxyphenyl)-3a-nitro-4-(trifluoromethyl)-1,2,3,3a,4,9b-hexahydrochromeno[3,4-c]pyrrole-1-carboxylate (endo-**9i**). Yield 215 mg (89%), white powder, mp 125–127 °C. IR (ATR) ν 3368 (NH), 1744 (C=O), 1547, 1348 (NO_2_). ^1^H NMR (400 MHz) *δ* 3.07 (dd, *J* = 11.3, 7.8 Hz, 1H, NH), 3.82 (s, 3H, MeO), 3.83 (s, 3H, MeO), 4.02 (s, 3H, MeO_2_C), 4.07 (dd, *J* = 7.8, 3.2 Hz, 1H, H-1), 4.49 (d, *J* = 3.2 Hz, 1H, H-9b), 4.78 (d, *J* = 11.3 Hz, 1H, H-3), 5.03 (q, *J* = 7.0 Hz, 1H, H-4), 6.83 (dd, *J* = 9.0, 2.8 Hz, 1H, H-7), 6.95 (d, *J* = 8.7 Hz, 2H, H-3,5 4-MeOC_6_H_4_), 6.98 (d, *J* = 9.0 Hz, 1H, H-6), 7.01 (d, *J* = 2.2 Hz, 1H, H-9), 7.21 (d, *J* = 8.7 Hz, 2H, H-2,6 4-MeOC_6_H_4_); ^19^F NMR (376 MHz) *δ* 97.0 (d, *J* = 7.0 Hz, CF_3_); ^13^C NMR (126 MHz) *δ* 45.9, 53.1, 55.3, 55.7, 68.3, 70.1, 72.6 (q, ^2^*J*_CF_ = 31.4 Hz, C-4), 93.8, 113.1, 114.6 (2C), 115.2, 118.4, 123.4 (q, ^1^J_CF_ = 289.2 Hz, CF_3_), 124.4, 124.8, 127.7 (2C), 142.8, 156.2, 160.9, 171.9. HRMS (ESI) *m/z*: [M + H]^+^ calcd for C_22_H_22_F_3_N_2_O_7_ 483.1374, found 483.1374.

Methyl (1S*,3S*,3aS*,4S*,9bR*)-6-ethoxy-3-(4-methoxyphenyl)-3a-nitro-4-(trifluoromethyl)-1,2,3,3a,4,9b-hexahydrochromeno[3,4-c]pyrrole-1-carboxylate (endo-**9j**). Yield 216 mg (87%), white powder, mp 123–125 °C. IR (ATR) ν 3348 (NH), 1752 (C=O), 1557, 1361 (NO_2_). ^1^H NMR (400 MHz) *δ* 1.48 (t, *J* = 7.0 Hz, Me), 3.06 (dd, *J* = 10.9, 7.6 Hz, 1H, NH), 3.83 (s, 3H, MeO), 4.01 (s, 3H, MeO_2_C), 4.05 (dd, *J* = 7.6, 3.2 Hz, 1H, H-1), 4.17 (d, *J* = 7.0 Hz, OCH_2_), 4.52 (d, *J* = 3.2 Hz, 1H, H-9b), 4.81 (d, *J* = 10.9 Hz, 1H, H-3), 5.19 (q, *J* = 7.0 Hz, 1H, H-4), 6.82–6.89 (m, 1H, H-8), 6.94 (d, *J* = 8.7 Hz, 2H, H-3,5 4-MeOC_6_H_4_), 7.06–7.10 (m, 2H, H-7,9), 7.25 (d, *J* = 8.7 Hz, 2H, H-2,6 4-MeOC_6_H_4_); ^19^F NMR (376 MHz) *δ* 97.1 (d, *J* = 7.0 Hz, CF_3_); ^13^C NMR (126 MHz) *δ* 14.7, 45.7, 53.1, 55.3, 64.7, 68.4, 70.2, 72.7 (q, ^2^*J*_CF_ = 31.5 Hz, C-4), 94.0, 112.3, 114.6 (2C), 120.1, 123.3 (q, ^1^*J*_CF_ = 288.8 Hz, CF_3_), 124.3, 124.4, 125.3, 127.8 (2C), 139.1, 148.1, 160.9, 172.0. HRMS (ESI) *m/z*: [M + H]^+^ calcd for C_23_H_23_F_3_N_2_NaO_7_ 519.1350, found 519.1347.

### 3.4. Synthesis of Compounds ***10a–g***

General procedure. A mixture of the appropriate 3-nitro-2-(trichloromethyl)-2*H*-chromene **4** (0.5 mmol), azomethine **5** (0.55 mmol), Et_3_N (7 μL, 5 mg, 0.05 mmol) and AgOAc (5.8 mg, 0.05 mmol) was stirred in dichloromethane (2 mL) for 5 h at room temperature (TLC control, EtOAc−hexane (1:3)). Upon completion of the reaction, the residue was evaporated under reduced pressure to complete dryness. The residue was purified by by silica gel column chromatography using (eluent−EtOAc−hexane (1:3)) to give products **10** as white powders.

Methyl (S)-2-[((E)-benzylidene)amino]-2-((2S*,3R*,4R*)-3-nitro-2-(trichloromethyl)chroman-4-yl)acetate (**10a**). Yield 101 mg (43%), mp 225–227 °C. IR (ATR) ν 1737 (C=O), 1552, 1311 (NO_2_). ^1^H NMR (400 MHz) *δ* 3.86 (s, 3H, MeO_2_C), 4.14 (br. d, *J* = 2.3 Hz, 1H, H-4*’*), 4.53 (d, *J* = 2.3 Hz, 1H, H-2), 5.22 (d, *J* = 1.4 Hz, 1H, H-2*’*), 6.29 (br. d, *J* = 1.4 Hz, 1H, H-3*’*), 7.04 (d, *J* = 8.0 Hz, 1H, H-8*’*), 7.09 (t, *J* = 7.5 Hz, 1H, H-6*’*), 7.20–7.29 (m, 2H, H-5*’*,7*’*), 7.39 (t, *J* = 7.3 Hz, 2H, H Ph), 7.46 (tt, *J* = 7.3, 1.3 Hz, 1H, H Ph), 7.63 (dd, *J* = 7.3, 1.3 Hz, 2H, H Ph), 8.02 (s, 1H, =CH); ^13^C NMR (126 MHz) *δ* 42.7, 53.2, 76.1, 78.8, 82.7, 95.5, 117.6, 118.4, 123.1, 127.8, 128.7 (2C), 128.8, 128.9 (2C), 132.1, 134.5, 153.8, 166.6, 169.9. HRMS (ESI) *m/z*: [M + H]^+^ calcd for C_20_H_18_Cl_3_N_2_O_5_ 471.0276, found 471.0276.

Methyl (S)-2-[((E)-4-methoxybenzylidene)amino]-2-((2S*,3R*,4R*)-3-nitro-2-(trichloromethyl)chroman-4-yl)acetate (**10b**). Yield 170 mg (66%), mp 155–157 °C. IR (ATR) ν 1737 (C=O), 1553, 1310 (NO_2_). ^1^H NMR (500 MHz) *δ* 3.82 (s, 3H, MeO), 3.85 (s, 3H, MeO_2_C), 4.11 (br. s, 1H, H-4*’*), 4.48 (d, *J* = 2.3 Hz, 1H, H-2), 5.25 (d, *J* = 1.6 Hz, 1H, H-2*’*), 6.29 (br. d, *J* = 1.6 Hz, 1H, H-3*’*), 6.89 (d, *J* = 8.7 Hz, 2H, H-3,5 4-MeOC_6_H_4_), 7.03 (d, *J* = 8.0 Hz, 1H, H-8*’*), 7.09 (t, *J* = 7.5 Hz, 1H, H-6*’*), 7.20–7.27 (m, 2H, H-5*’*,7*’*), 7.54 (d, *J* = 8.7 Hz, 2H, H-2,6 4-MeOC_6_H_4_), 7.92 (s, 1H, =CH); ^13^C NMR (126 MHz) *δ* 42.7 (C-4*’*), 53.1 (MeO), 55.4 (MeO), 76.0 (C-2), 78.7 (C-3*’*), 82.6 (C-2*’*), 95.6 (CCl_3_), 114.2 (C-3,5 4-MeOC_6_H_4_), 117.5 (C-5*’*), 118.6 (C-4a*’*), 123.0 (C-6*’*), 127.5 (C-1 4-MeOC_6_H_4_), 127.8 (C-8*’*), 128.7 (C-7*’*), 130.4 (C-2,6 4-MeOC_6_H_4_), 153.8 (C-8a*’*), 162.8 (C-4 4-MeOC_6_H_4_), 165.7 (C=N), 170.2 (C=O). HRMS (ESI) *m/z*: [M + H]^+^ calcd for C_21_H_20_Cl_3_N_2_O_6_ 501.0381, found 501.0372.

*Methyl (S)-2-[((E)-benzo[d]*[1,3]dioxol-5-ylmethylene)amino]-2-((2S*,3R*,4R*)-3-nitro-2-(trichloromethyl)chroman-4-yl)acetate (**10c**). Yield 146 mg (55%), mp 189–191 °C. IR (ATR) ν 1732 (C=O), 1552, 1339 (NO_2_). ^1^H NMR (500 MHz) *δ* 3.85 (s, 3H, MeO_2_C), 4.11 (br. d, *J* = 2.4 Hz, 1H, H-4*’*), 4.48 (d, *J* = 2.4 Hz, 1H, H-2), 5.21 (d, *J* = 1.8 Hz, 1H, H-2*’*), 6.00 (s, 2H, OCH_2_O), 6.26 (dd, *J* = 1.8, 1.0 Hz, 1H, H-3*’*), 6.78 (d, *J* = 8.0 Hz, 1H, H-7 benzo[*d*][1,3]dioxol-5-yl), 7.01 (dd, *J* = 8.0, 1.5 Hz, 1H, H-6 benzo[*d*][1,3]dioxol-5-yl), 7.05 (dd, *J* = 8.3, 1.1 Hz, 1H, H-8*’*), 7.09 (dd, *J* = 7.6, 1.1 Hz, 1H, H-6*’*), 7.20 (d, *J* = 1.5 Hz, 1H, H-4 benzo[*d*][1,3]dioxol-5-yl), 7.21–7.27 (m, 2H, H-5*’*,7*’*), 7.87 (s, 1H, =CH); ^13^C NMR (126 MHz) *δ* 42.6, 53.1, 75.9, 78.7, 82.6, 95.5, 101.7, 106.5, 108.2, 117.6, 118.5, 123.1, 125.9, 127.8, 128.8, 129.3, 148.5, 151.1, 153.8, 165.5, 170.1. HRMS (ESI) *m/z*: [M + H]^+^ calcd for C_21_H_18_Cl_3_N_2_O_7_ 515.0174, found 515.0181.

Methyl (S)-2-((2S*,3R*,4R*)-6-chloro-3-nitro-2-(trichloromethyl)chroman-4-yl)-2-[((E)-4-methoxybenzylidene)amino]acetate (**10d**). Yield 161 mg (60%), mp 127–129 °C. IR (ATR) ν 1743 (C=O), 1556, 1337 (NO_2_). ^1^H NMR (500 MHz) *δ* 3.83 (s, 3H, MeO), 3.85 (s, 3H, MeO_2_C), 4.08 (br. s, 1H, H-4*’*), 4.44 (d, *J* = 1.8 Hz, 1H, H-2), 5.29 (d, *J* = 1.3 Hz, 1H, H-2*’*), 6.26 (br. s, 1H, H-3*’*), 6.90 (d, *J* = 8.6 Hz, 2H, H-3,5 4-MeOC_6_H_4_), 6.98 (d, *J* = 8.8 Hz, 1H, H-8*’*), 7.19 (dd, *J* = 8.8, 2.1 Hz, 1H, H-7*’*), 7.59 (d, *J* = 2.1 Hz, 1H, H-5*’*), 7.59 (d, *J* = 8.6 Hz, 2H, H-2,6 4-MeOC_6_H_4_), 7.99 (s, 1H, =CH); ^13^C NMR (126 MHz) *δ* 42.5, 53.2, 55.5, 75.7, 78.3, 82.8, 95.3, 114.3 (2C), 119.0, 120.4, 127.4, 127.5, 127.9, 129.0, 130.5 (2C), 152.4, 162.9, 166.1, 169.8. HRMS (ESI) *m/z*: [M + H]^+^ calcd for C_21_H_19_Cl_4_N_2_O_6_ 534.9992, found 534.9993.

Methyl (S)-2-((2S*,3R*,4R*)-6-bromo-3-nitro-2-(trichloromethyl)chroman-4-yl)-2-[((E)-4-methoxybenzylidene)amino]acetate (**10e**). Yield 194 mg (67%), mp 158–160 °C. IR (ATR) ν 1743 (C=O), 1557, 1337 (NO_2_). ^1^H NMR (500 MHz) *δ* 3.83 (s, 3H, MeO), 3.85 (s, 3H, MeO_2_C), 4.08 (s, 1H, H-4*’*), 4.44 (s, 1H, H-2), 5.30 (s, 1H, H-2*’*), 6.26 (s, 1H, H-3*’*), 6.90 (d, *J* = 8.3 Hz, 2H, H-3,5 4-MeOC_6_H_4_), 6.93 (d, *J* = 8.8 Hz, 1H, H-8*’*), 7.32 (d, *J* = 8.8 Hz, 1H, H-7*’*), 7.39 (s, 1H, H-5*’*), 7.59 (d, *J* = 8.3 Hz, 2H, H-2,6 4-MeOC_6_H_4_), 7.99 (s, 1H, =CH); ^13^C NMR (126 MHz) *δ* 42.4, 53.2, 55.5, 75.7, 78.3, 82.7, 95.3, 114.3 (2C), 115.2, 119.4, 120.9, 127.4, 130.50 (2C), 130.53, 131.8, 152.9, 162.9, 166.1, 169.8. HRMS (ESI) *m/z*: [M + H]^+^ calcd for C_21_H_19_BrCl_3_N_2_O_6_ 578.9487, found 578.9486.

Methyl (S)-2-((2S*,3R*,4R*)-6,8-dibromo-3-nitro-2-(trichloromethyl)chroman-4-yl)-2-[((E)-4-methoxybenzylidene)amino]acetate (**10f**). Yield 165 mg (50%), mp 112–115 °C. IR (ATR) ν 1735 (C=O), 1561, 1341 (NO_2_). ^1^H NMR (500 MHz) *δ* 3.81 (s, 3H, MeO), 3.84 (s, 3H, MeO_2_C), 4.11 (br. s, 1H, H-4*’*), 4.43 (d, *J* = 1.8 Hz, 1H, H-2), 5.35 (d, *J* = 1.6 Hz, 1H, H-2*’*), 6.25 (br. s, *J* = 1.5 Hz, 1H, H-3*’*), 6.92 (d, *J* = 8.6 Hz, 2H, H-3,5 4-MeOC_6_H_4_), 7.35 (d, *J* = 1.6 Hz, 1H, H-5*’*), 7.60 (d, *J* = 8.6 Hz, 2H, H-2,6 4-MeOC_6_H_4_), 7.62 (d, *J* = 1.6 Hz, 1H, H-7*’*), 8.03 (s, 1H, =CH); ^13^C NMR (126 MHz) *δ* 42.6, 53.3, 55.5, 75.5, 78.3, 83.2, 94.8, 114.4 (2C), 115.0, 122.9, 127.3, 128.1, 129.7, 130.6 (2C), 134.9, 149.8, 163.0, 166.5, 169.6. HRMS (ESI) *m/z*: [M + H]^+^ calcd for C_21_H_18_Br_2_Cl_3_N_2_O_6_ 656.8592, found 656.8590.

Methyl (S)-2-((2S*,3R*,4R*)-6-methoxy-3-nitro-2-(trichloromethyl)chroman-4-yl)-2-[((E)-4-methoxybenzylidene)amino]acetate (**10g**). Yield 106 mg (40%), mp 168–170 °C. IR (ATR) ν 1746 (C=O), 1562, 1326 (NO_2_). ^1^H NMR (500 MHz) *δ* 3.80 (s, 3H, MeO), 3.83 (s, 3H, MeO), 3.85 (s, 3H, MeO_2_C), 4.08 (dd, *J* = 2.3, 1.7 Hz, 1H, H-4*’*), 4.46 (d, *J* = 2.3 Hz, 1H, H-2), 5.18 (d, *J* = 1.7 Hz, 1H, H-2*’*), 6.25 (dd, *J* = 1.7, 1.0 Hz, 1H, H-3*’*), 6.89 (d, *J* = 8.8 Hz, 2H, H-3,5 4-MeOC_6_H_4_), 6.75 (d, *J* = 2.9 Hz, 1H, H-5*’*), 6.79 (dd, *J* = 8.9, 2.9 Hz, 1H, H-7*’*), 6.90 (d, *J* = 8.9 Hz, 1H, H-8*’*), 7.59 (d, *J* = 8.8 Hz, 2H, H-2,6 4-MeOC_6_H_4_), 7.95 (s, 1H, =CH); ^13^C NMR (126 MHz) *δ* 42.9, 53.1, 55.4, 75.8, 75.9, 78.6, 82.9, 95.6, 112.3, 114.2 (2C), 114.6, 118.3, 119.2, 127.5, 130.4 (2C), 147.9, 155.2, 162.8, 165.6, 170.2. HRMS (ESI) *m/z*: [M + H]^+^ calcd for C_22_H_22_Cl_3_N_2_O_7_ 531.0487, found 531.9486.

### 3.5. Biology

#### 3.5.1. Cell Cultures

The human cervical carcinoma (HeLa) cell line was purchased from the Bank of Cell Cultures of the Institute of Cytology of the Russian Academy of Sciences, St. Petersburg, Russia. The normal human dermal fibroblasts (HDF) cell line was obtained from the Institute of Medical Cell Technologies, Ekaterinburg, Russia.

#### 3.5.2. Assessment of In Vitro Cytotoxic Activity

The cells were seeded in 96-well microplates at a seeding density of 2 × 10^5^ cells per mL and cultured for 24 h in DMEM medium with glutamine (1%) in the presence of 10% fetal bovine serum and gentamicin (50 mg/L) at 37 °C in a humidified atmosphere containing 5% CO_2_. Then the tested compounds were added to the wells in various concentrations (10^−7^ M, 10^−6^ M, 10^−5^ M, 10^−4^ M). Cells with compounds were incubated for 72 h, after which cell viability was assessed using the standard MTT test [43] based on the reduction of the yellow tetrazole salt by living cell mitochondrial dehydrogenases to formazan crystals, soluble in DMSO. Experiments were performed in triplicates with negative control (culture medium), positive control (camptothecin, 3 mM) and solvent control (DMSO). The results of the MTT test were evaluated by comparing the optical density of the formazan solution measured on a flatbed scanner Tecan Infinite M200 PRO (Tecan Austria GmbH, Austria) at a wavelength of 570 nm in the experimental and control wells and control wells and calculating the cytotoxicity index (IC). The cytotoxicity index was determined for each concentration of the studied substances by AAT Bioquest-calculator: https://www.aatbio.com/tools/ic50-calculator (accessed on 15 November 2022). The parameters of the arithmetic mean value and the standard error were calculated. The differences in the average values according to the Mann-Whitney U test with *p* < 0.05 were considered reliable. For the statistical analysis, Microsoft Excel 2019 (Microsoft corp., Redmond, DC, USA) and Statistika 13.3 (Tibco, Palo Alto, CA, USA) were used.

## Figures and Tables

**Table 1:** Condition optimization for the reaction of **4a** with **5b** ^a^.

Entry	Catalyst	Solvent	Total Yield ^b^, %	Ratio ^c^ of *endo:endo’*
1	AgOAc	PhMe	60	2.1:1
2	AgOAc	MeCN	91	2.1:1
3	AgOAc	THF	92	2.1:1
**4**	**AgOAc**	**DCM**	**93**	**2.1:1**
5	CuI	PhMe	40	2.1:1
6	CuI	MeCN	70	2.1:1
7	CuI	THF	70	2.1:1
8	CuI	DCM	78	2.1:1

^a^ Conditions: a mixture of **4a** (63 mg, 0.25 mmol) and **5b** (58 mg, 0.28 mmol) was stirred at room temperature in 1 mL of the corresponding solvent for 5 h in the presence of Et_3_N (2.9 mg, 0.025 mmol) and the corresponding catalyst (0.025 mmol). ^b^ Isolated yield. ^c^ Determined by ^1^H NMR spectroscopy of the reaction mixtures.

**Table 2:** Scope of the synthesis of 4-Ph-substituted chromeno[3,4-*c*]pyrrolidines **8 ^a^**.

Chromene 4	R^1^	Imine 5	Ar	Product	Total yield ^b^, %	Yield ^b^ *endo*-8, %	Yield ^b^ *endo’*-8, %	Ratio ^c^ of *endo:endo’*
**a**	H	**a**	Ph	**8a**	87	56	23	2.0:1
**a**	H	**b**	4-MeOC_6_H_4_	**8b**	93	63	21	2.1:1
**a**	H	**c**	3,4-(MeO)_2_C_6_H_3_	**8c**	90	54	27	2.2:1
**a**	H	**d**	2,3,4-(MeO)_3_C_6_H_2_	**8d**	87	55	− ^d^	2.3:1
**a**	H	**e**	benzo[*d*][1,3]dioxol-5-yl	**8e**	85	56	20	2.3:1
**b**	Br	**b**	4-MeOC_6_H_4_	**8f**	92	61	21	2.2:1
**c**	MeO	**b**	4-MeOC_6_H_4_	**8g**	88	57	22	2.2:1

^a^ Conditions: a mixture of the appropriate chromene **4** (0.5 mmol), azomethine **5** (0.55 mmol), Et_3_N (5 mg, 0.05 mmol) and AgOAc (5.8 mg, 0.05 mmol) was stirred at room temperature in DCM (2 mL) for 5 h. ^b^ Isolated yield. ^c^ Determined by ^1^H NMR spectroscopy of the reaction mixtures. ^d^ This isomer was not isolated from the reaction mixture.

**Table 3:** Scope of the synthesis of 4-CF_3_-substituted chromeno[3,4-*c*]pyrrolidines **9 ^a^**.

Chromene 4	R^1^	R^2^	Imine 5	Ar	Product	Yield ^b^, %
**d**	H	H	**a**	Ph	*endo*-**9a**	90
**d**	H	H	**b**	4-MeOC_6_H_4_	*endo*-**9b**	92
**d**	H	H	**c**	3,4-(MeO)_2_C_6_H_3_	*endo*-**9c**	92
**d**	H	H	**d**	2,3,4-(MeO)_3_C_6_H_2_	*endo*-**9d**	85
**d**	H	H	**e**	benzo[*d*][1,3]dioxol-5-yl	*endo*-**9e**	87
**e**	Cl	H	**b**	4-MeOC_6_H_4_	*endo*-**9f**	90
**f**	Br	H	**b**	4-MeOC_6_H_4_	*endo*-**9g**	94
**g**	Br	Br	**b**	4-MeOC_6_H_4_	*endo*-**9h**	93
**h**	MeO	H	**b**	4-MeOC_6_H_4_	*endo*-**9i**	89
**i**	H	EtO	**b**	4-MeOC_6_H_4_	*endo*-**9j**	87

^a^ Conditions: a mixture of the appropriate chromene **4** (0.5 mmol), azomethine **5** (0.55 mmol), Et_3_N (5 mg, 0.05 mmol) and AgOAc (5.8 mg, 0.05 mmol) was stirred at room temperature in DCM (2 mL) for 5 h. ^b^ Isolated yield.

**Table 4:** Scope of the synthesis of 2-CCl_3_-substituted chromanes **10** ^a^.

Chromene 4	R^1^	Imine 5	Ar	Product	Yield ^b^, %
**j**	H	**a**	Ph	**10a**	43
**j**	H	**b**	4-MeOC_6_H_4_	**10b**	66
**j**	H	**e**	benzo[*d*][1,3]dioxol-5-yl	**10c**	55
**k**	Cl	**b**	4-MeOC_6_H_4_	**10d**	60
**l**	Br	**b**	4-MeOC_6_H_4_	**10e**	67
**m**	Br	**b**	4-MeOC_6_H_4_	**10f**	50
**n**	MeO	**b**	4-MeOC_6_H_4_	**10g**	40

^a^ Conditions: a mixture of the appropriate chromene **4** (0.5 mmol), azomethine **5** (0.55 mmol), Et_3_N (5 mg, 0.05 mmol,) and AgOAc (5.8 mg, 0.05 mmol) was stirred at room temperature in DCM (2 mL) for 5 h. ^b^ Isolated yield.

**Table 5:** Cytotoxic activity (IC_50_) of compounds *endo*-**8b**,**e−g** and *endo*-**9b**,**e−j** against HeLa and HDF cell lines.

Compound	IC_50_, μM	
	HeLa	HDF
*endo*-**8b**	5300 ± 120.0	33.23 ± 1.65
*endo*-**8e**	44.98 ± 2.15	806.00 ± 41.54
*endo*-**8f**	5610 ± 37.0	745.0 ± 16.40
*endo*-**8g**	5220 ± 117.0	781.0 ± 17.50
*endo*-**9b**	0.55 ± 0.01	− ^a^
*endo*-**9e**	108 ± 15.8	185 ± 14.2
*endo*-**9f**	50.22 ± 3.40	1750.00 ± 120.0
*endo*-**9g**	3100.0 ± 84.8	225.00 ± 47.20
*endo*-**9h**	4400.0 ± 90.1	8750.0 ± 647.0
*endo*-**9i**	12.17 ± 1.37	213.00 ± 51.25
*endo*-**9j**	4.19 ± 0.05	0.74 ± 0.05
Camptothecin	1.66 ± 0.97	323.27 ± 28.93

^a^ This compound is not cytotoxic in the concentration range from 10^−7^ M to 10^−4^ M.

## Data Availability

Data is contained within the article and Supplementary Material.

## Supplementary Materials

The following supporting information can be downloaded at: https://www.mdpi.com/article/10.3390/molecules27248983/s1: X-ray diffraction experiments; NMR spectra of compounds **8**–**10** [44,45].

## Sample Availability

Samples of the compounds are not available from the authors.
