# Supplementary material for: Different Behavior of 2-Substituted 3-Nitro-2H-chromenes in the Reaction with Stabilized Azomethine Ylides Generated from α-Iminoesters
Source: Molecules. 2022 Dec 16;27(24):8983. doi: 10.3390/molecules27248983 (PMC9784934; doi:10.3390/molecules27248983)
Supplement: Supplementary file 1 [file molecules-27-08983-s001.zip › molecules-2094114-supplementary.pdf]

Supporting information for

Different behavior of 2-substituted 3-nitro-2*H*-chromenes in the reaction with stabilized azomethine ylides generated from α-iminoesters

Ivan A. Kochnev, Alexey Y. Barkov, Nikita S. Simonov, Maria V. Ulitko, Nikolay S. Zimnitskiy,

Vladislav Y. Korotaev* and Vyacheslav Y. Sosnovskikh*

Institute of Natural Sciences and Mathematics, Ural Federal University, 51 Lenina Ave.,

620000 Ekaterinburg, Russian Federation

E-mail: *korotev.vladislav@urfu.ru*, *vy.sosnovskikh@urfu.ru*

Table of Contents

1. Copies of ^{1}H and ^{13}C NMR spectra of synthesized compounds S2–S34

2. Copies of 2D NMR spectra of synthesized compounds S35–S37

3. X-ray crystallography data S38–S39

4. References S40

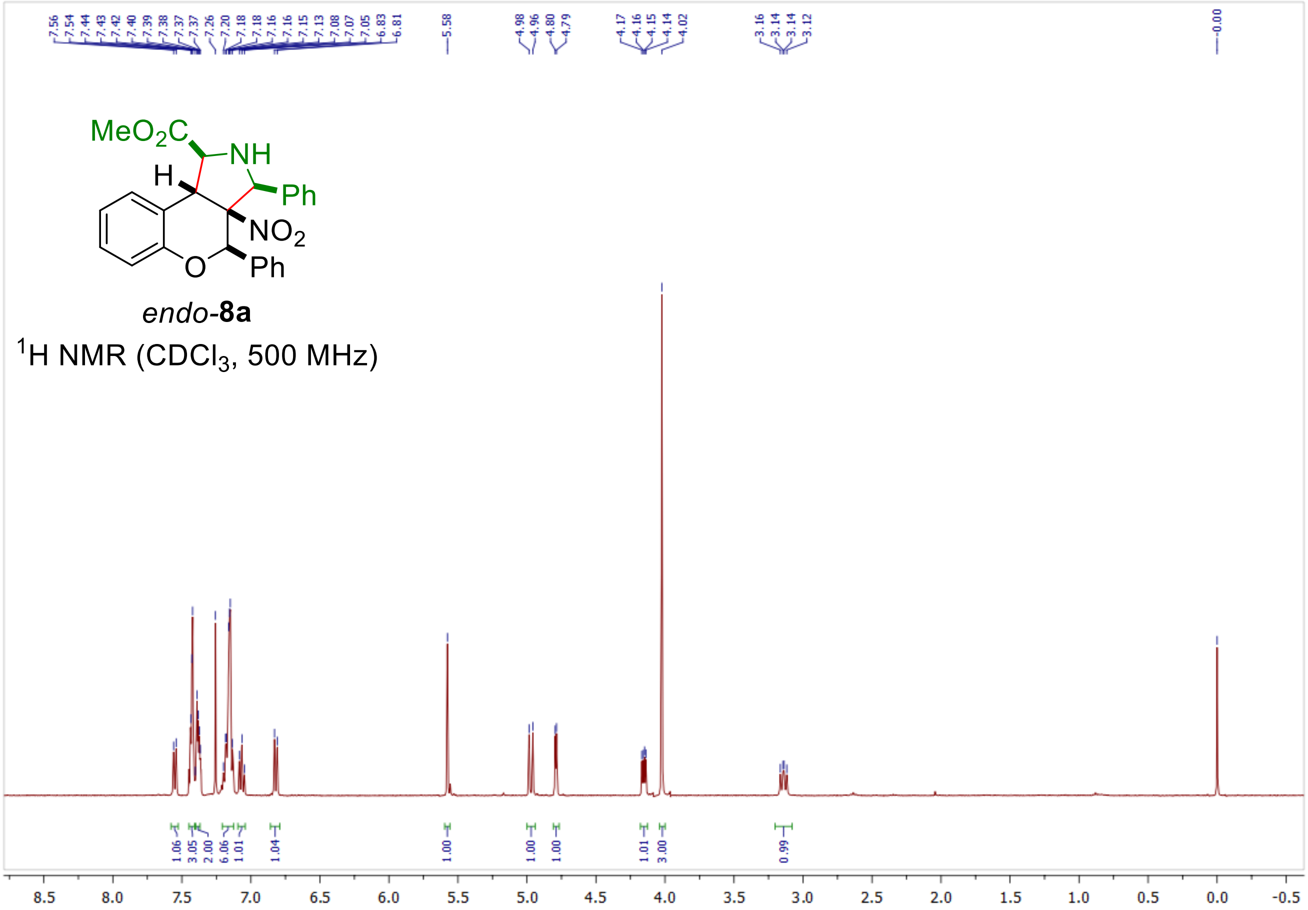
7.56 7.54 7.44 7.43 7.42 7.40 7.39 7.38 7.37 7.37 7.26 7.20 7.18 7.18 7.16 7.16 7.15 7.13 7.08 7.07 7.05 6.83 6.81
5.58
4.98 4.96 4.80 4.79
4.17 4.16 4.15 4.14 4.02
3.16 3.14 3.14 3.12
0.00
MeO2C
NH
H
Ph
NO2
O
Ph
endo-8a
1H NMR (CDCl3, 500 MHz)
1.06 3.05 2.00 6.06 1.01 1.04 1.00 1.00 1.00 1.01 3.00 0.99
8.5 8.0 7.5 7.0 6.5 6.0 5.5 5.0 4.5 4.0 3.5 3.0 2.5 2.0 1.5 1.0 0.5 0.0 -0.5

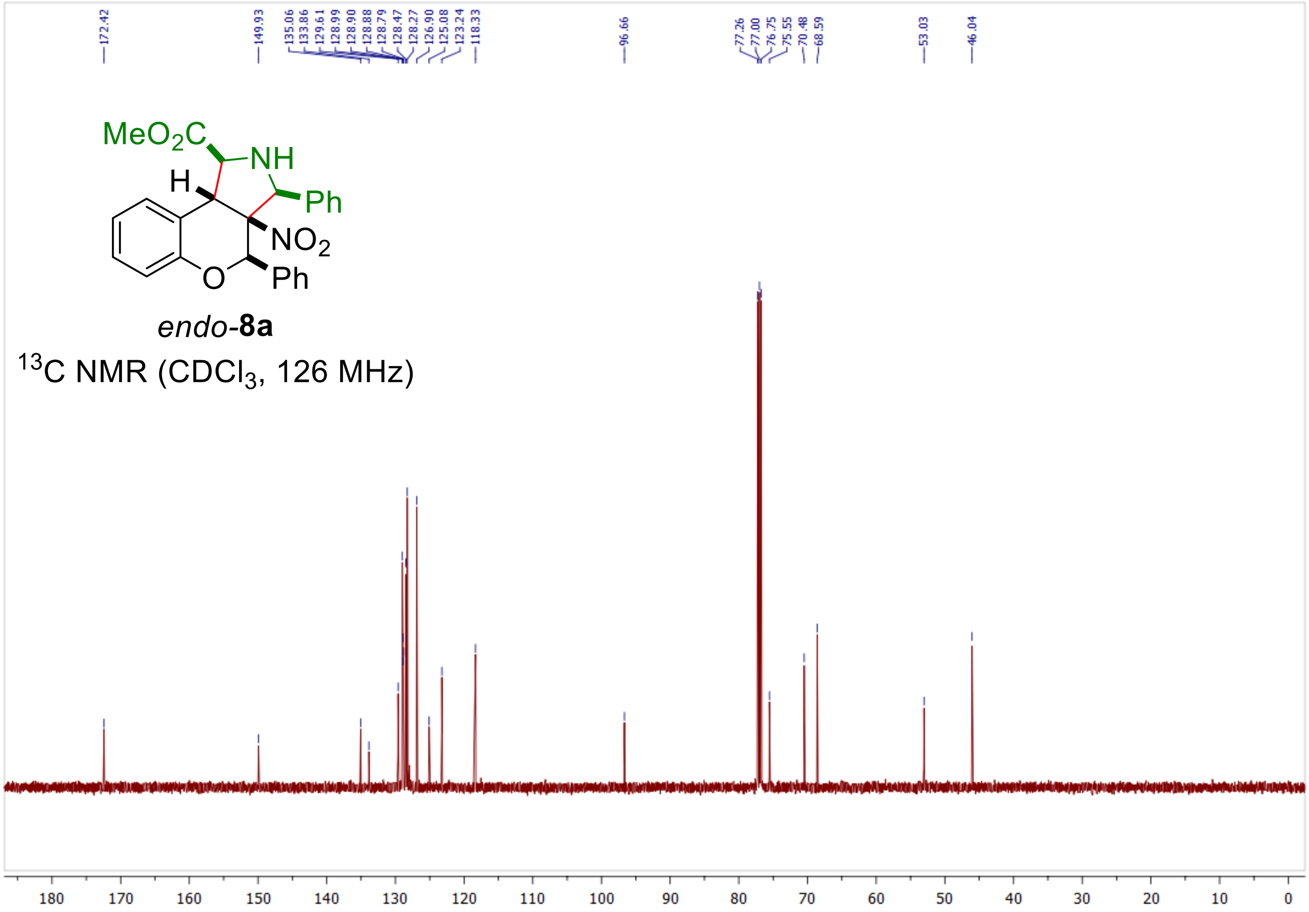
172.42
149.93
135.06 133.86 129.61 128.99 128.90 128.88 128.79 128.47 128.27 126.90 125.08 123.24 118.33
96.66
77.26 77.00 76.75 75.55 70.48 68.59
53.03
46.04
MeO2C
NH
H
Ph
NO2
O
Ph
endo-8a
13C NMR (CDCl3, 126 MHz)
180 170 160 150 140 130 120 110 100 90 80 70 60 50 40 30 20 10 0

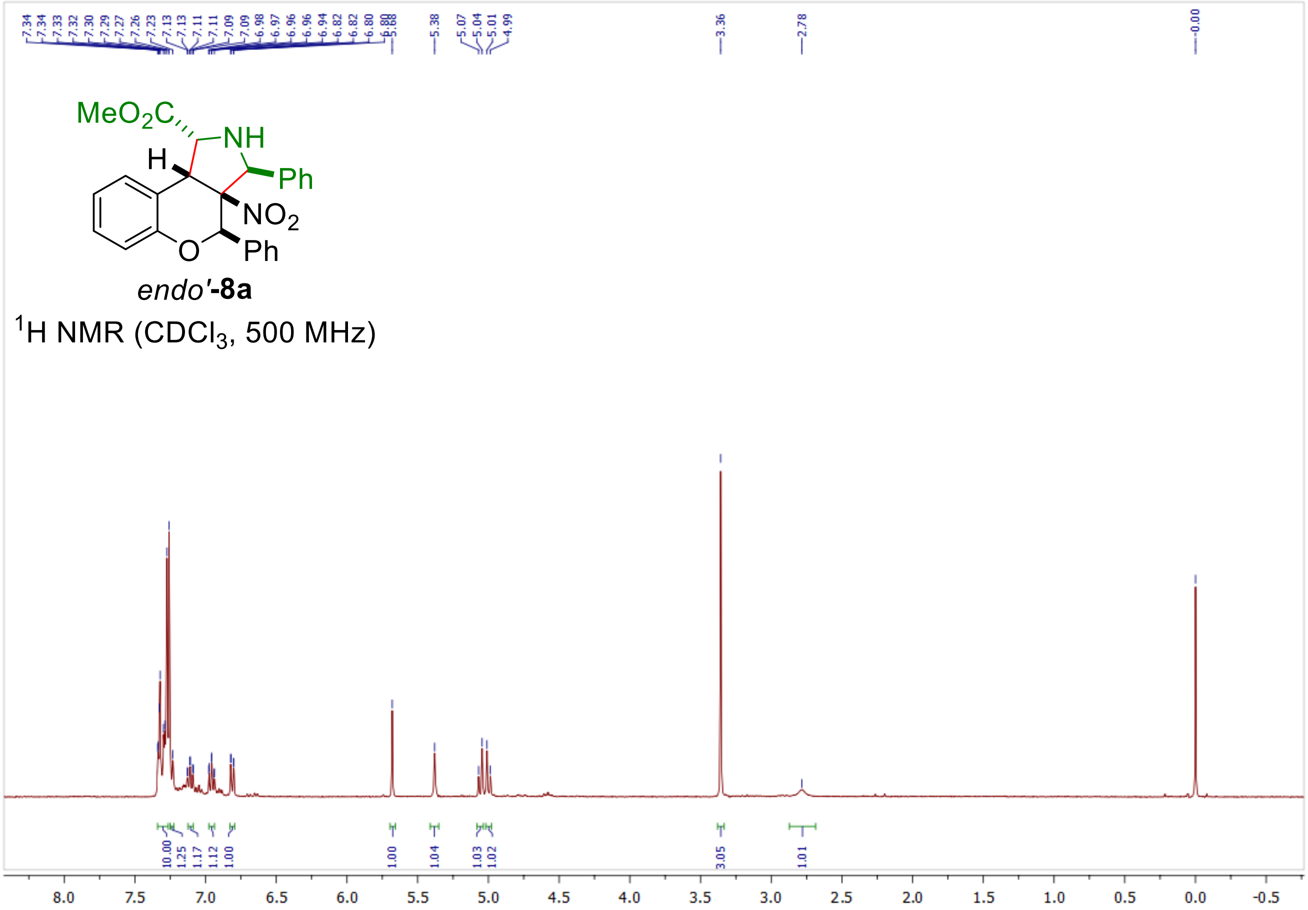
MeO2C
NH
H
Ph
NO2
O
Ph
endo'-8a
1H NMR (CDCl3, 500 MHz)
8.0
7.5
7.0
6.5
6.0
5.5
5.0
4.5
4.0
3.5
3.0
2.5
2.0
1.5
1.0
0.5
0.0
-0.5

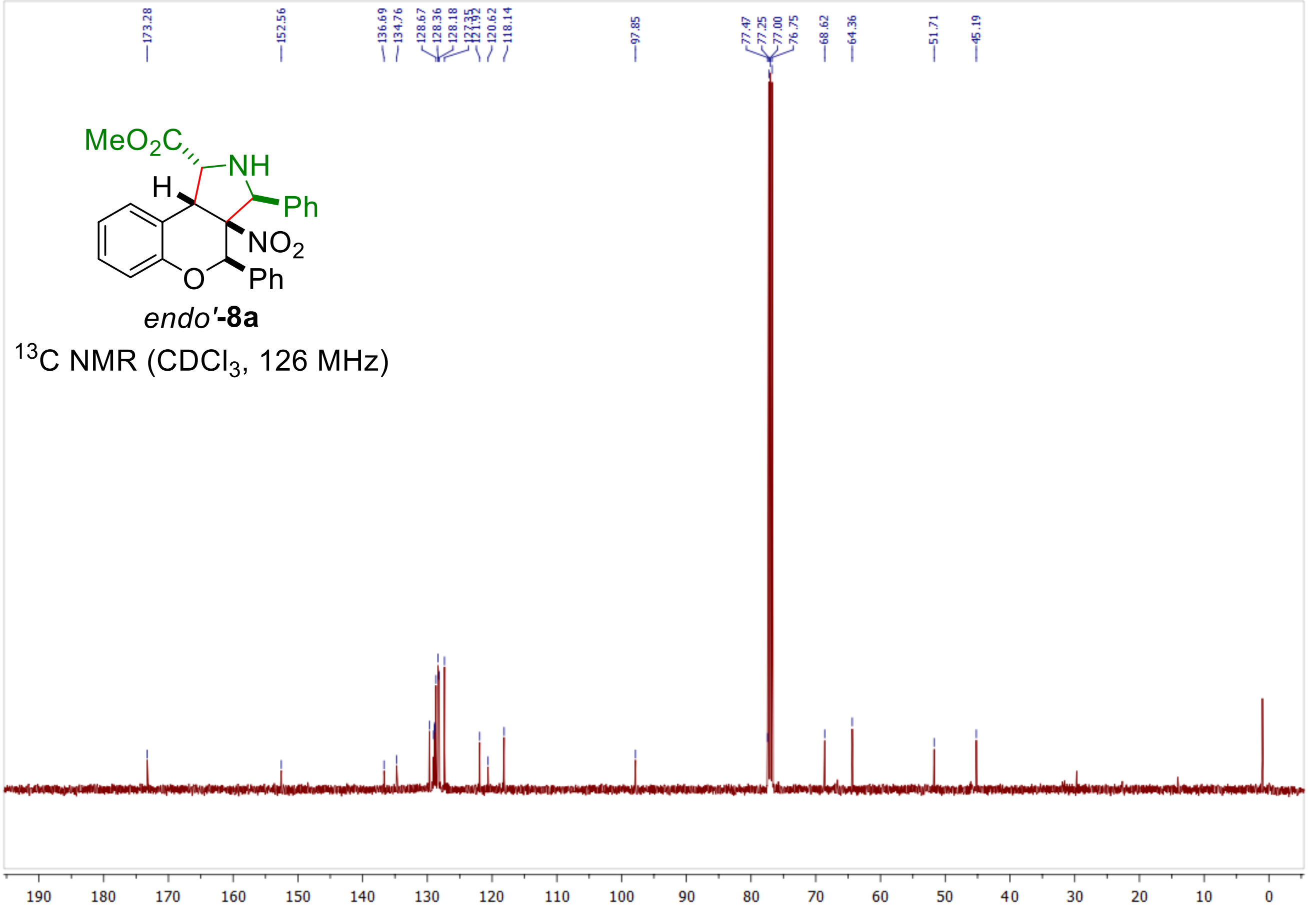
MeO2C
NH
H
Ph
NO2
O
Ph
endo'-8a
13C NMR (CDCl3, 126 MHz)
190
180
170
160
150
140
130
120
110
100
90
80
70
60
50
40
30
20
10
0

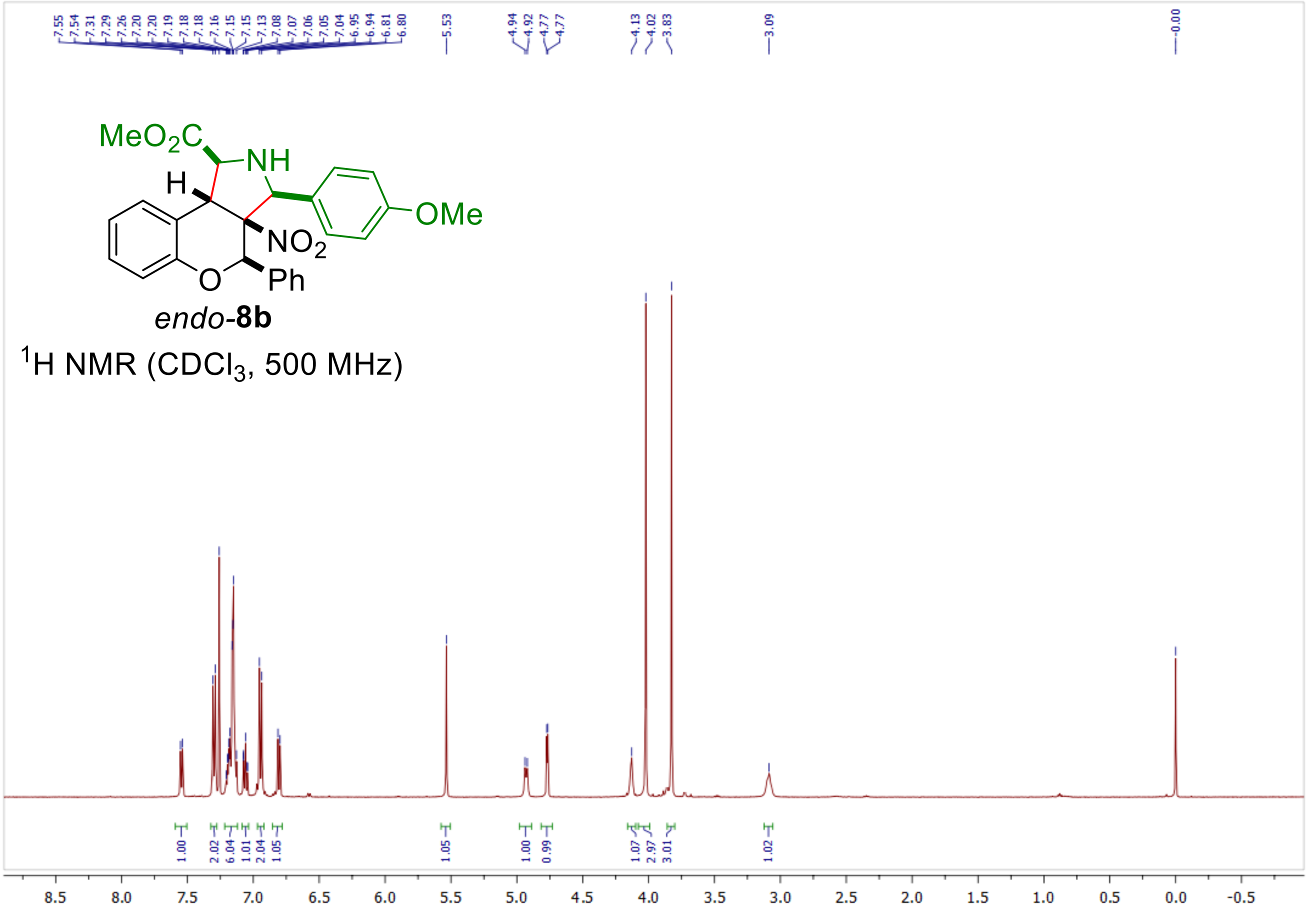
7.55
7.54
7.31
7.29
7.26
7.20
7.20
7.19
7.18
7.18
7.16
7.15
7.15
7.13
7.08
7.07
7.06
7.05
7.04
6.95
6.94
6.81
6.80
5.53
4.94
4.92
4.77
4.77
4.13
4.02
3.83
3.09
0.00
MeO2C
NH
H
OMe
NO2
O
Ph
endo-8b
1H NMR (CDCl3, 500 MHz)
1.00
2.02
6.04
1.01
2.04
1.05
1.05
1.00
0.99
1.07
2.97
3.01
1.02
8.5
8.0
7.5
7.0
6.5
6.0
5.5
5.0
4.5
4.0
3.5
3.0
2.5
2.0
1.5
1.0
0.5
0.0
-0.5

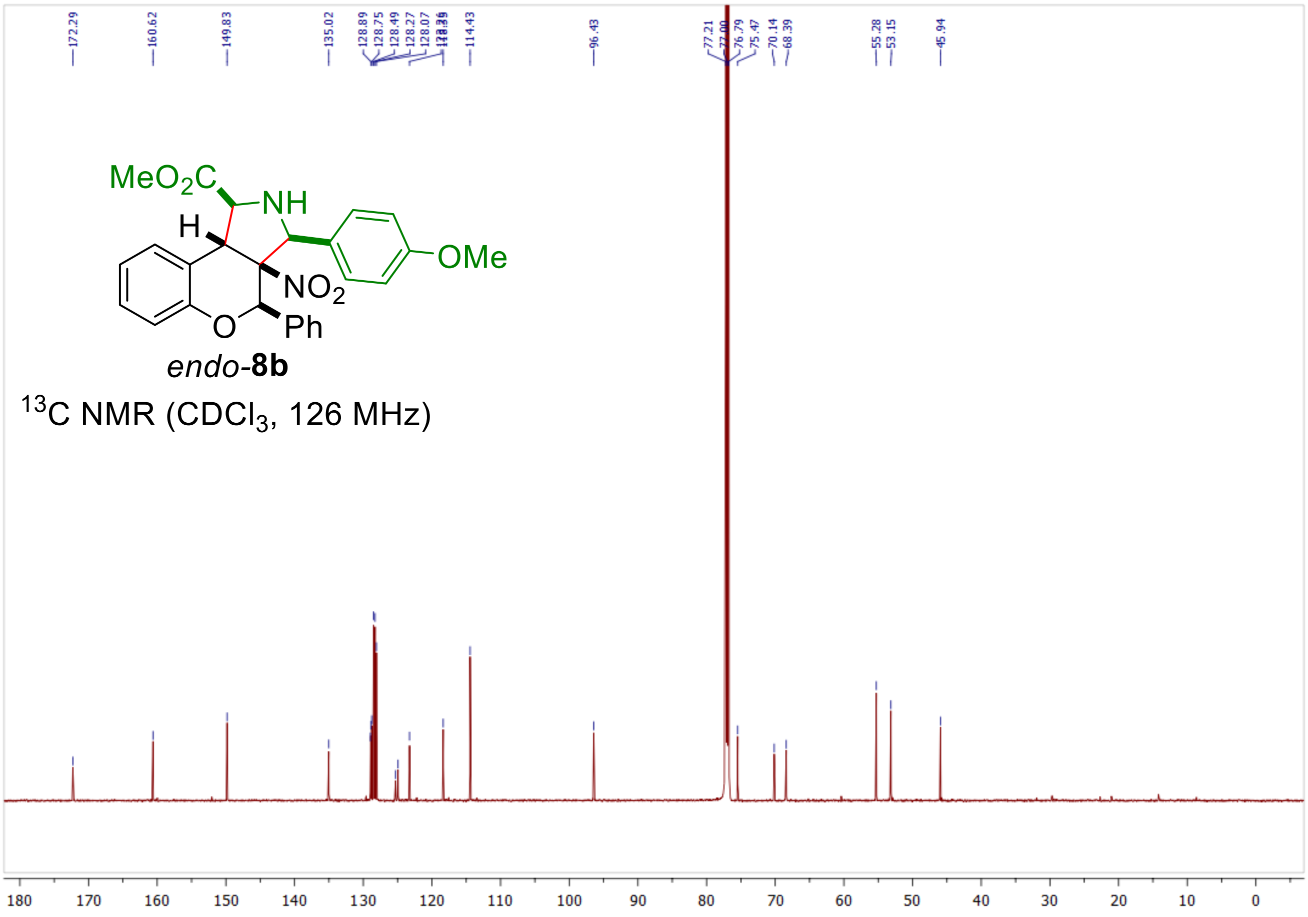
172.29
160.62
149.83
135.02
128.89
128.75
128.49
128.27
128.07
114.43
96.43
77.21
77.00
76.79
75.47
70.14
68.39
55.28
53.15
45.94
MeO2C
NH
H
OMe
NO2
O
Ph
endo-8b
13C NMR (CDCl3, 126 MHz)
180
170
160
150
140
130
120
110
100
90
80
70
60
50
40
30
20
10
0

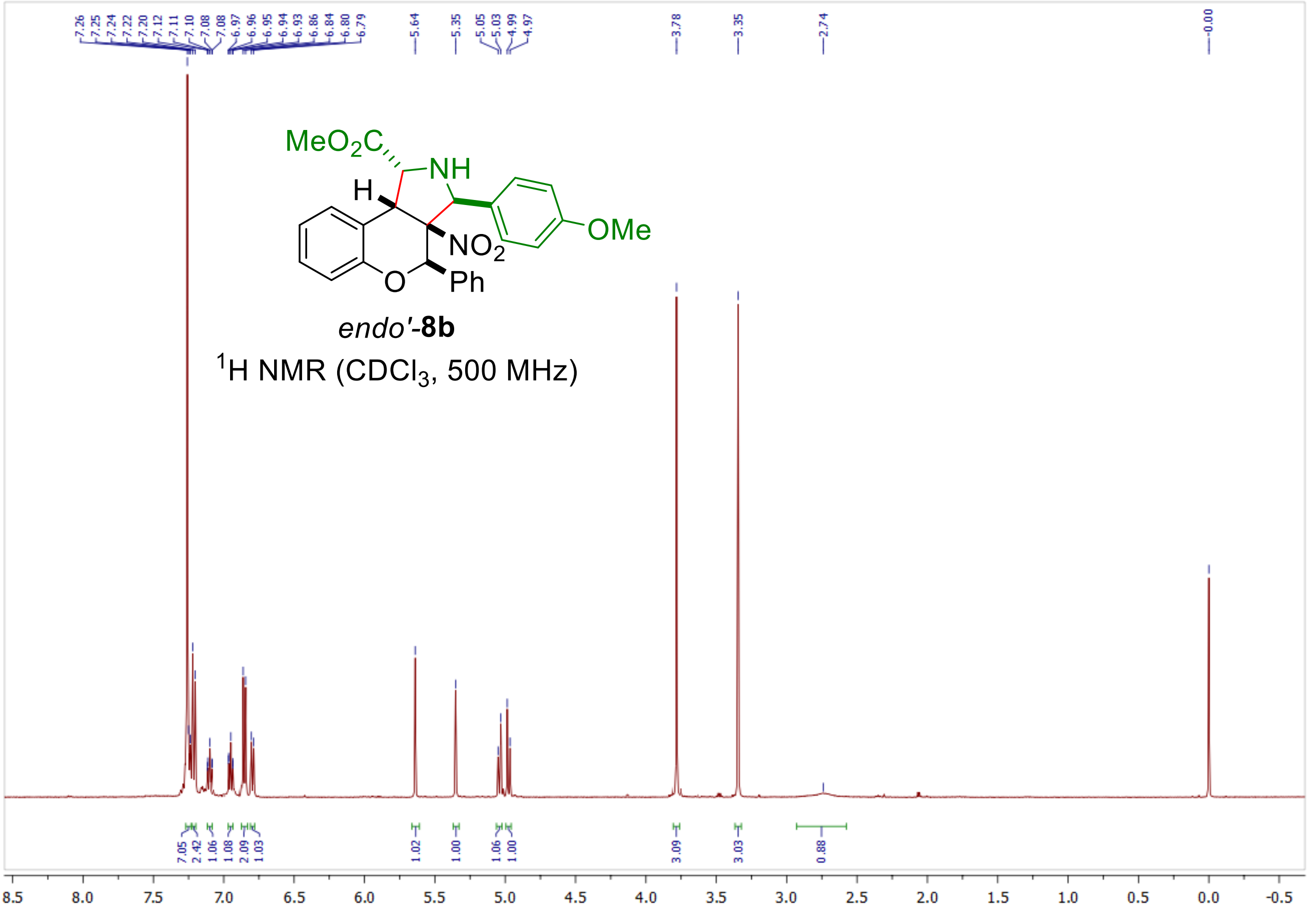
7.26 7.25 7.24 7.22 7.20 7.12 7.11 7.10 7.08 7.08 6.97 6.96 6.95 6.94 6.93 6.86 6.84 6.80 6.79
5.64
5.35
5.05 5.03 4.99 4.97
3.78
3.35
2.74
0.00
MeO2C
NH
H
NO2
OMe
O
Ph
endo'-8b
1H NMR (CDCl3, 500 MHz)
7.05 2.42 1.06 1.08 2.09 1.03 1.02 1.00 1.06 1.00 3.09 3.03 0.88
8.5 8.0 7.5 7.0 6.5 6.0 5.5 5.0 4.5 4.0 3.5 3.0 2.5 2.0 1.5 1.0 0.5 0.0 -0.5

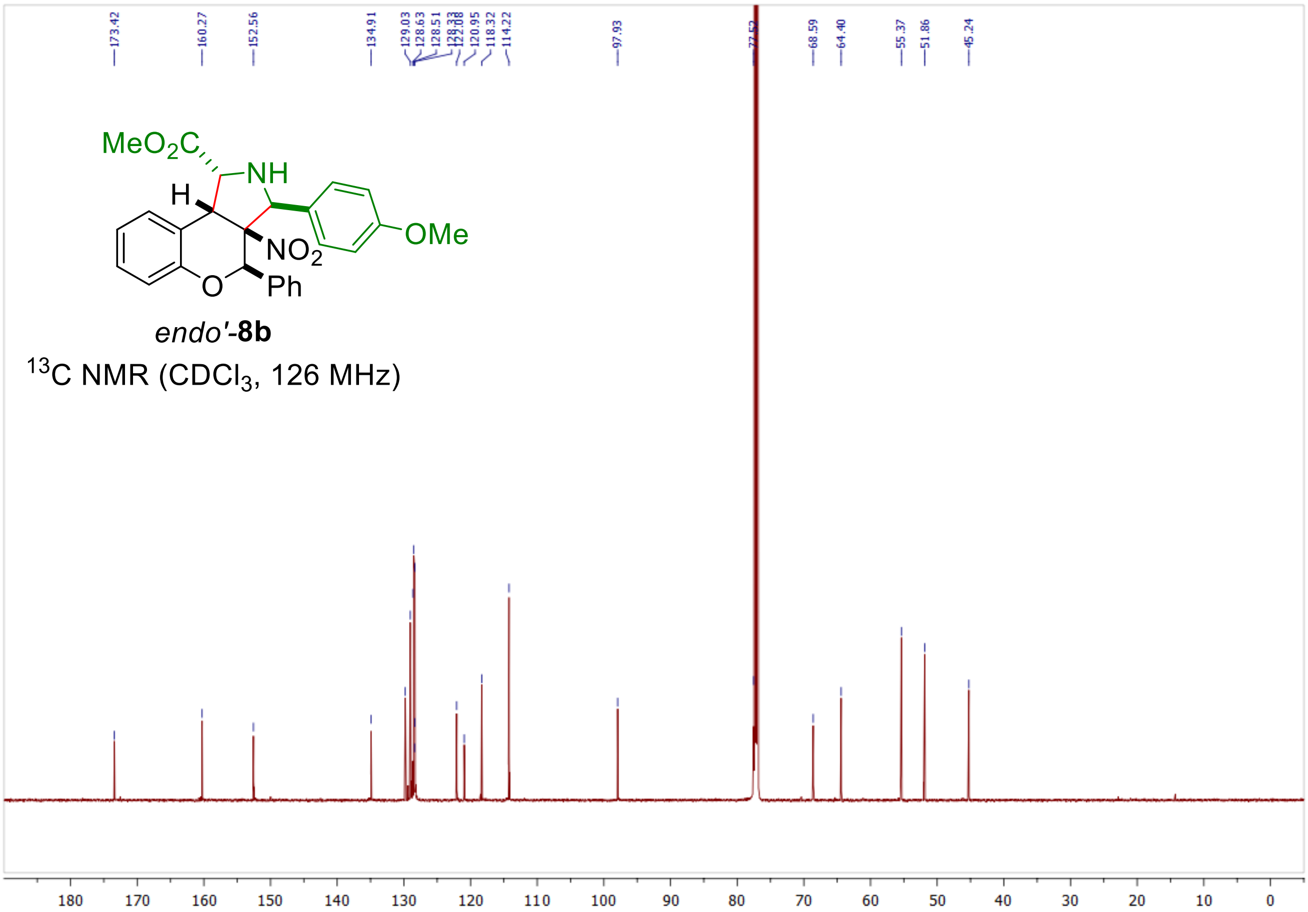
173.42
160.27
152.56
134.91
129.03 128.63 128.51 128.33 122.08 120.95 118.32 114.22
97.93
68.59
64.40
55.37
51.86
45.24
MeO2C
NH
H
NO2
OMe
O
Ph
endo'-8b
13C NMR (CDCl3, 126 MHz)
180 170 160 150 140 130 120 110 100 90 80 70 60 50 40 30 20 10 0

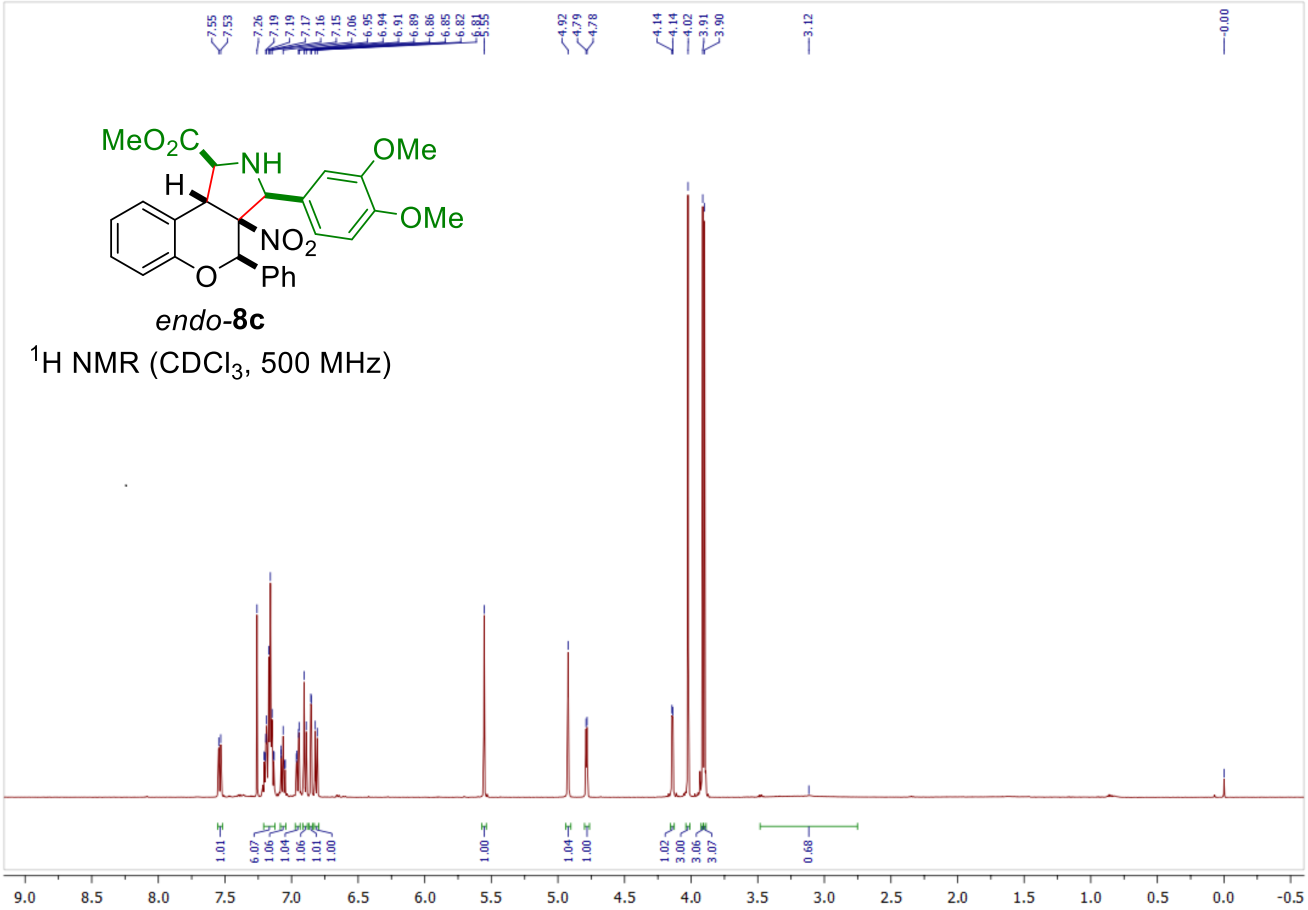
MeO2C
NH
OMe
OMe
H
NO2
O
Ph
endo-8c
1H NMR (CDCl3, 500 MHz)

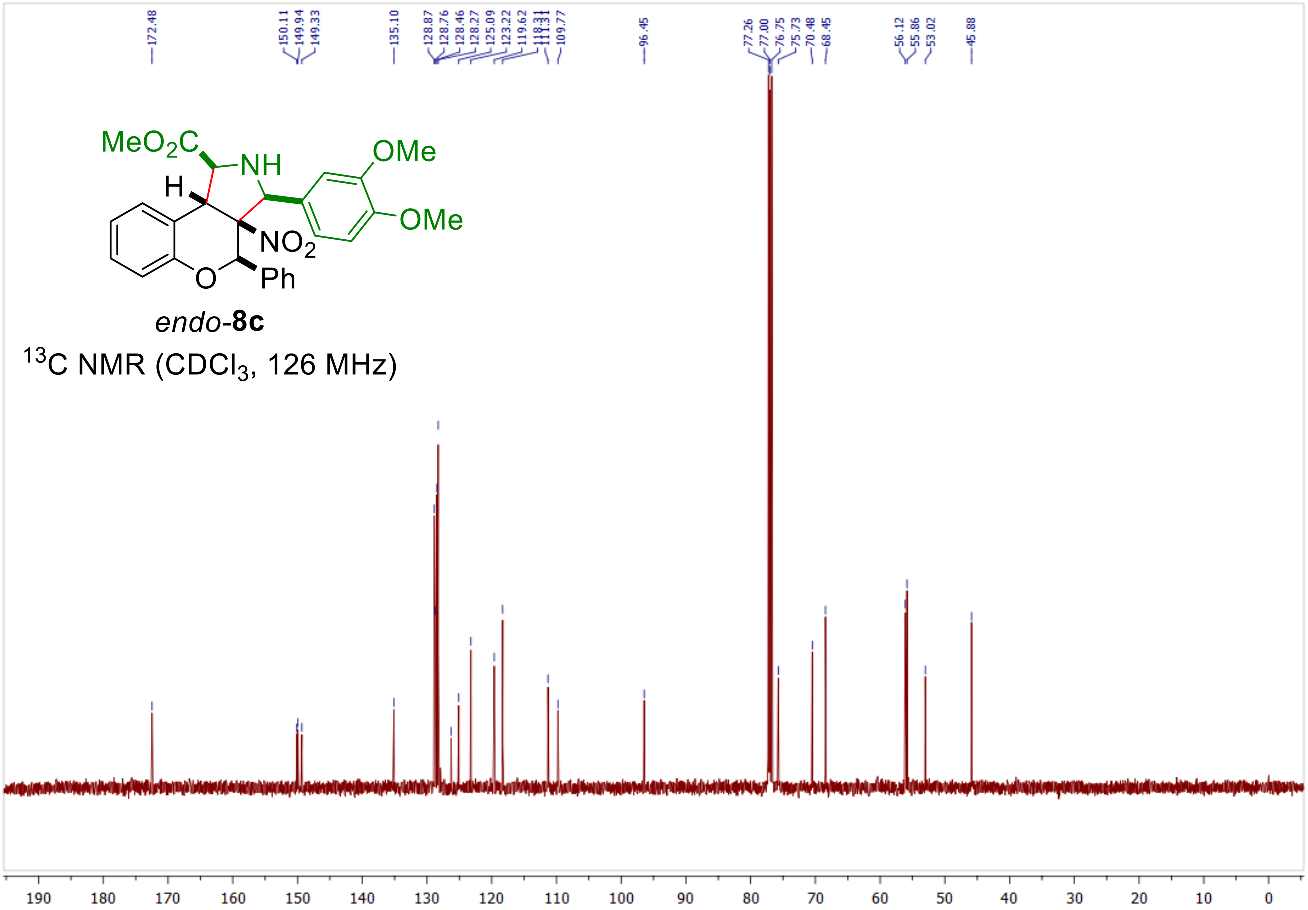
MeO2C
NH
OMe
OMe
H
NO2
O
Ph
endo-8c
13C NMR (CDCl3, 126 MHz)

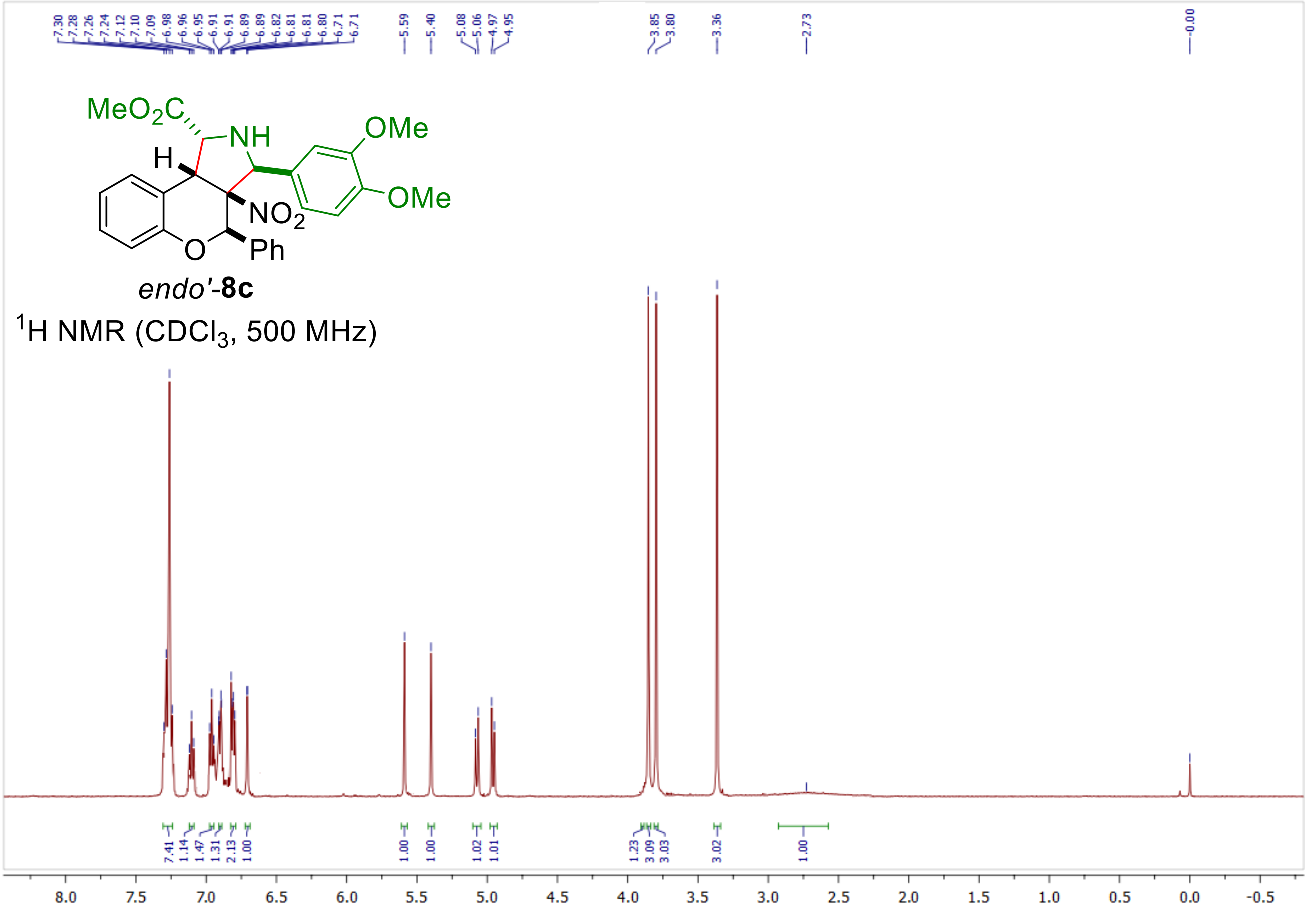
MeO2C
NH
OMe
H
OMe
NO2
O
Ph
endo'-8c
1H NMR (CDCl3, 500 MHz)

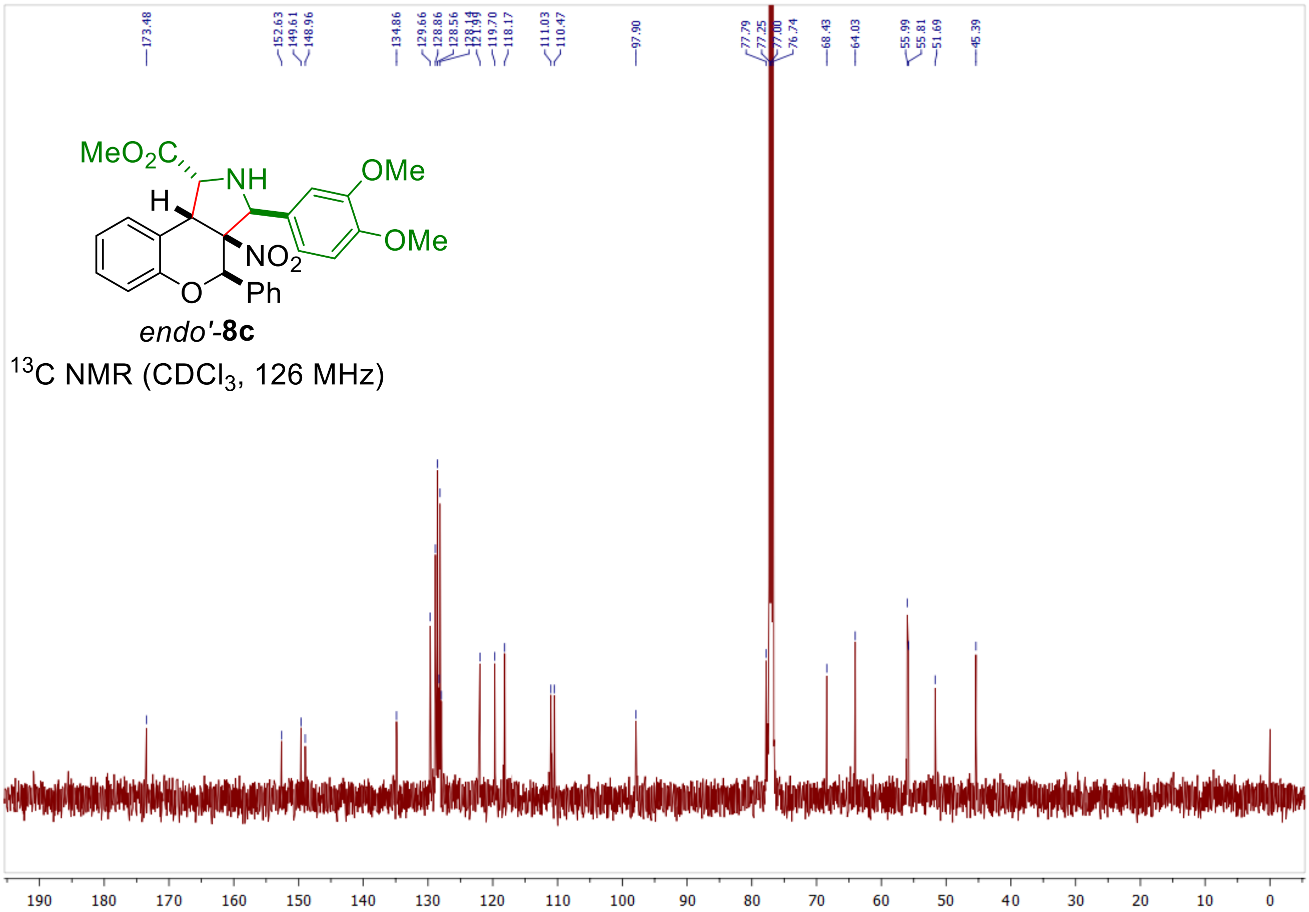
MeO2C
NH
OMe
H
OMe
NO2
O
Ph
endo'-8c
13C NMR (CDCl3, 126 MHz)

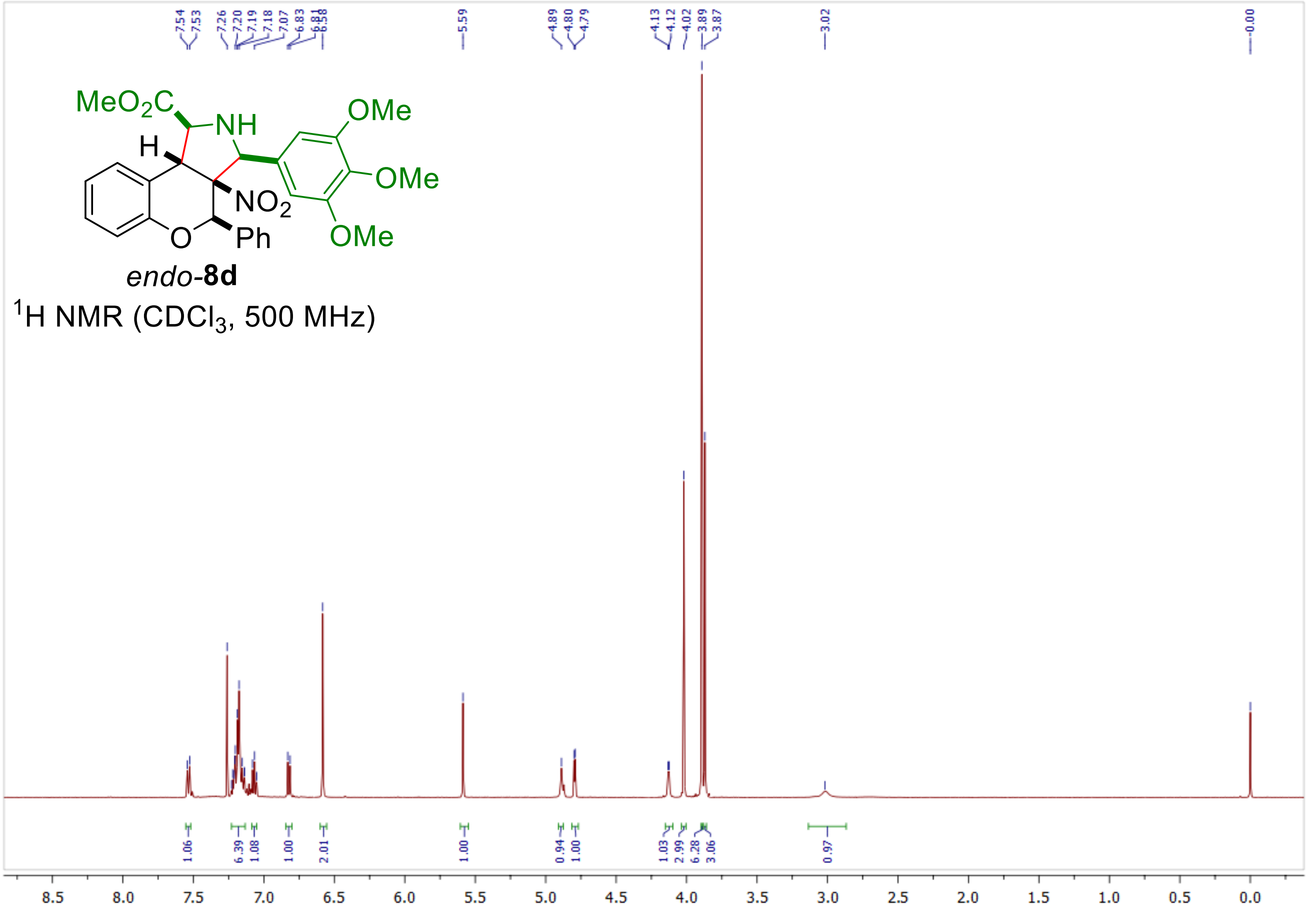
MeO2C
NH
H
OMe
OMe
NO2
O
Ph
OMe
endo-8d
1H NMR (CDCl3, 500 MHz)

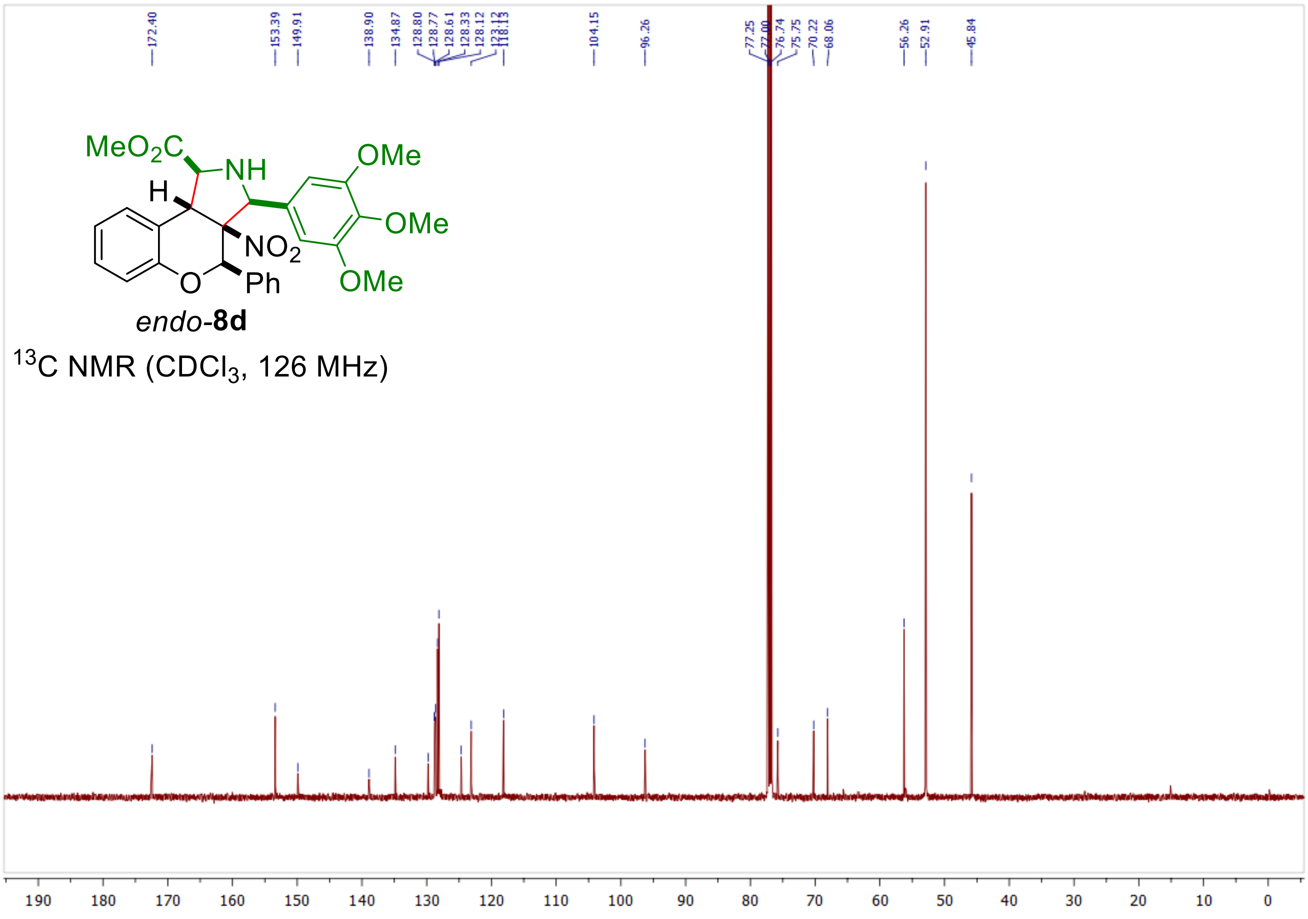
MeO2C
NH
H
OMe
OMe
NO2
O
Ph
OMe
endo-8d
13C NMR (CDCl3, 126 MHz)

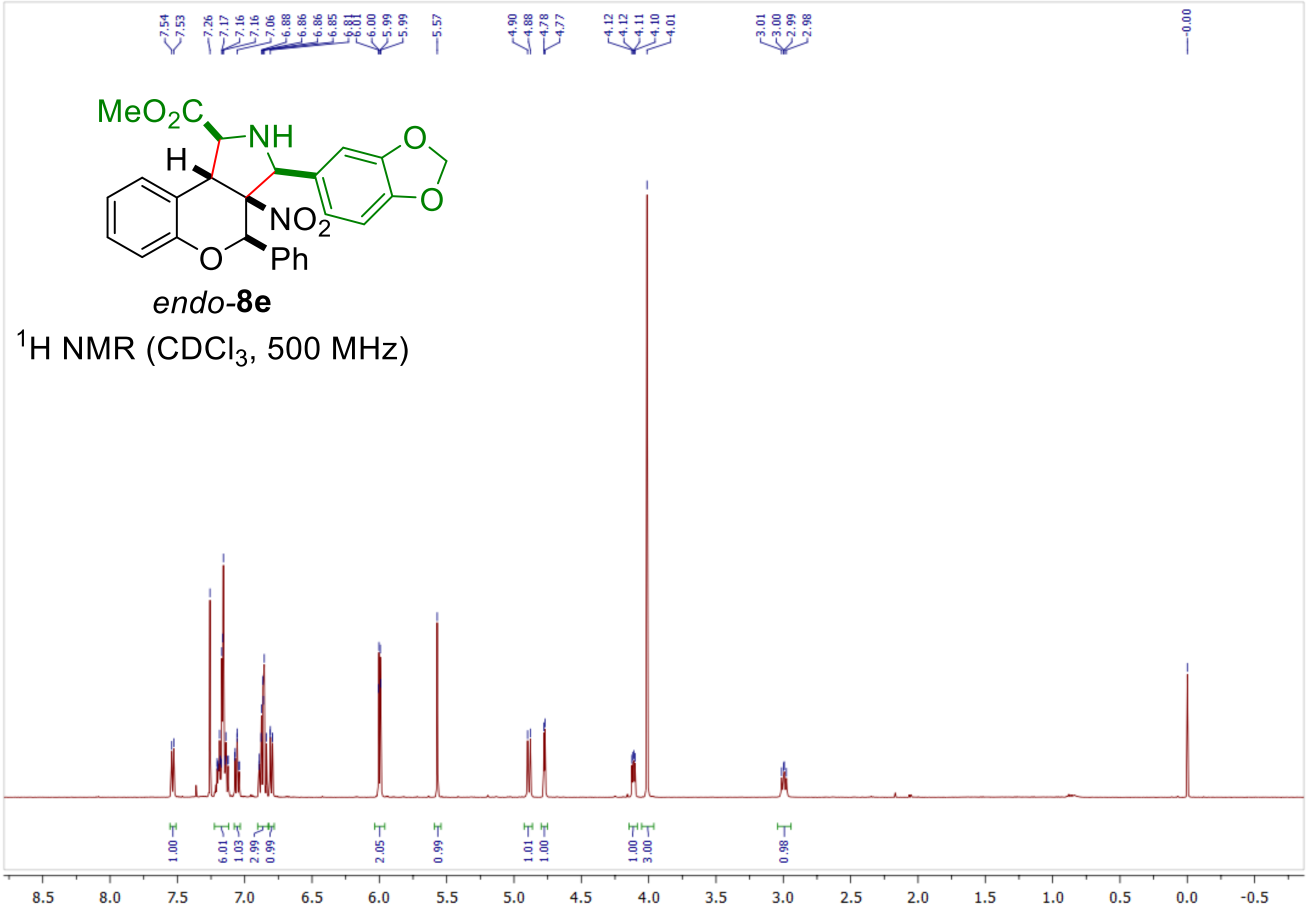
7.54 7.53 7.26 7.17 7.16 7.16 7.06 6.88 6.86 6.86 6.85 6.81 6.81 6.00 5.99 5.99 5.57 4.90 4.88 4.78 4.77 4.12 4.12 4.11 4.10 4.01 3.01 3.00 2.99 2.98 0.00
MeO2C
NH
H
NO2
O
O
O
Ph
endo-8e
1H NMR (CDCl3, 500 MHz)
1.00 6.01 1.03 2.99 0.99 0.99 2.05 0.99 1.01 1.00 1.00 3.00 0.98
8.5 8.0 7.5 7.0 6.5 6.0 5.5 5.0 4.5 4.0 3.5 3.0 2.5 2.0 1.5 1.0 0.5 0.0 -0.5

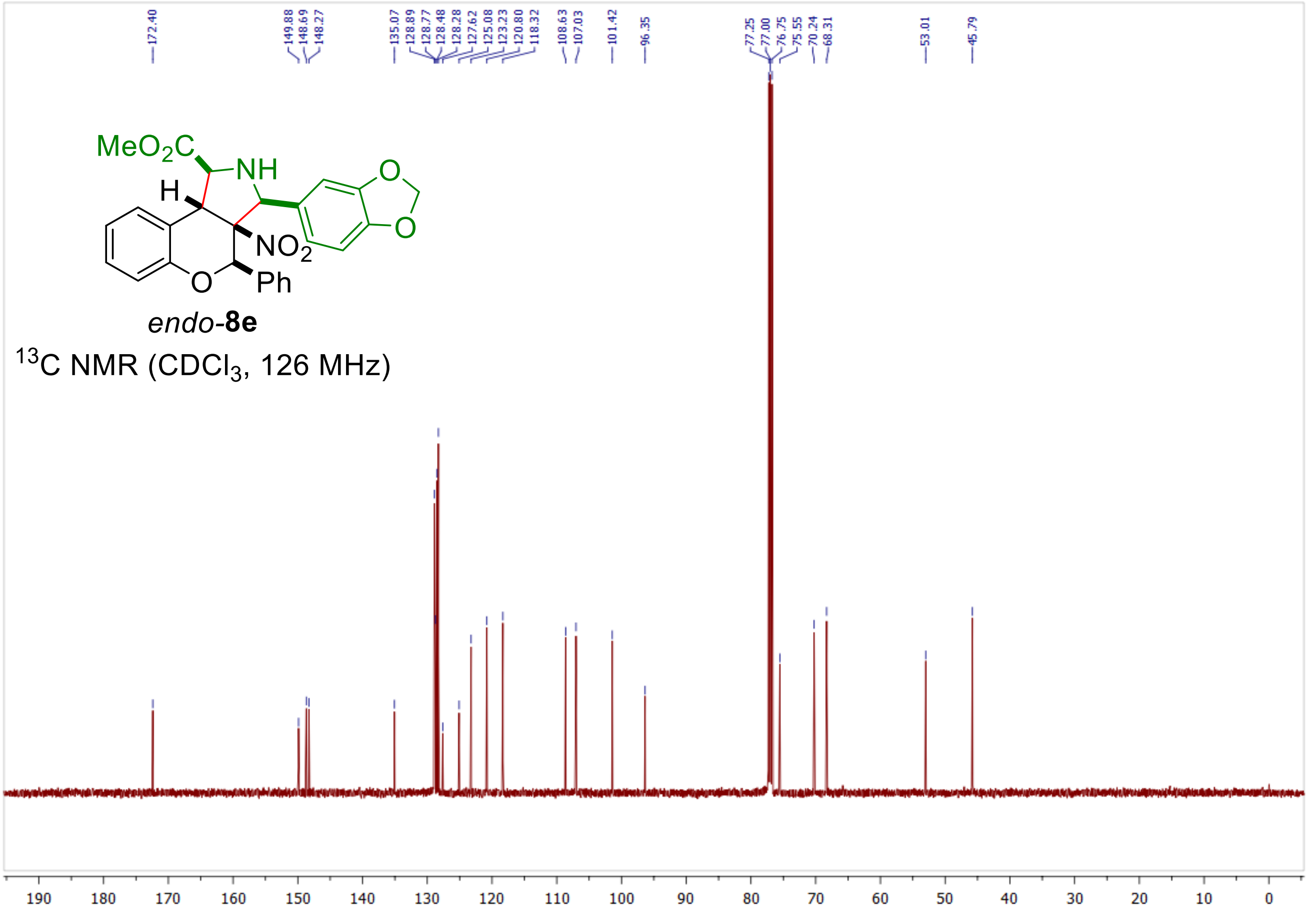
172.40 149.88 148.69 148.27 135.07 128.89 128.77 128.48 128.28 127.62 125.08 123.23 120.80 118.32 108.63 107.03 101.42 96.35 77.25 77.00 76.75 75.55 70.24 68.31 53.01 45.79
MeO2C
NH
H
NO2
O
O
O
Ph
endo-8e
13C NMR (CDCl3, 126 MHz)
190 180 170 160 150 140 130 120 110 100 90 80 70 60 50 40 30 20 10 0

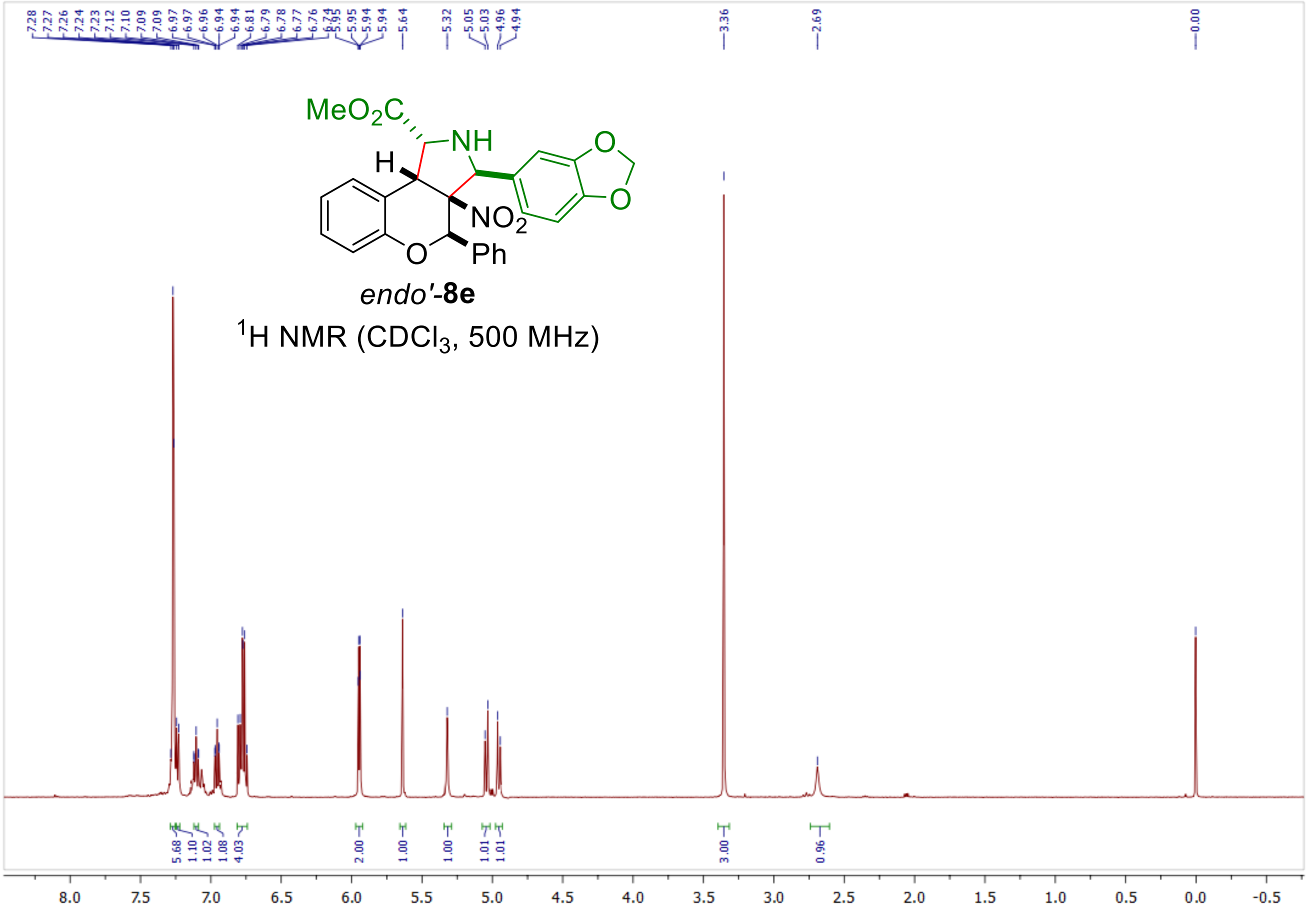
7.28 7.27 7.26 7.24 7.23 7.12 7.10 7.09 7.09 6.97 6.97 6.96 6.94 6.94 6.81 6.79 6.78 6.77 6.76 6.74 5.95 5.95 5.94 5.94 5.64 5.32 5.05 5.03 4.96 4.94 3.36 2.69 0.00
MeO2C
NH
H
NO2
O
Ph
endo'-8e
1H NMR (CDCl3, 500 MHz)
5.68 1.10 1.02 1.08 4.03 2.00 1.00 1.00 1.01 1.01 3.00 0.96
8.0 7.5 7.0 6.5 6.0 5.5 5.0 4.5 4.0 3.5 3.0 2.5 2.0 1.5 1.0 0.5 0.0 -0.5

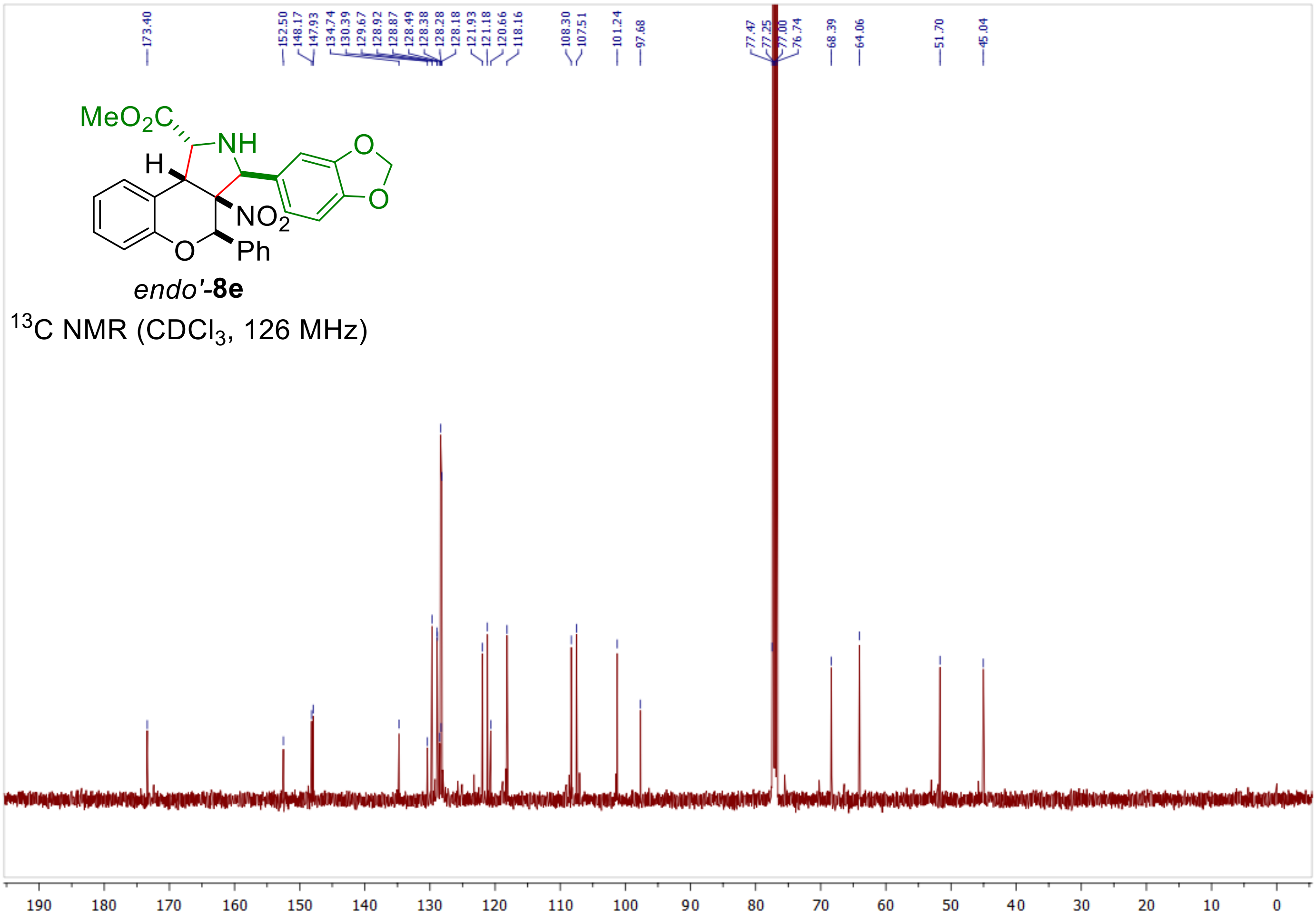
173.40 152.50 148.17 147.93 134.74 130.39 129.67 128.92 128.87 128.49 128.38 128.28 128.18 121.93 121.18 120.66 118.16 108.30 107.51 101.24 97.68 77.47 77.25 77.00 76.74 68.39 64.06 51.70 45.04
MeO2C
NH
H
NO2
O
Ph
endo'-8e
13C NMR (CDCl3, 126 MHz)
190 180 170 160 150 140 130 120 110 100 90 80 70 60 50 40 30 20 10 0

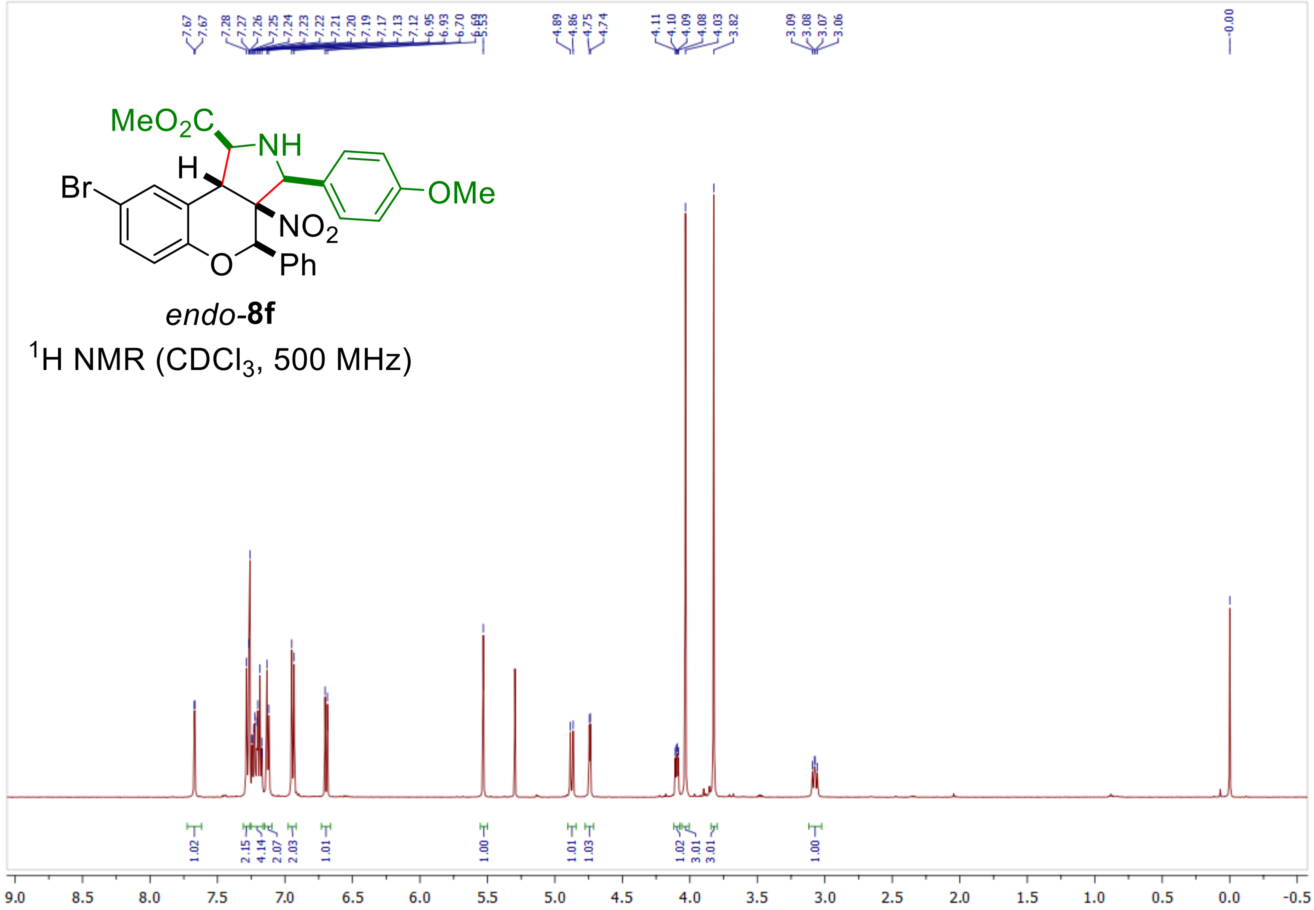
MeO2C
NH
H
Br
OMe
NO2
O
Ph
endo-8f
1H NMR (CDCl3, 500 MHz)

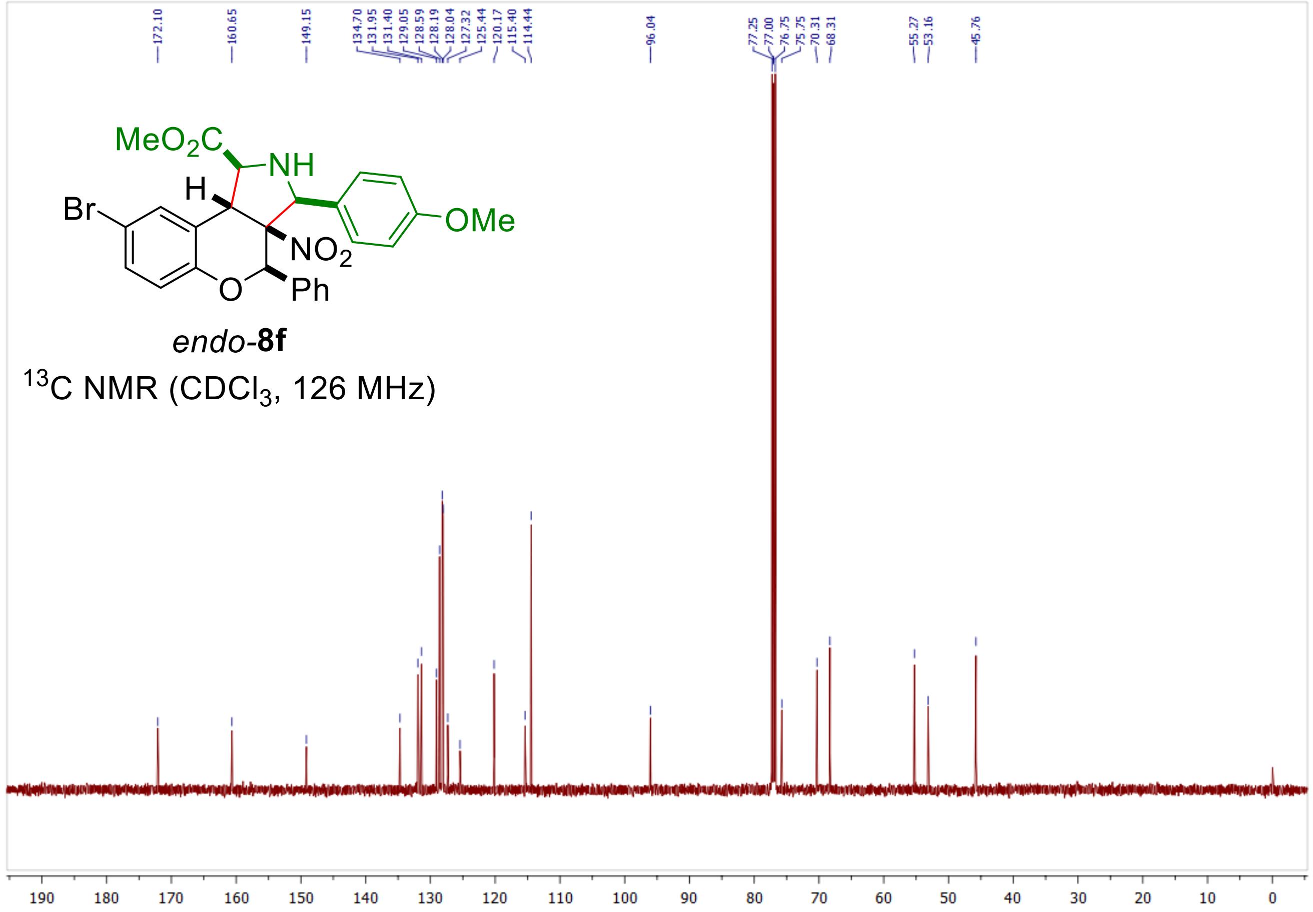
MeO2C
NH
H
Br
OMe
NO2
O
Ph
endo-8f
13C NMR (CDCl3, 126 MHz)

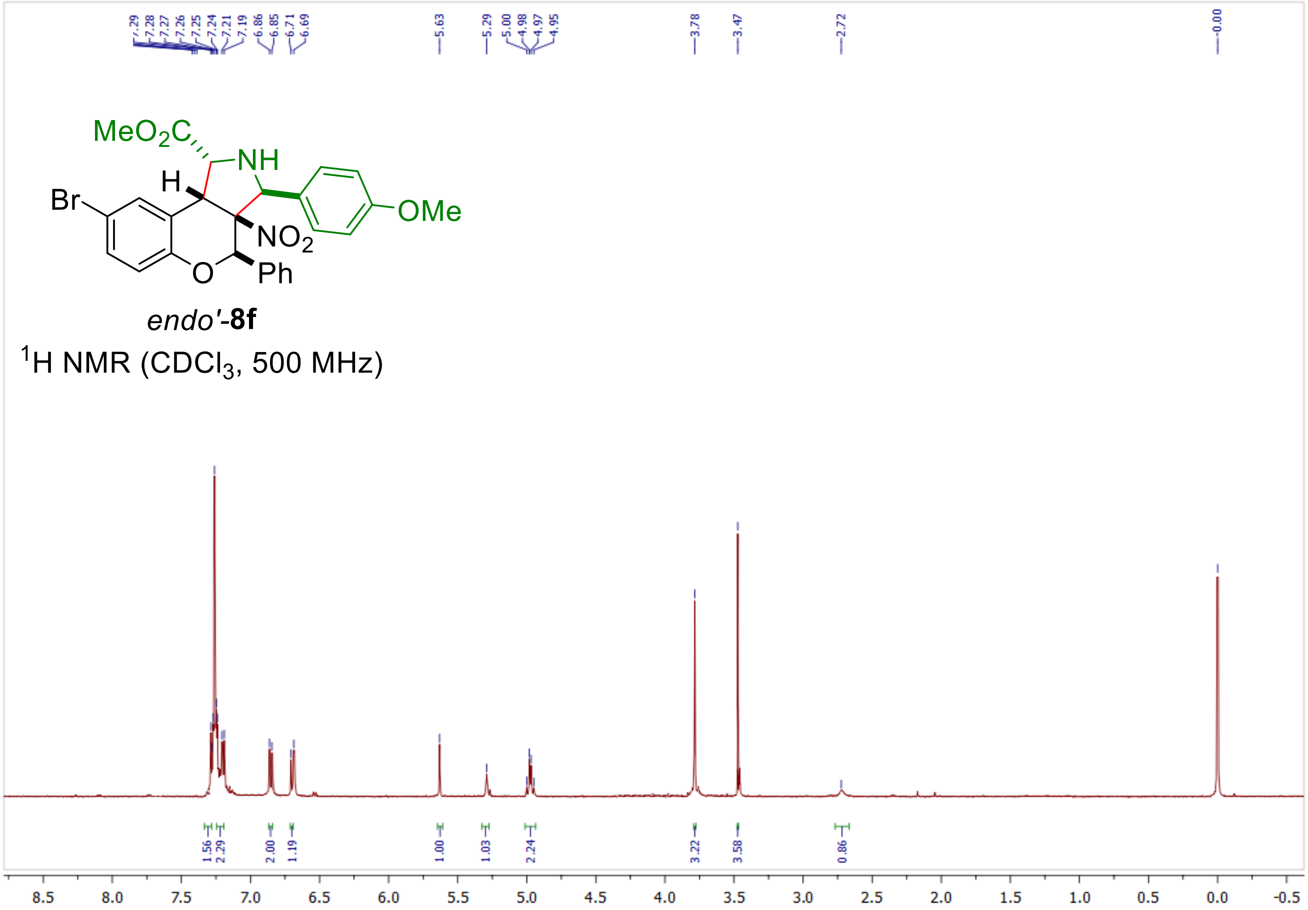
7.29
7.28
7.27
7.26
7.25
7.24
7.21
7.19
6.86
6.85
6.71
6.69
5.63
5.29
5.00
4.98
4.97
4.95
3.78
3.47
2.72
0.00
MeO2C
NH
H
Br
OMe
NO2
O
Ph
endo'-8f
1H NMR (CDCl3, 500 MHz)
1.56
2.29
2.00
1.19
1.00
1.03
2.24
3.22
3.58
0.86
8.5
8.0
7.5
7.0
6.5
6.0
5.5
5.0
4.5
4.0
3.5
3.0
2.5
2.0
1.5
1.0
0.5
0.0
-0.5

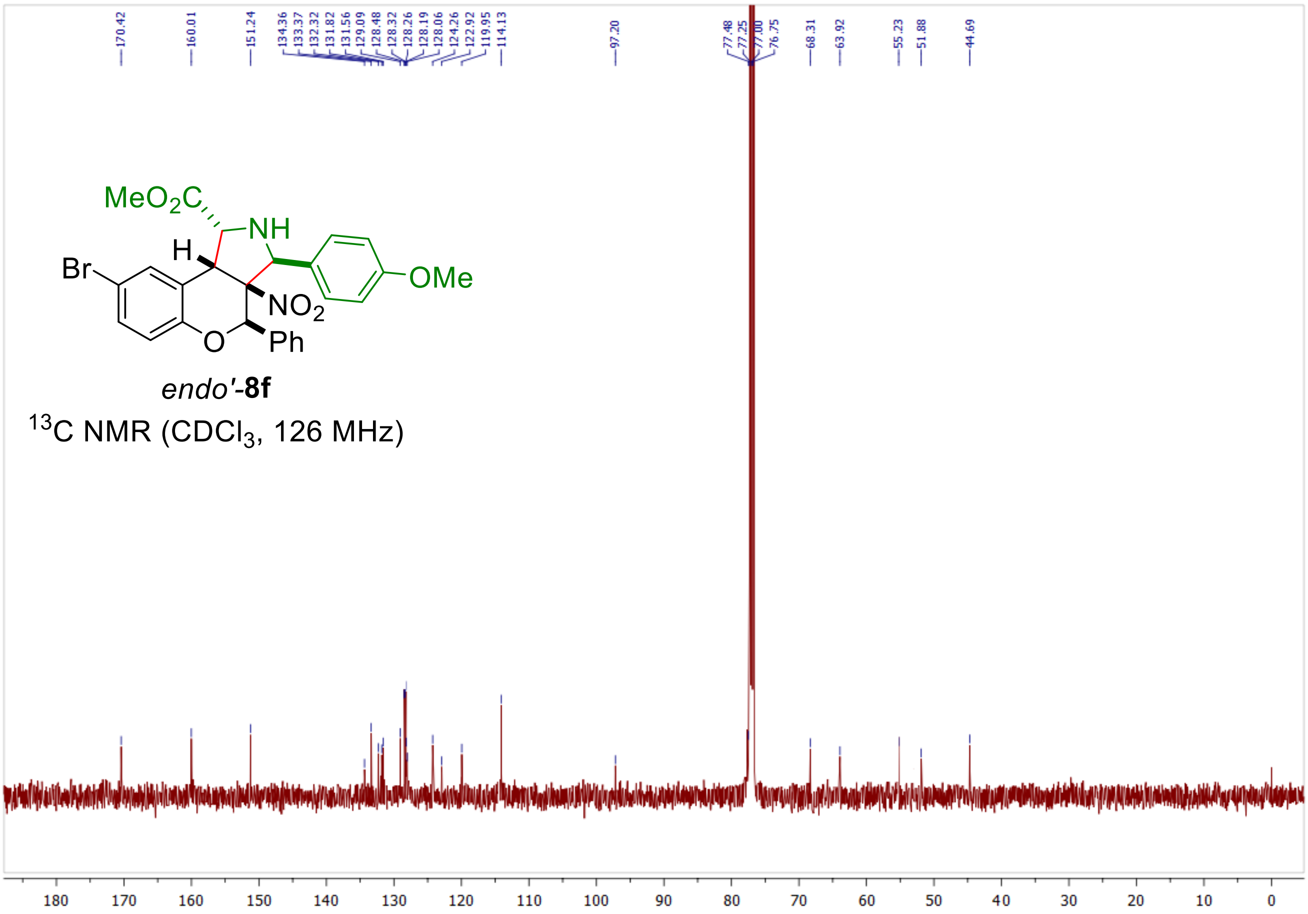
170.42
160.01
151.24
134.36
133.37
132.32
131.82
131.56
129.09
128.48
128.32
128.26
128.19
128.06
124.26
122.92
119.95
114.13
97.20
77.48
77.25
77.00
76.75
68.31
63.92
55.23
51.88
44.69
MeO2C
NH
H
Br
OMe
NO2
O
Ph
endo'-8f
13C NMR (CDCl3, 126 MHz)
180
170
160
150
140
130
120
110
100
90
80
70
60
50
40
30
20
10
0

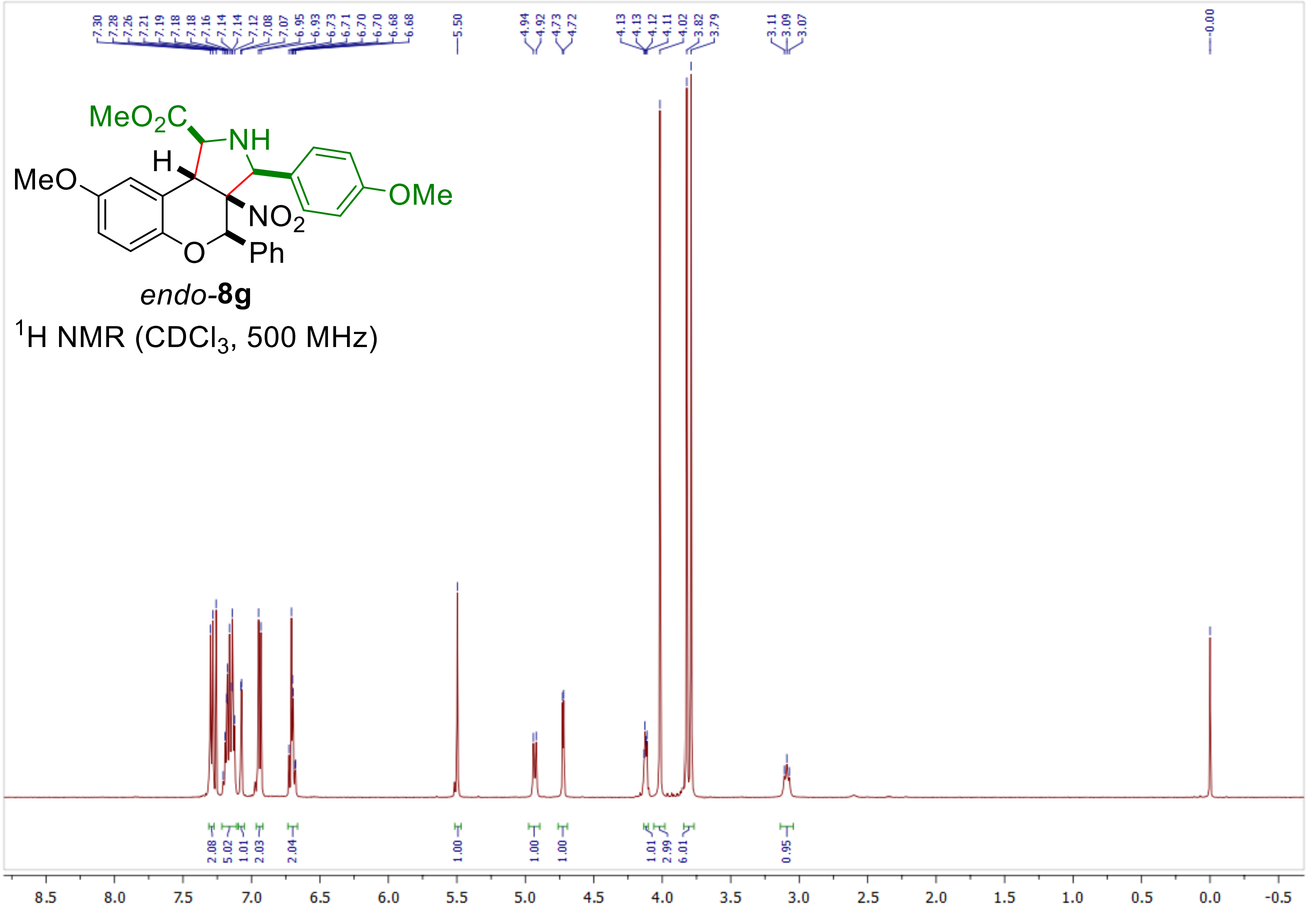
7.30 7.28 7.26 7.21 7.19 7.18 7.18 7.16 7.14 7.14 7.12 7.08 7.07 6.95 6.93 6.73 6.71 6.70 6.70 6.68 6.68
5.50
4.94 4.92 4.73 4.72
4.13 4.13 4.12 4.11 4.02 3.82 3.79
3.11 3.09 3.07
0.00
MeO2C
NH
H
MeO
OMe
NO2
O
Ph
endo-8g
1H NMR (CDCl3, 500 MHz)
2.08 5.02 1.01 2.03 2.04 1.00 1.00 1.00 1.01 2.99 6.01 0.95
8.5 8.0 7.5 7.0 6.5 6.0 5.5 5.0 4.5 4.0 3.5 3.0 2.5 2.0 1.5 1.0 0.5 0.0 -0.5

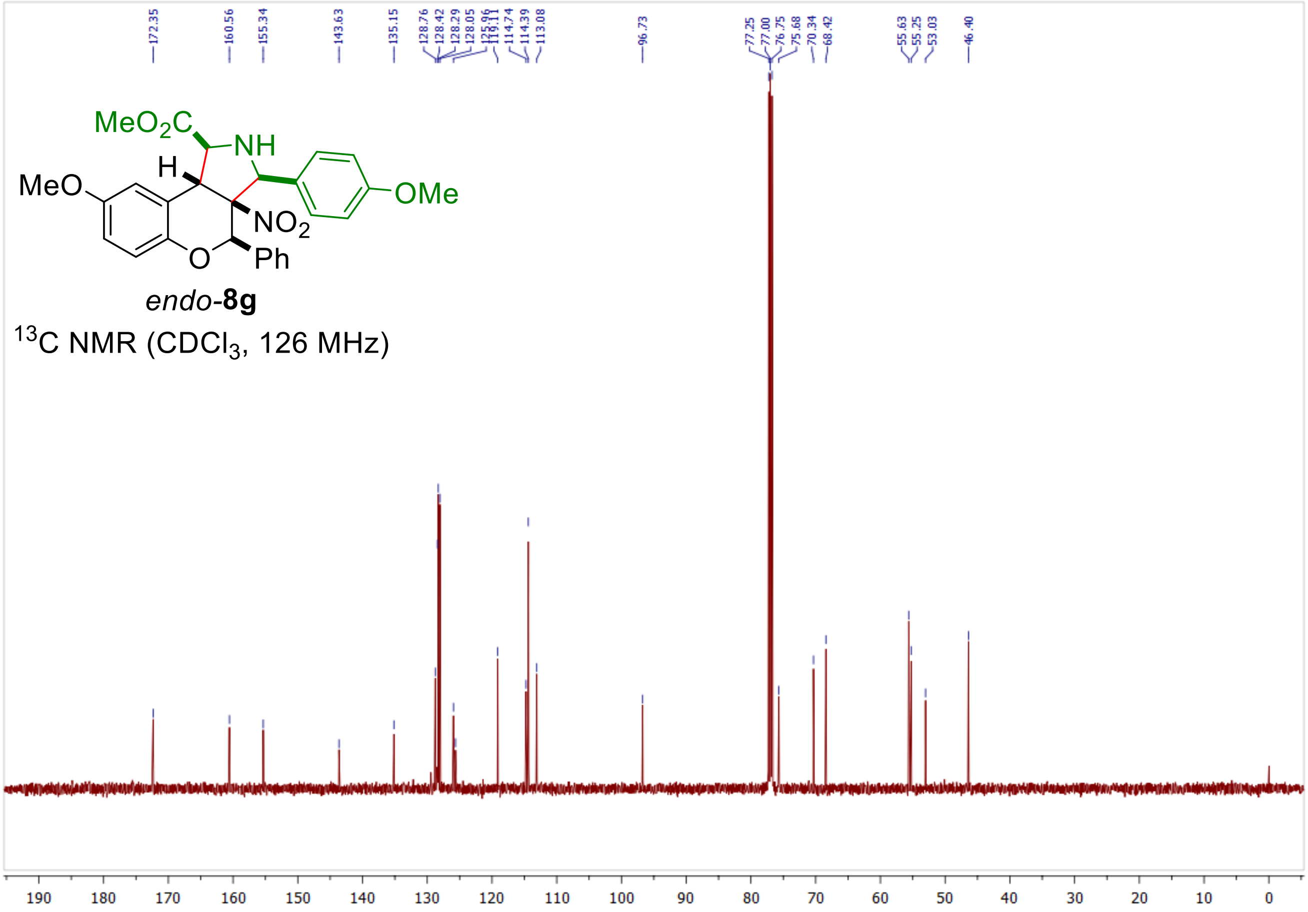
172.35
160.56 155.34
143.63
135.15
128.76 128.42 128.29 128.05
114.74 114.39 113.08
96.73
77.25 77.00 76.75 75.68 70.34 68.42
55.63 55.25 53.03
46.40
MeO2C
NH
H
MeO
OMe
NO2
O
Ph
endo-8g
13C NMR (CDCl3, 126 MHz)
190 180 170 160 150 140 130 120 110 100 90 80 70 60 50 40 30 20 10 0

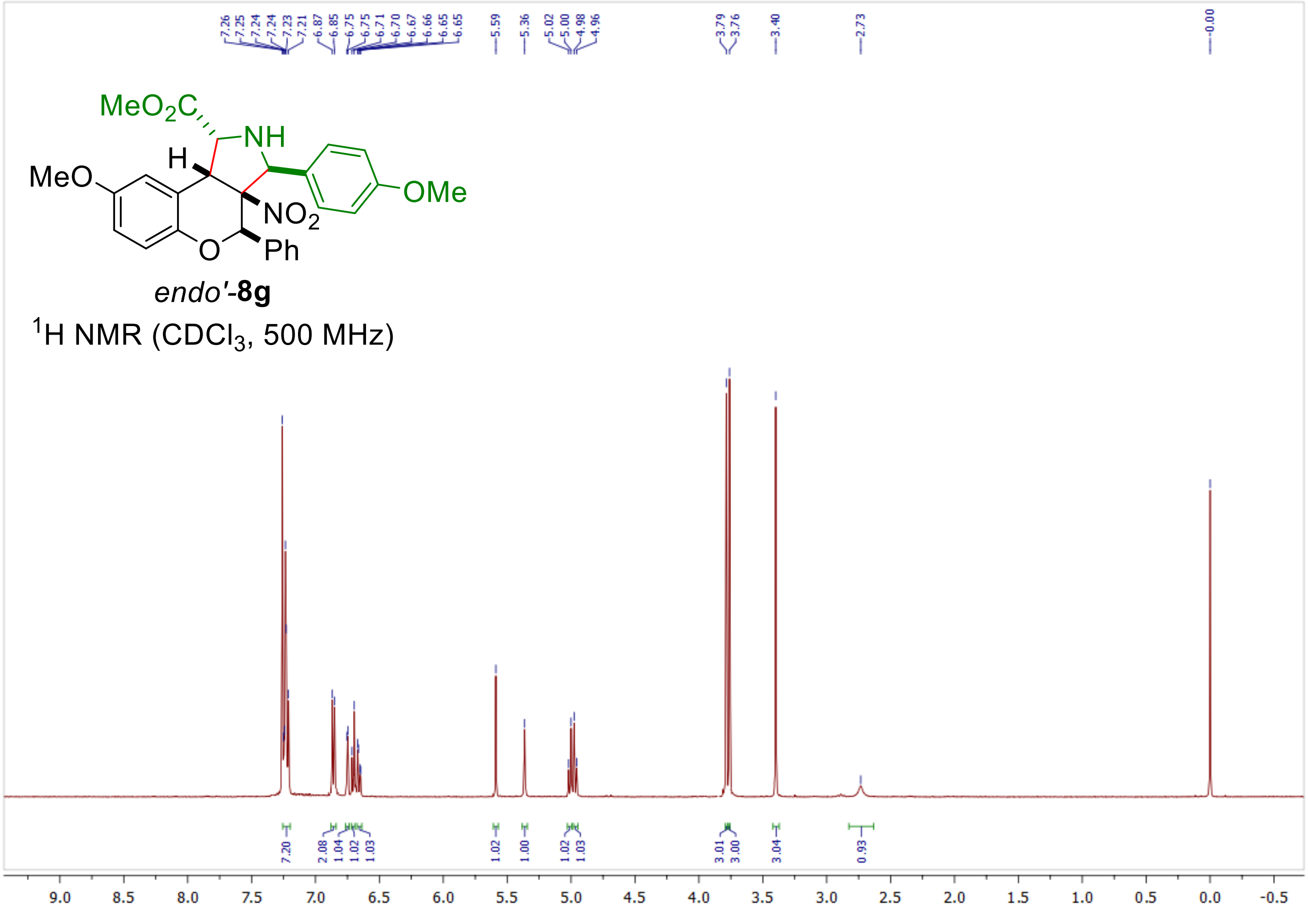
MeO$_2$C
NH
H
MeO
OMe
NO$_2$
O
Ph
endo'-**8g**
^{1}H NMR ($CDCl_3$, 500 MHz)

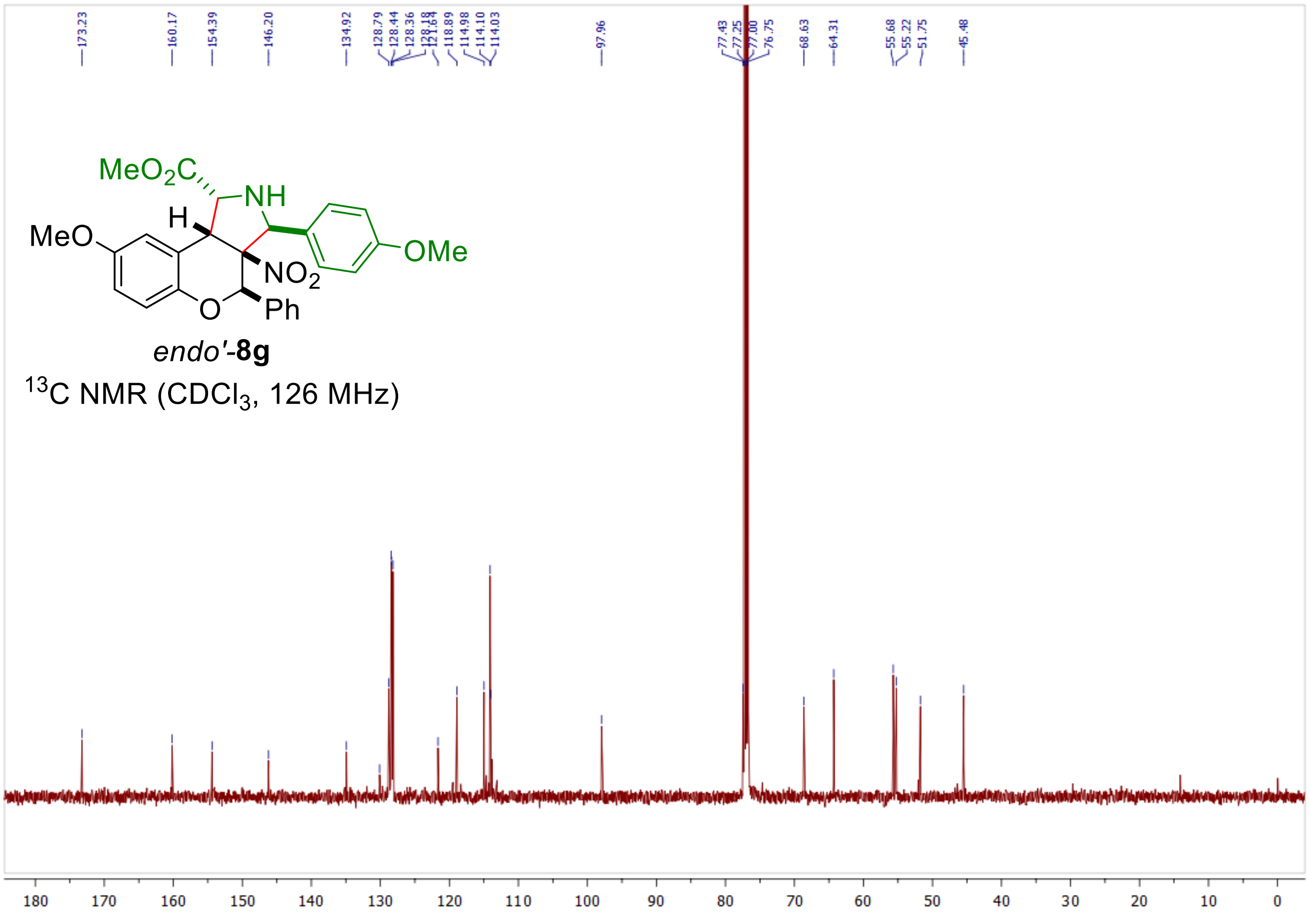
MeO$_2$C
NH
H
MeO
OMe
NO$_2$
O
Ph
endo'-**8g**
^{13}C NMR ($CDCl_3$, 126 MHz)

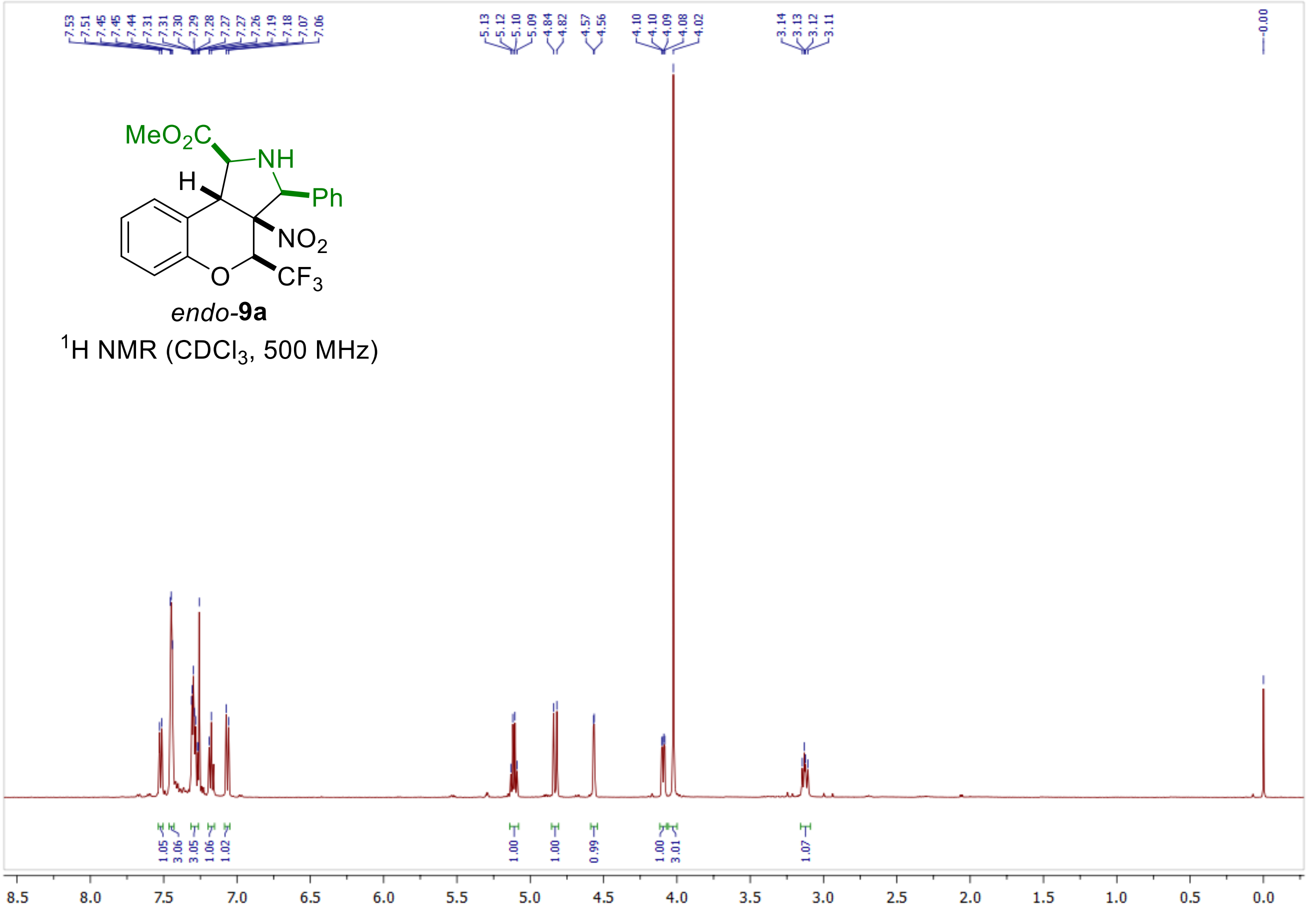

MeO2C
NH
H
Ph
NO2
O
CF3
endo-9a
1H NMR (CDCl3, 500 MHz)

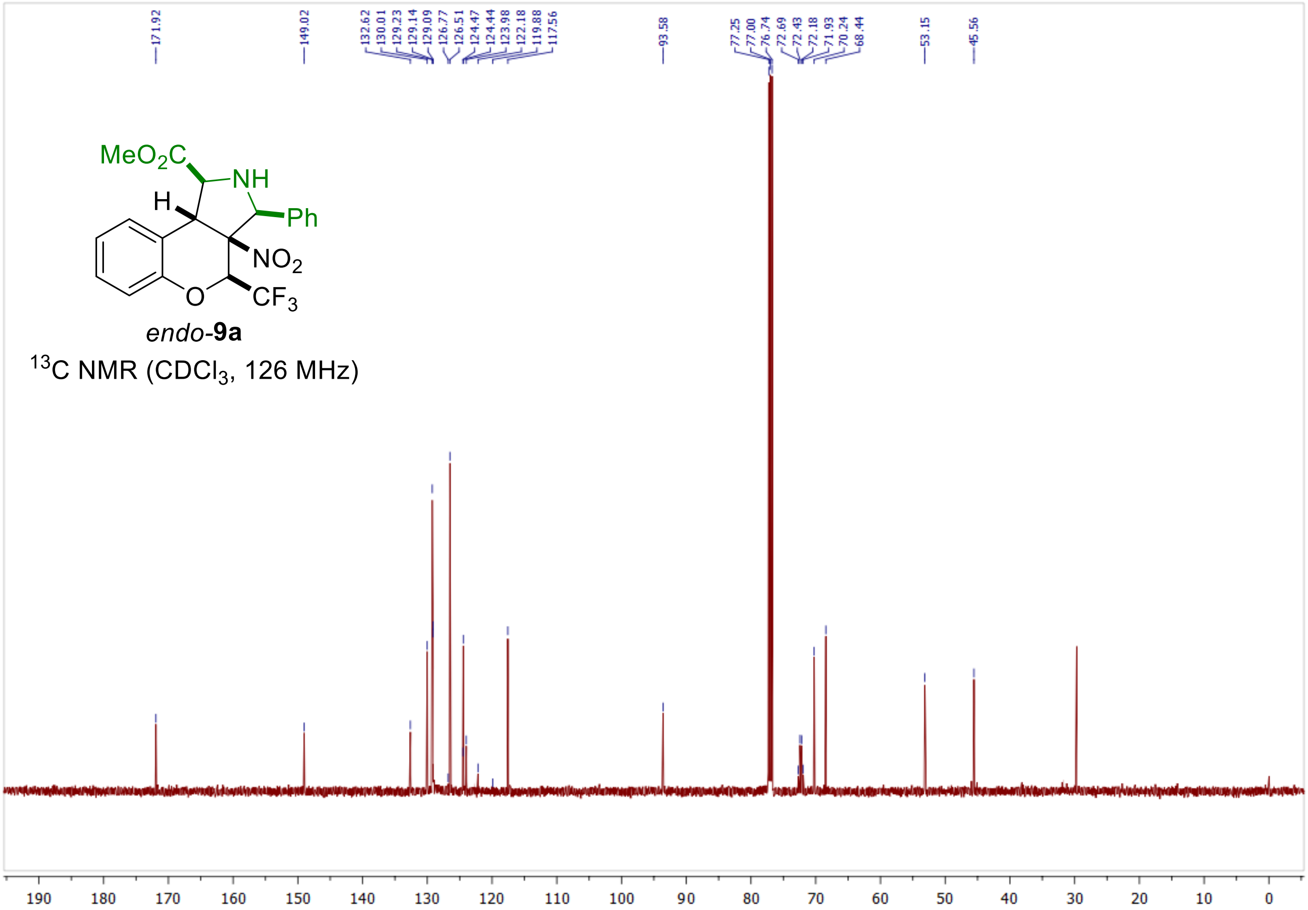

MeO2C
NH
H
Ph
NO2
O
CF3
endo-9a
13C NMR (CDCl3, 126 MHz)

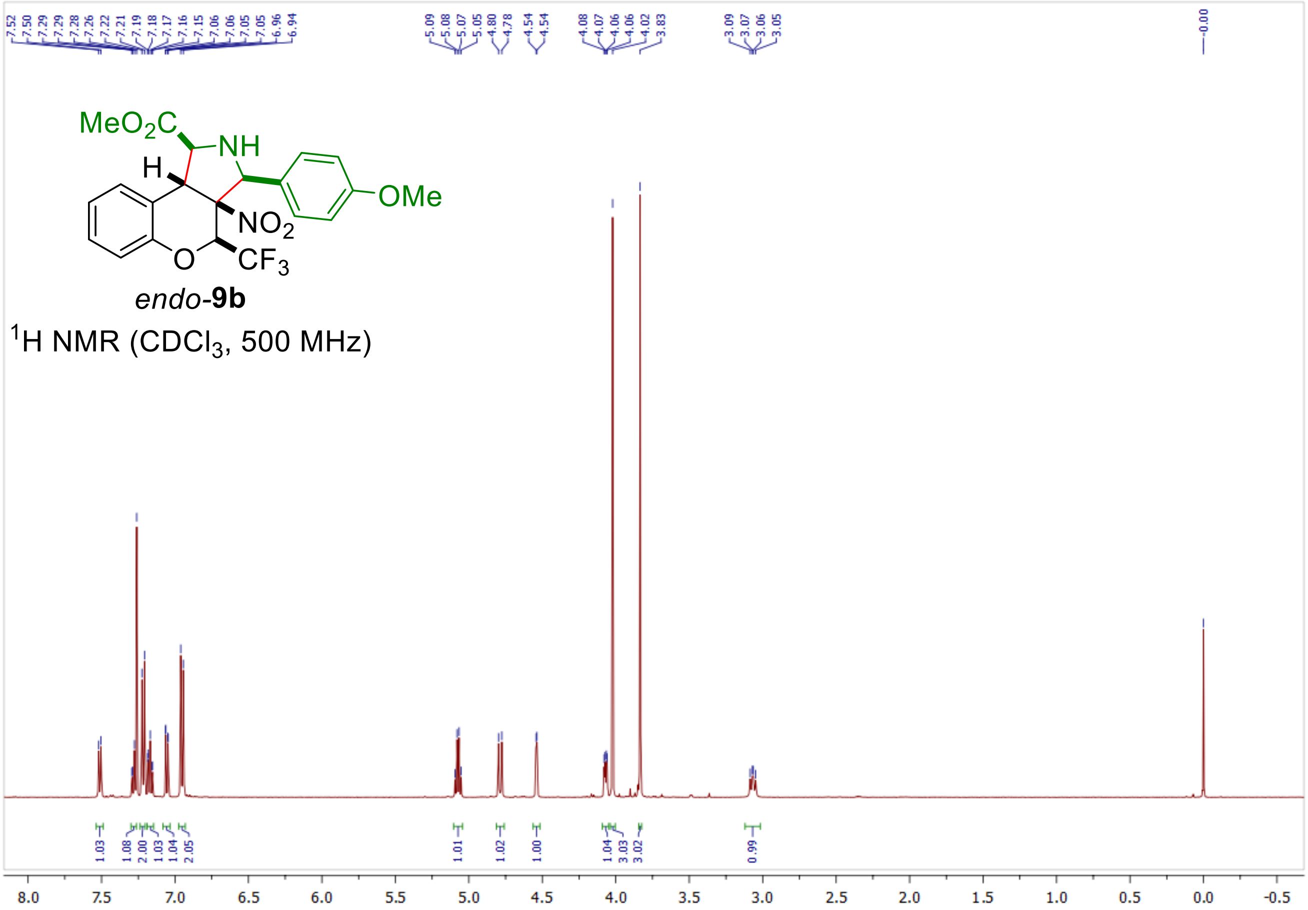

7.52 7.50 7.29 7.29 7.28 7.26 7.22 7.21 7.19 7.18 7.17 7.16 7.15 7.06 7.06 7.05 7.05 6.96 6.94
5.09 5.08 5.07 5.05 4.80 4.78 4.54 4.54
4.08 4.07 4.06 4.06 4.02 3.83
3.09 3.07 3.06 3.05
0.00
MeO2C
NH
H
OMe
NO2
O
CF3
endo-9b
1H NMR (CDCl3, 500 MHz)
1.03 1.08 2.00 1.03 1.04 2.05
1.01 1.02 1.00
1.04 3.03 3.02
0.99
8.0 7.5 7.0 6.5 6.0 5.5 5.0 4.5 4.0 3.5 3.0 2.5 2.0 1.5 1.0 0.5 0.0 -0.5

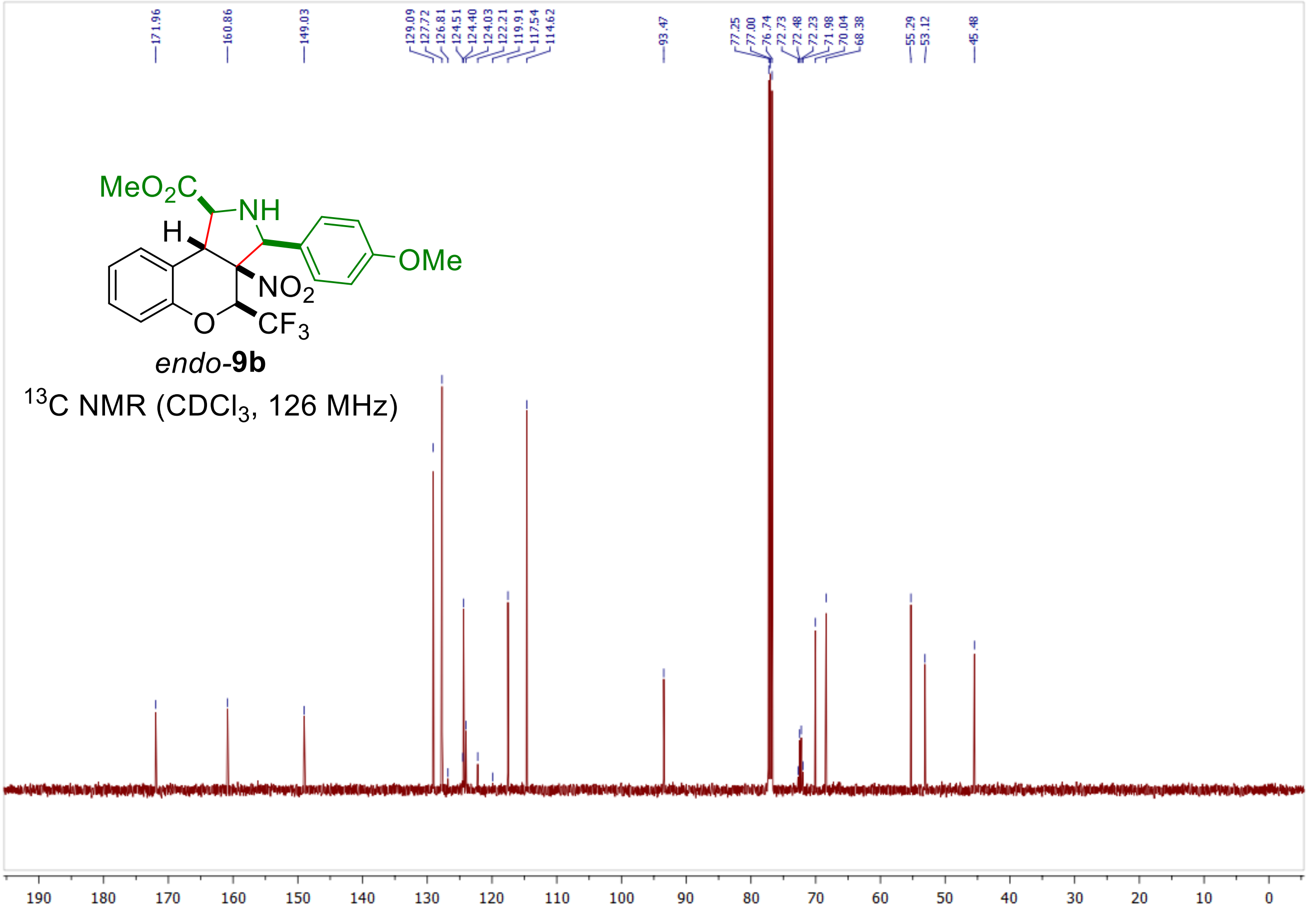

171.96
160.86
149.03
129.09 127.72 126.81 124.51 124.40 124.03 122.21 119.91 117.54 114.62
93.47
77.25 77.00 76.74 72.73 72.48 72.23 71.98 70.04 68.38
55.29 53.12
45.48
MeO2C
NH
H
OMe
NO2
O
CF3
endo-9b
13C NMR (CDCl3, 126 MHz)
190 180 170 160 150 140 130 120 110 100 90 80 70 60 50 40 30 20 10 0

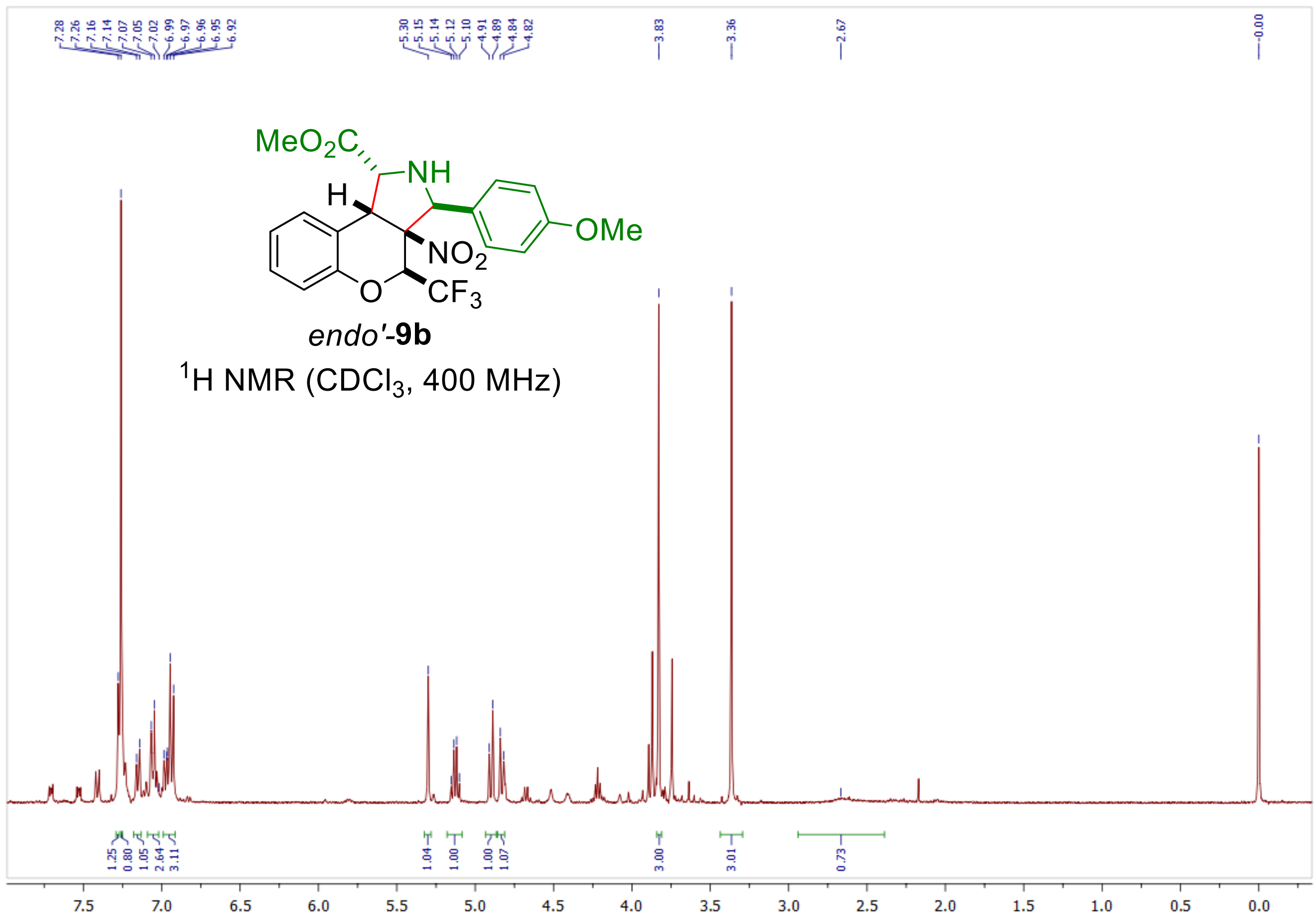
MeO2C
NH
H
OMe
NO2
O
CF3
endo'-9b
1H NMR (CDCl3, 400 MHz)

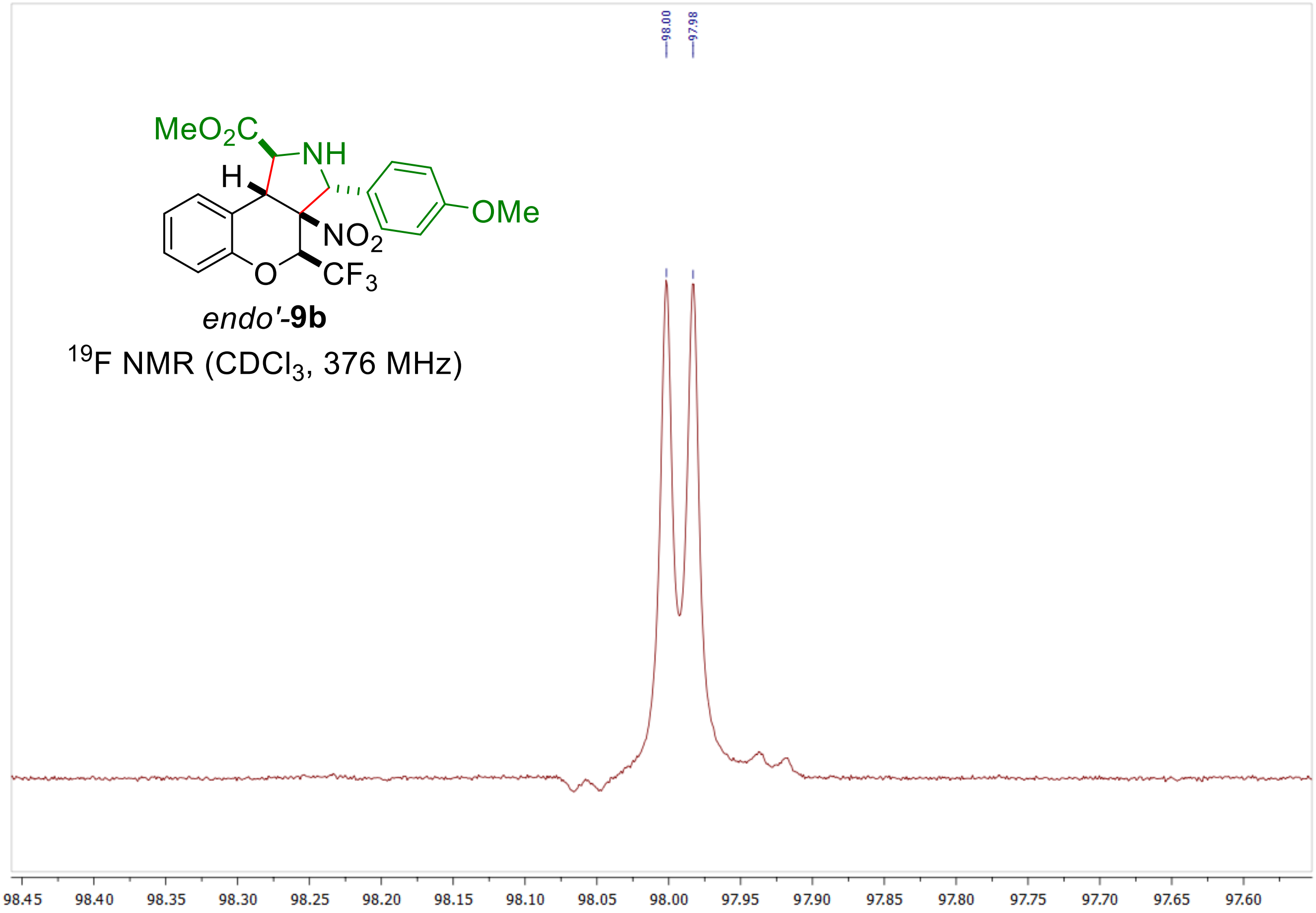
98.00
97.98
MeO2C
NH
H
OMe
NO2
O
CF3
endo'-9b
19F NMR (CDCl3, 376 MHz)

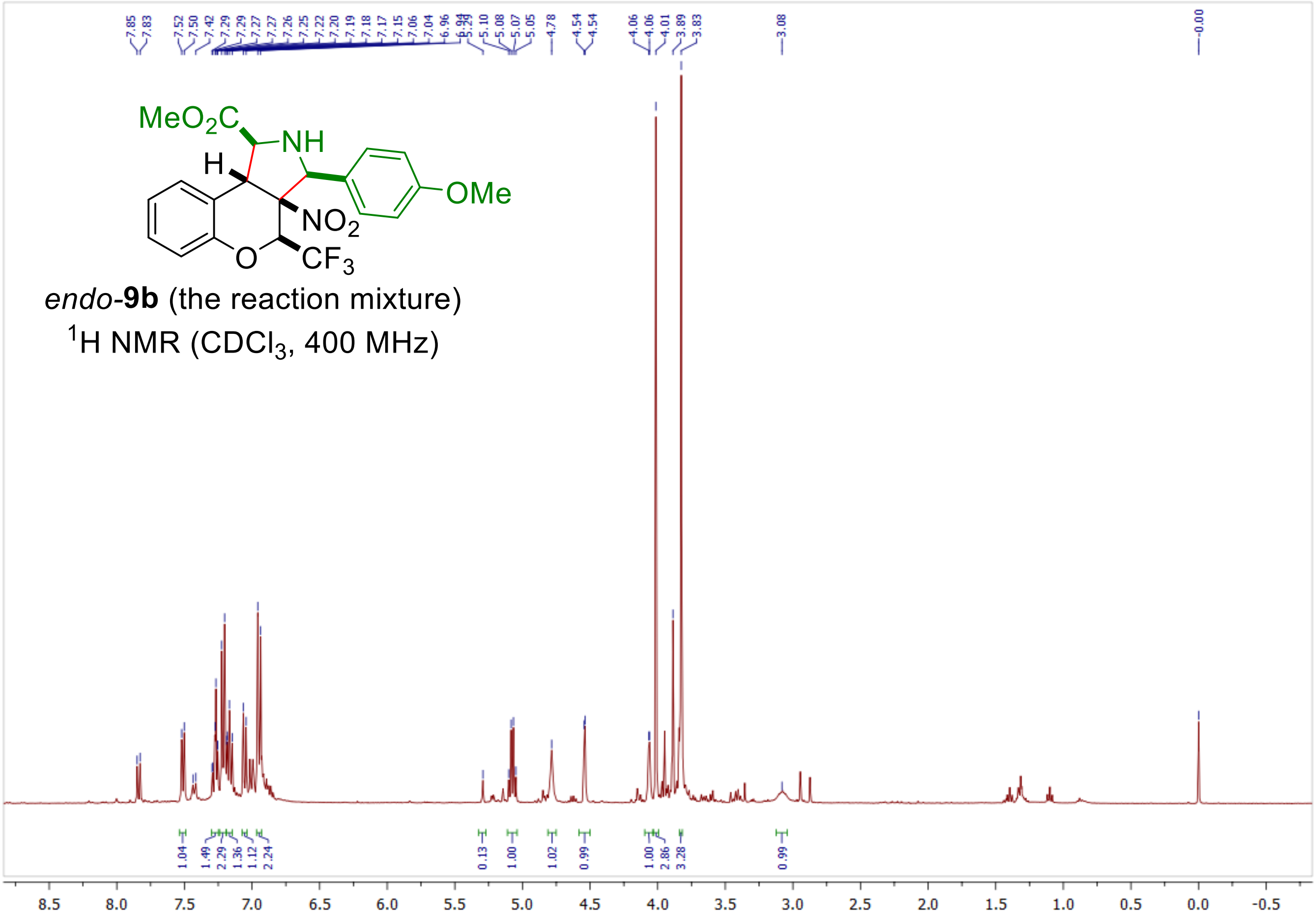
MeO2C
NH
H
OMe
NO2
O
CF3
endo-9b (the reaction mixture)
1H NMR (CDCl3, 400 MHz)

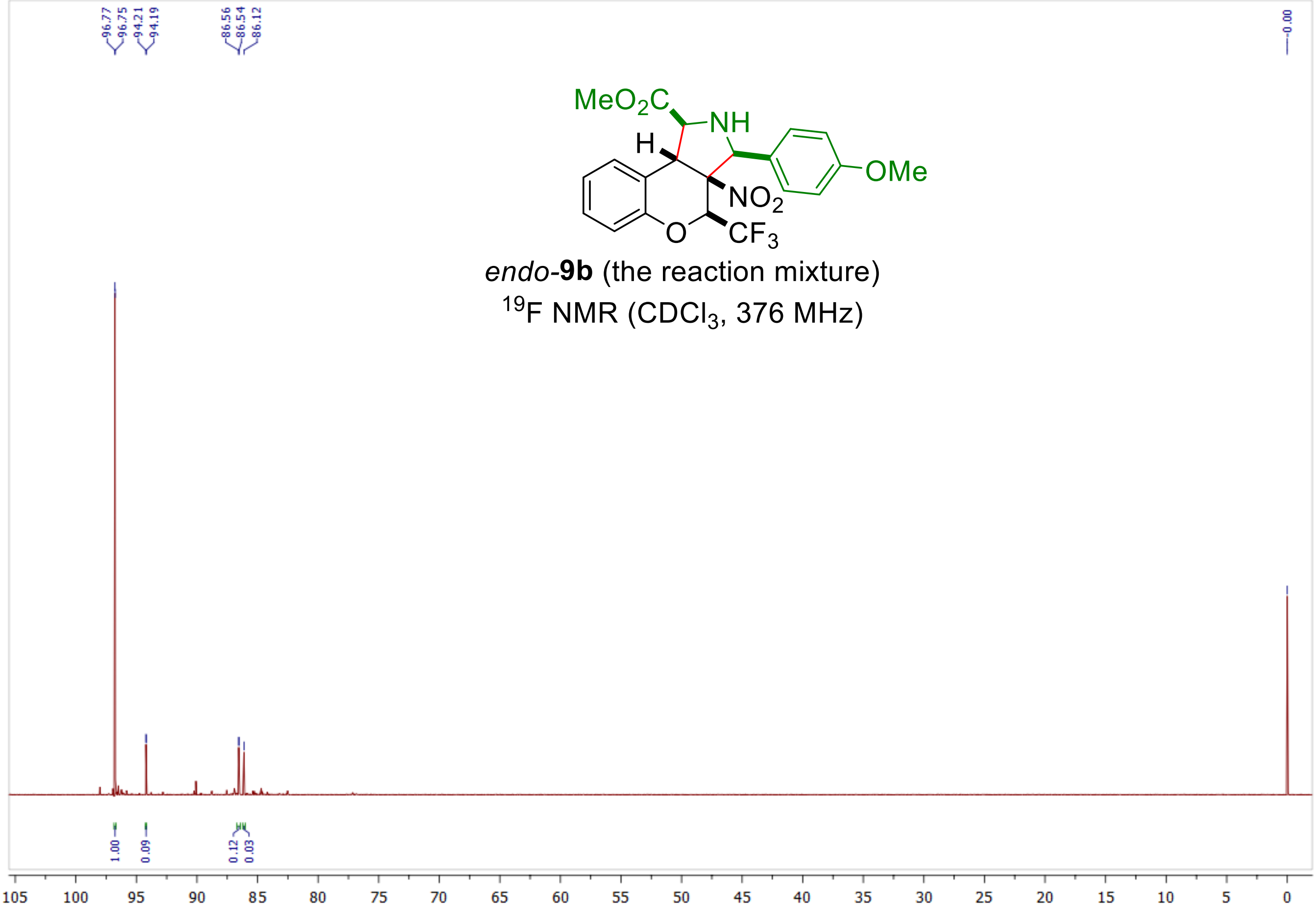
MeO2C
NH
H
OMe
NO2
O
CF3
endo-9b (the reaction mixture)
19F NMR (CDCl3, 376 MHz)

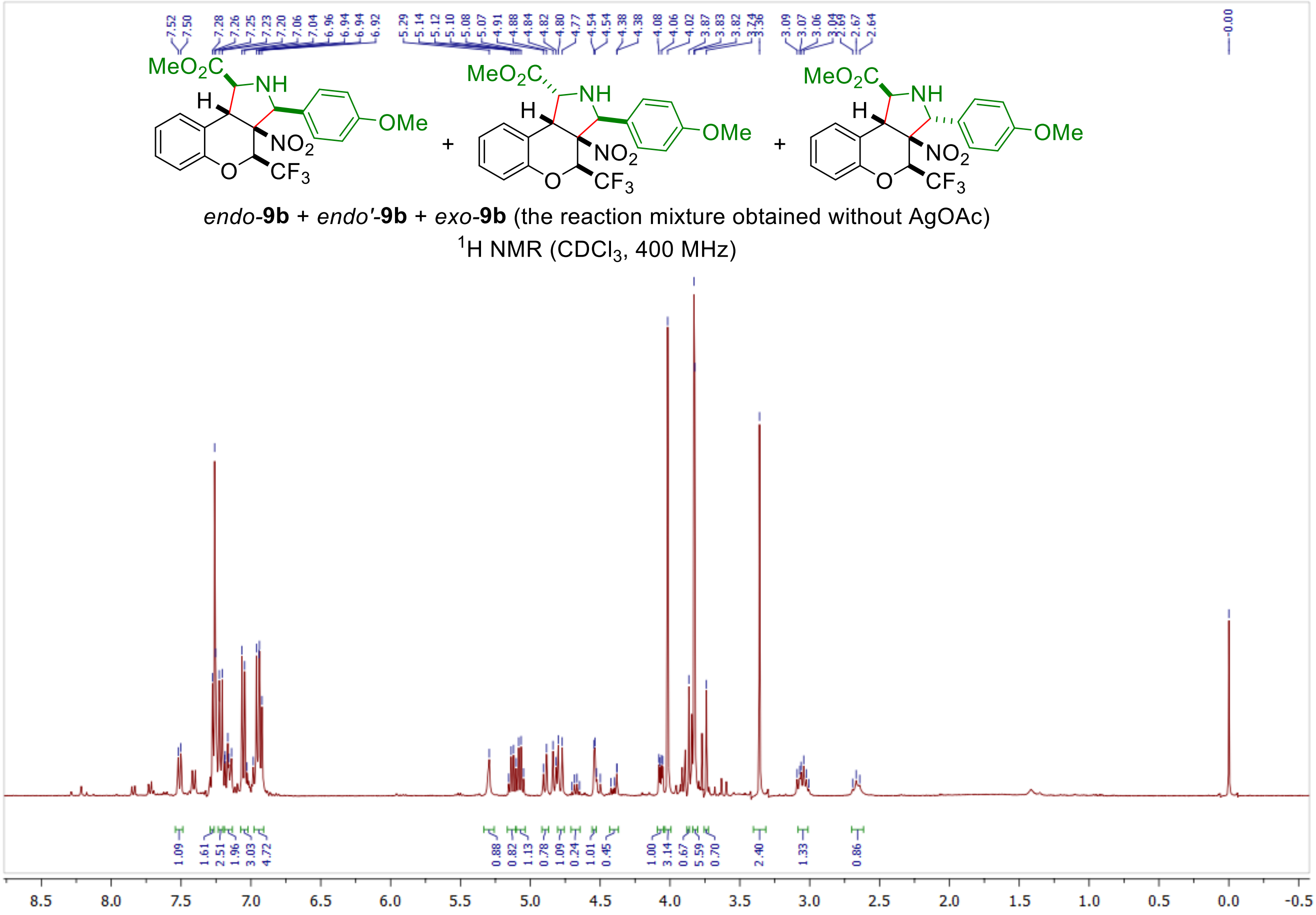

endo-9b + endo'-9b + exo-9b (the reaction mixture obtained without AgOAc)
1H NMR (CDCl3, 400 MHz)

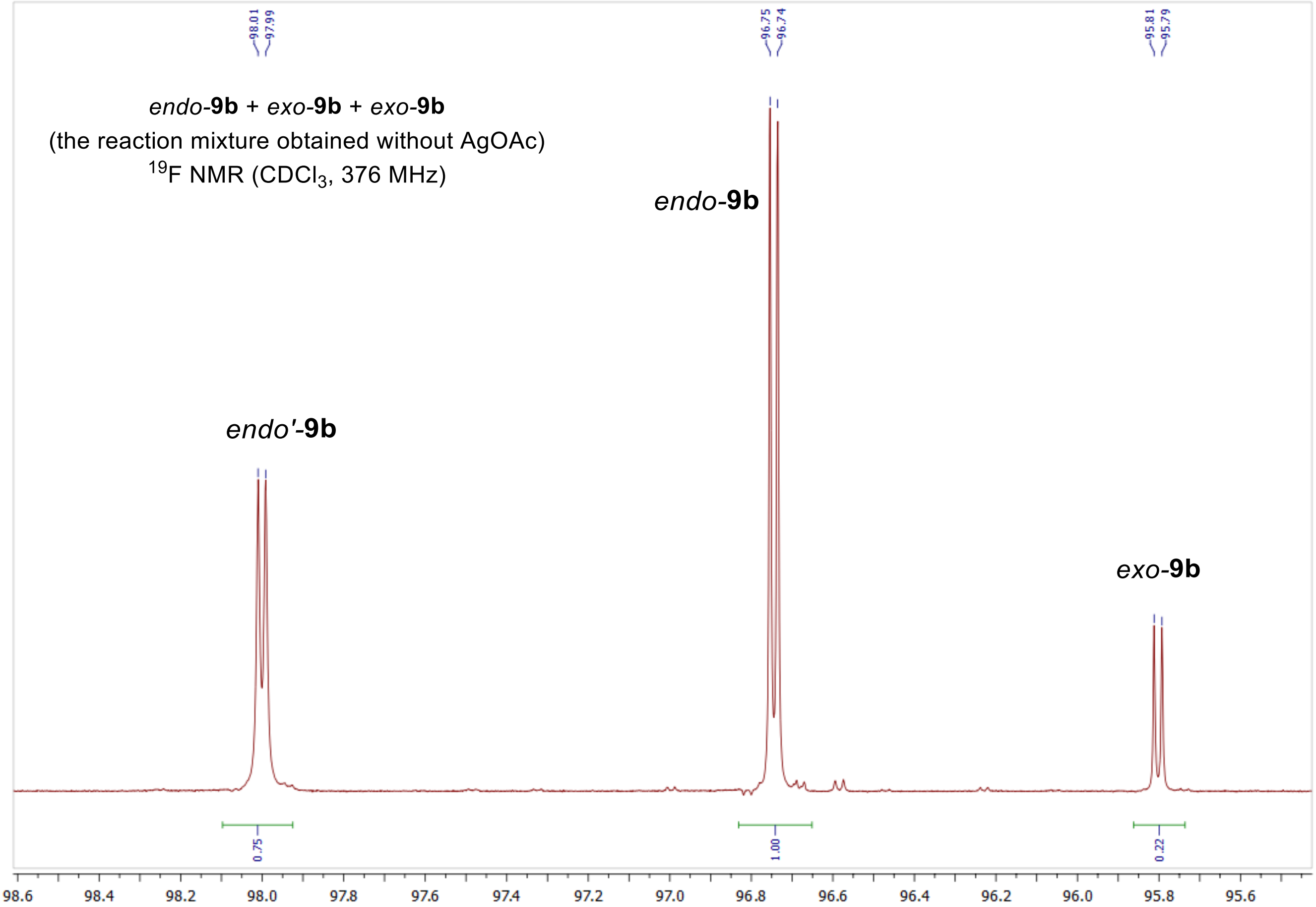

endo-9b + exo-9b + exo-9b
(the reaction mixture obtained without AgOAc)
19F NMR (CDCl3, 376 MHz)
endo-9b
endo'-9b
exo-9b

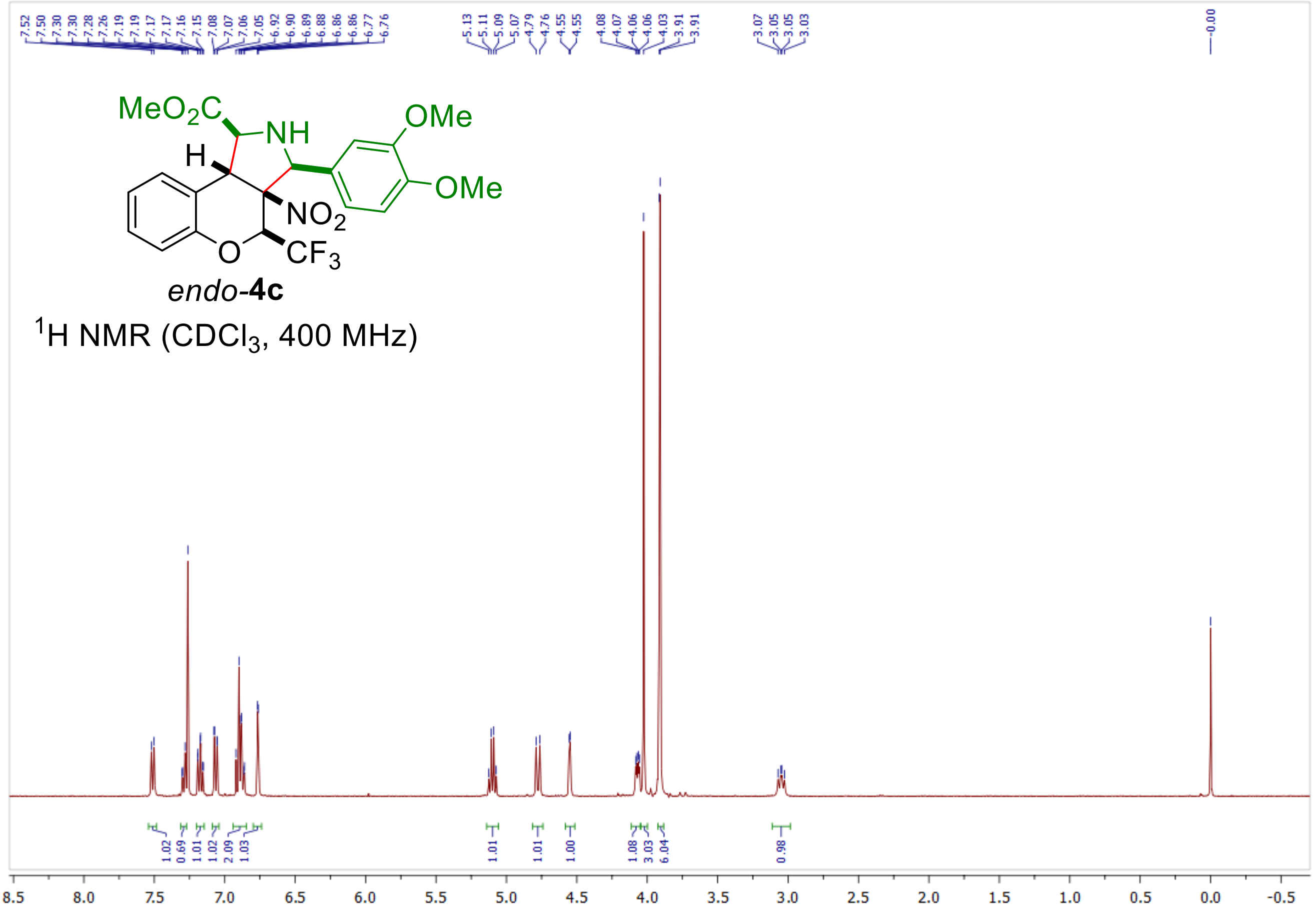
7.52 7.50 7.30 7.30 7.28 7.26 7.19 7.19 7.17 7.17 7.16 7.15 7.08 7.07 7.06 7.05 6.92 6.90 6.89 6.88 6.86 6.86 6.77 6.76
5.13 5.11 5.09 5.07 4.79 4.76 4.55 4.55 4.08 4.07 4.06 4.06 4.03 3.91 3.91
3.07 3.05 3.05 3.03
0.00
MeO2C
NH
H
OMe
OMe
NO2
O
CF3
endo-4c
1H NMR (CDCl3, 400 MHz)
1.02 0.69 1.01 1.02 2.09 1.03
1.01 1.01 1.00 1.08 3.03 6.04 0.98
8.5 8.0 7.5 7.0 6.5 6.0 5.5 5.0 4.5 4.0 3.5 3.0 2.5 2.0 1.5 1.0 0.5 0.0 -0.5

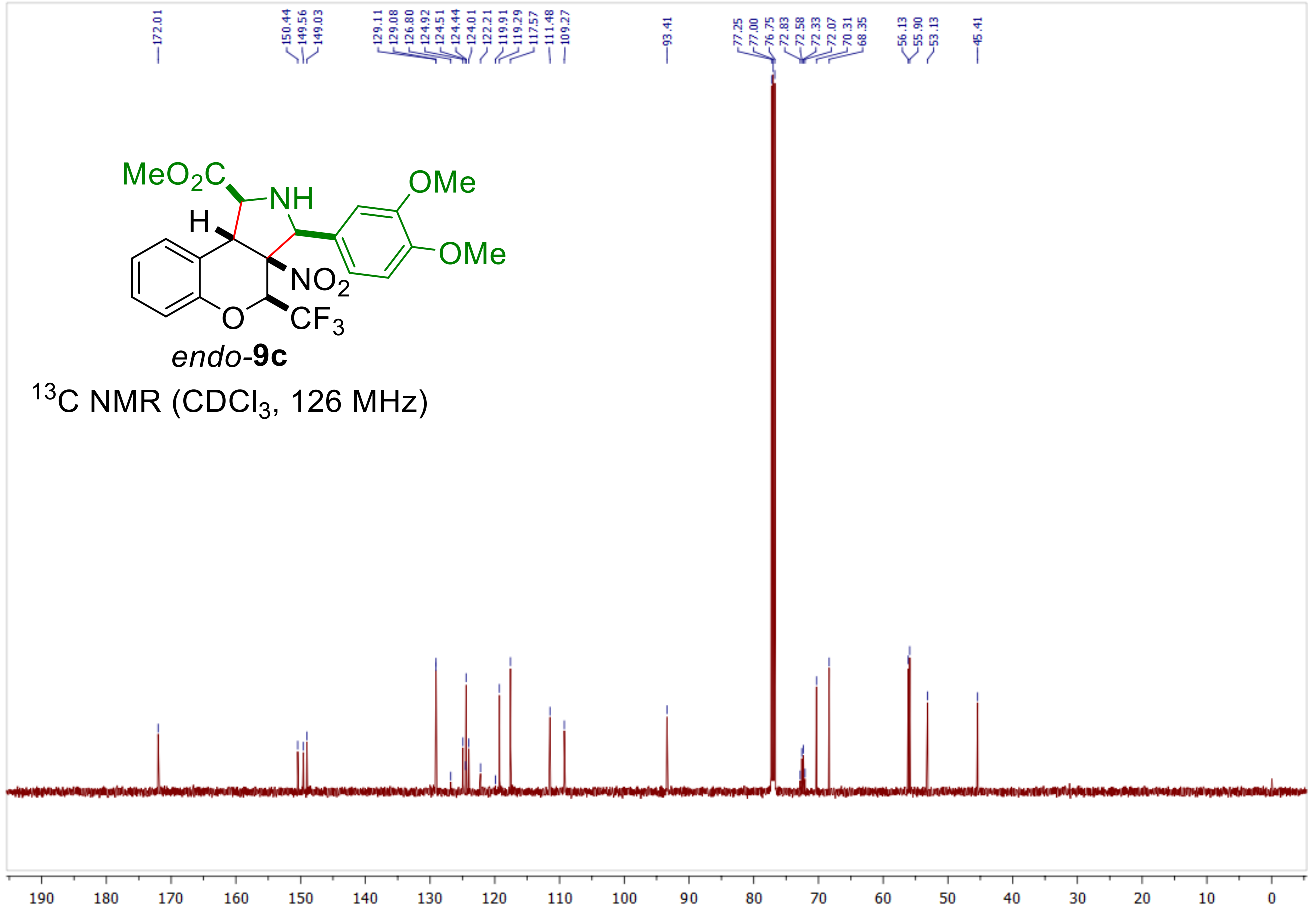
172.01
150.44 149.56 149.03
129.11 129.08 126.80 124.92 124.51 124.44 124.01 122.21 119.91 119.29 117.57 111.48 109.27
93.41
77.25 77.00 76.75 72.83 72.58 72.33 72.07 70.31 68.35
56.13 55.90 53.13
45.41
MeO2C
NH
H
OMe
OMe
NO2
O
CF3
endo-9c
13C NMR (CDCl3, 126 MHz)
190 180 170 160 150 140 130 120 110 100 90 80 70 60 50 40 30 20 10 0

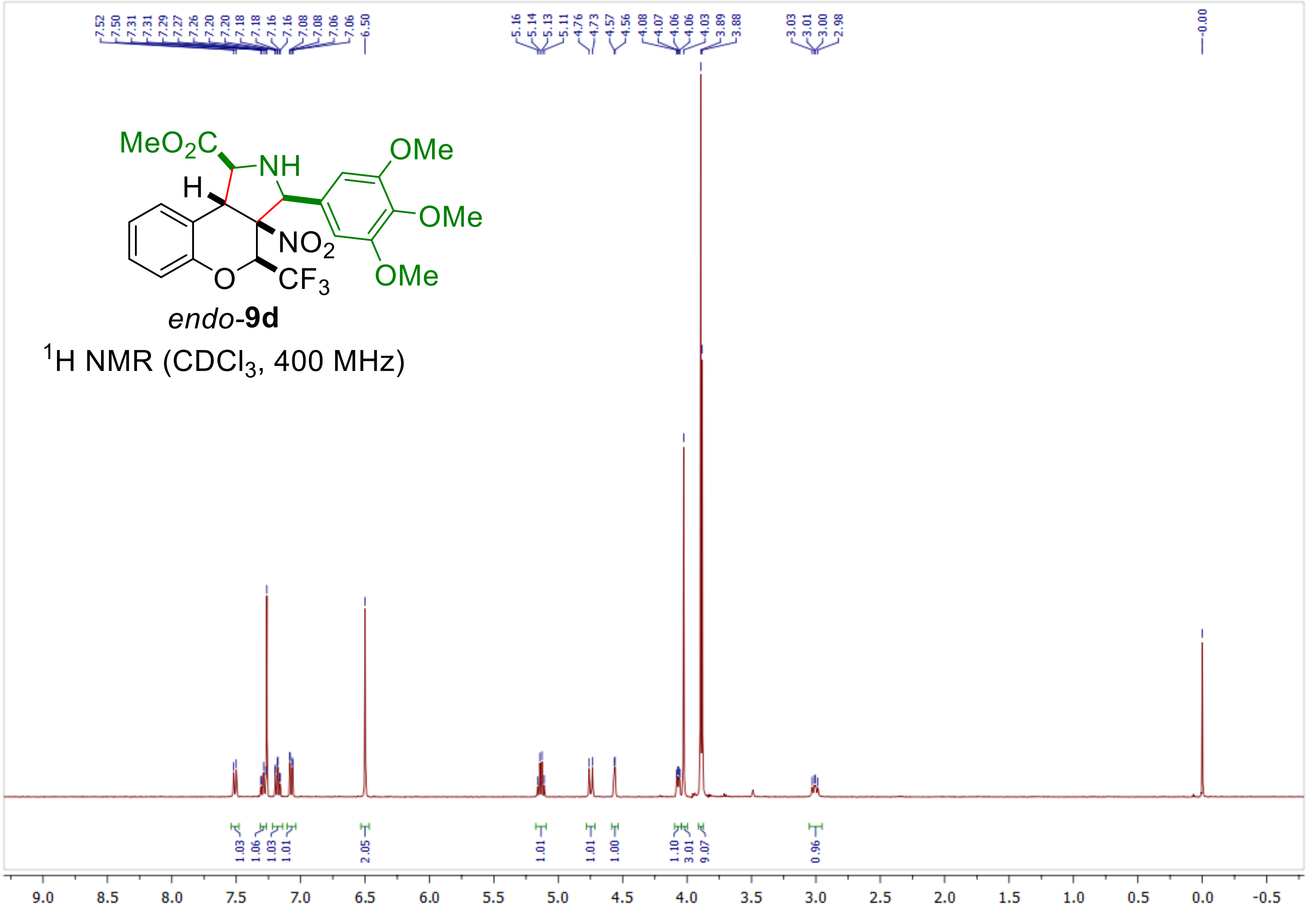
7.52 7.50 7.31 7.31 7.29 7.27 7.26 7.20 7.20 7.18 7.18 7.16 7.16 7.08 7.08 7.06 7.06 6.50
5.16 5.14 5.13 5.11 4.76 4.73 4.57 4.56 4.08 4.07 4.06 4.06 4.03 3.89 3.88
3.03 3.01 3.00 2.98
0.00
MeO2C
NH
H
OMe
OMe
NO2
O
CF3
OMe
endo-9d
1H NMR (CDCl3, 400 MHz)
1.03 1.06 1.03 1.01 2.05 1.01 1.01 1.00 1.10 3.01 9.07 0.96
9.0 8.5 8.0 7.5 7.0 6.5 6.0 5.5 5.0 4.5 4.0 3.5 3.0 2.5 2.0 1.5 1.0 0.5 0.0 -0.5

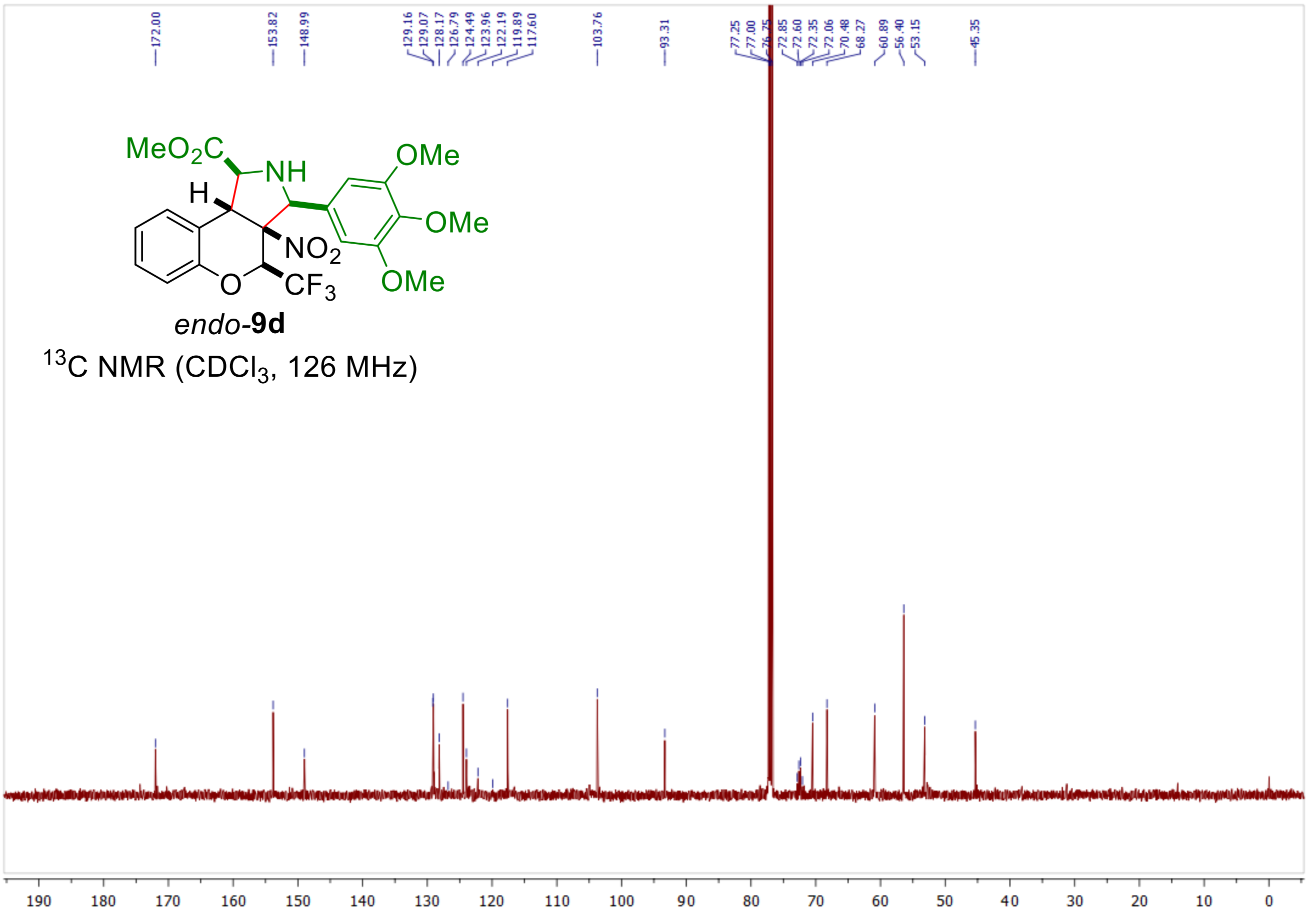
172.00
153.82 148.99
129.16 129.07 128.17 126.79 124.49 123.96 122.19 119.89 117.60
103.76
93.31
77.25 77.00 76.75 72.85 72.60 72.35 72.06 70.48 68.27 60.89 56.40 53.15
45.35
MeO2C
NH
H
OMe
OMe
NO2
O
CF3
OMe
endo-9d
13C NMR (CDCl3, 126 MHz)
190 180 170 160 150 140 130 120 110 100 90 80 70 60 50 40 30 20 10 0

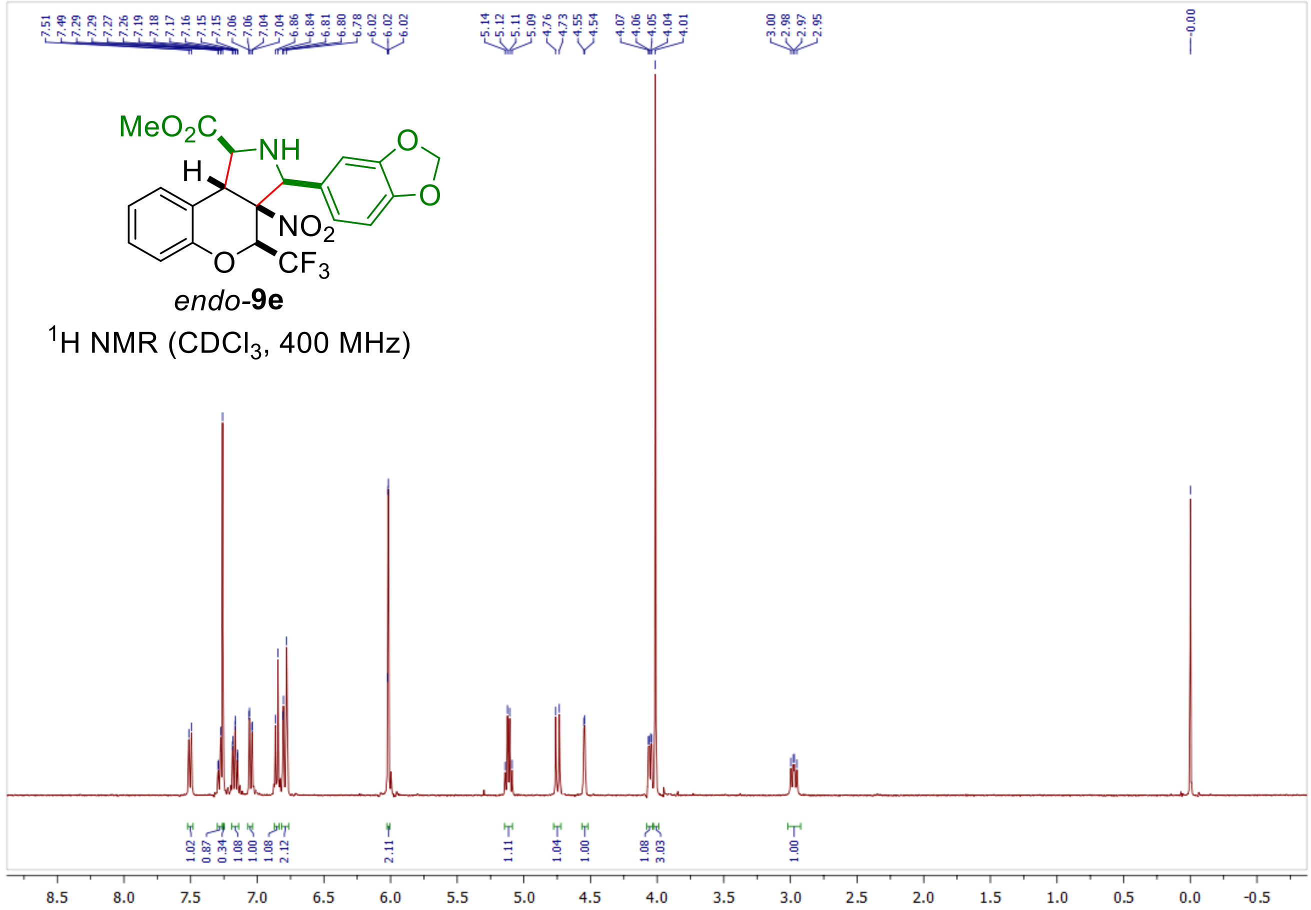

MeO2C
NH
H
NO2
O
CF3
O
O
endo-9e
1H NMR (CDCl3, 400 MHz)

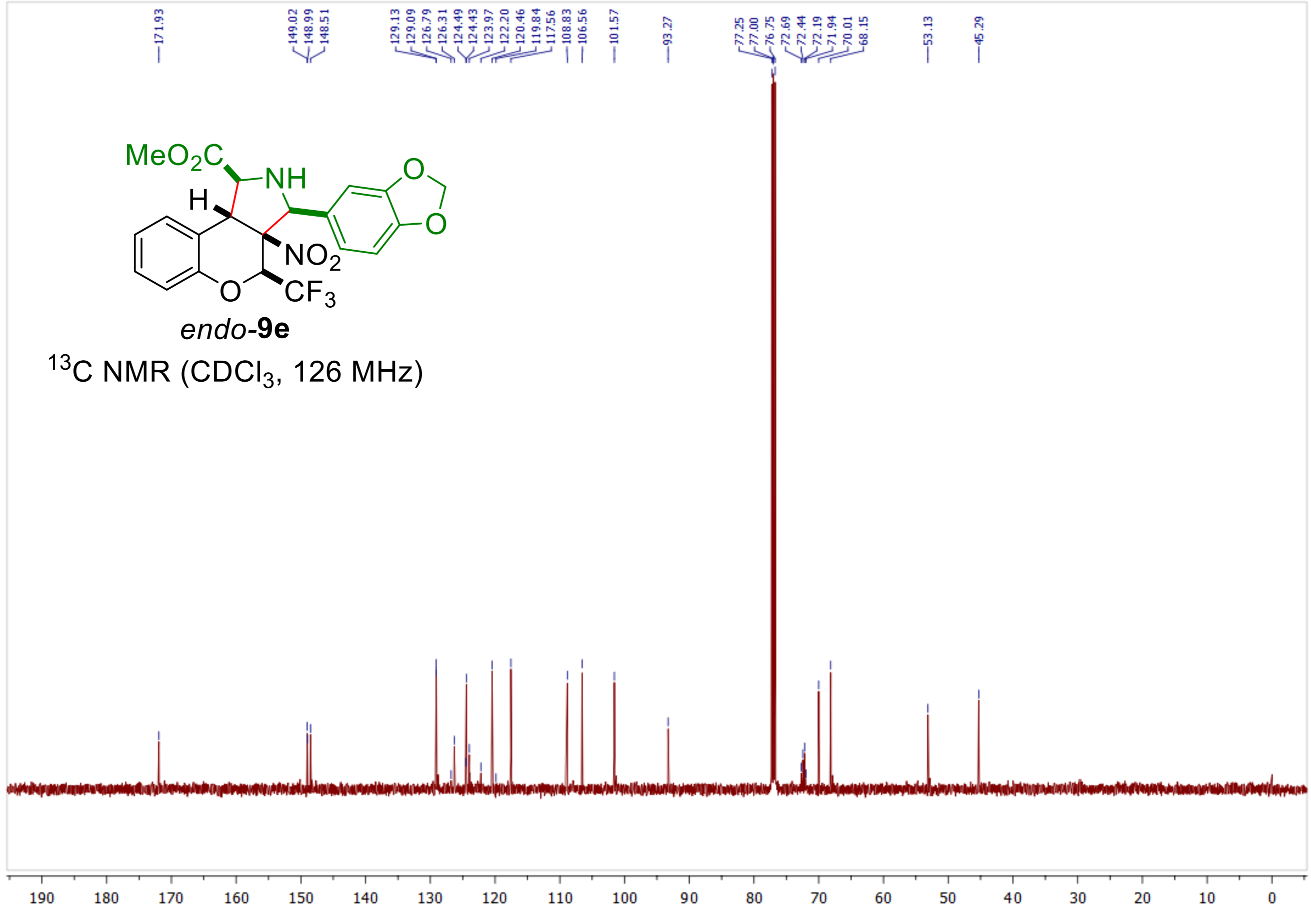

MeO2C
NH
H
NO2
O
CF3
O
O
endo-9e
13C NMR (CDCl3, 126 MHz)

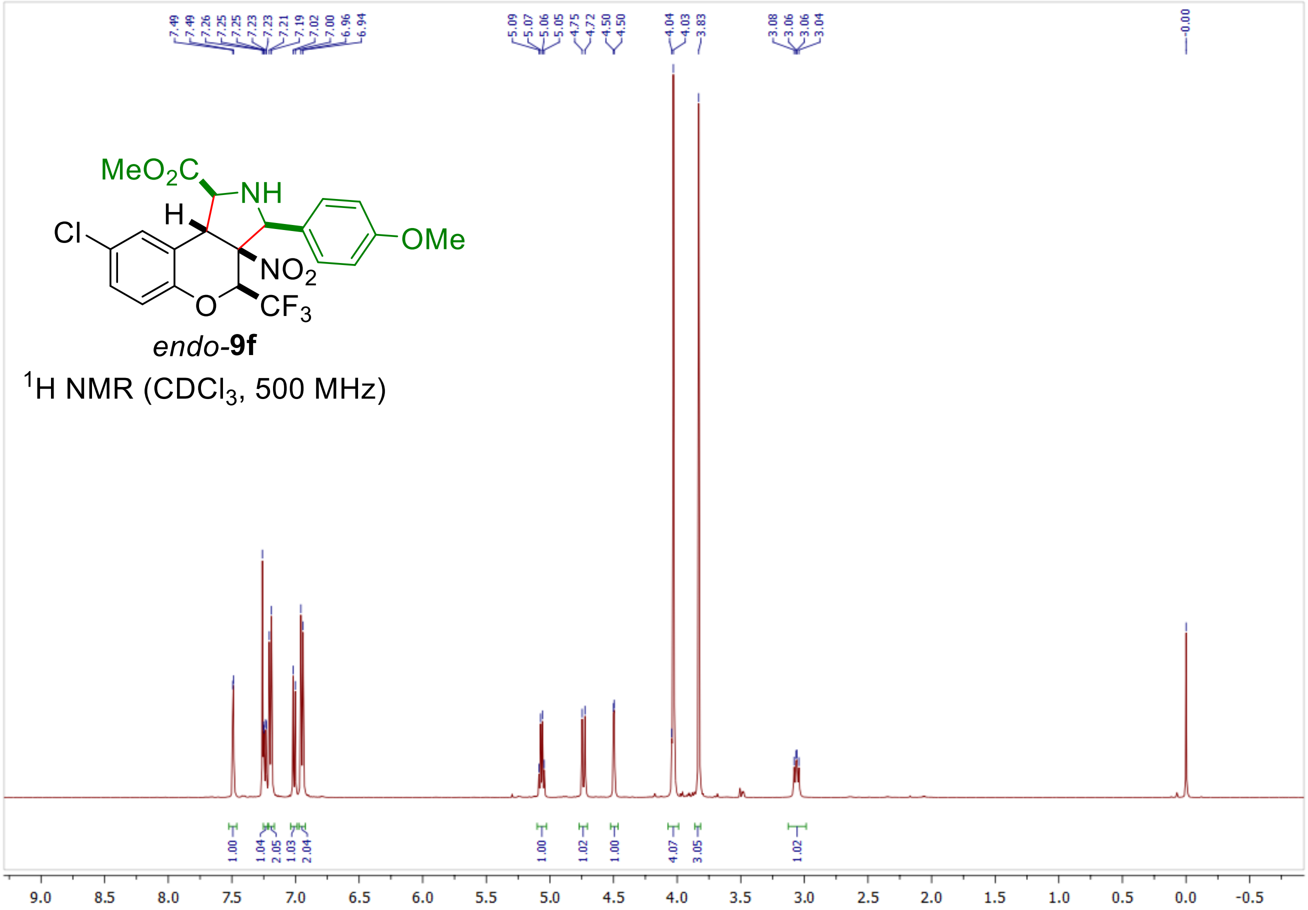
7.49
7.49
7.26
7.25
7.23
7.23
7.21
7.19
7.02
7.00
6.96
6.94
5.09
5.07
5.06
5.05
4.75
4.72
4.50
4.50
4.04
4.03
3.83
3.08
3.06
3.06
3.04
0.00
MeO2C
NH
H
Cl
OMe
NO2
O
CF3
endo-9f
1H NMR (CDCl3, 500 MHz)
1.00
1.04
2.05
1.03
2.04
1.00
1.02
1.00
4.07
3.05
1.02
9.0 8.5 8.0 7.5 7.0 6.5 6.0 5.5 5.0 4.5 4.0 3.5 3.0 2.5 2.0 1.5 1.0 0.5 0.0 -0.5

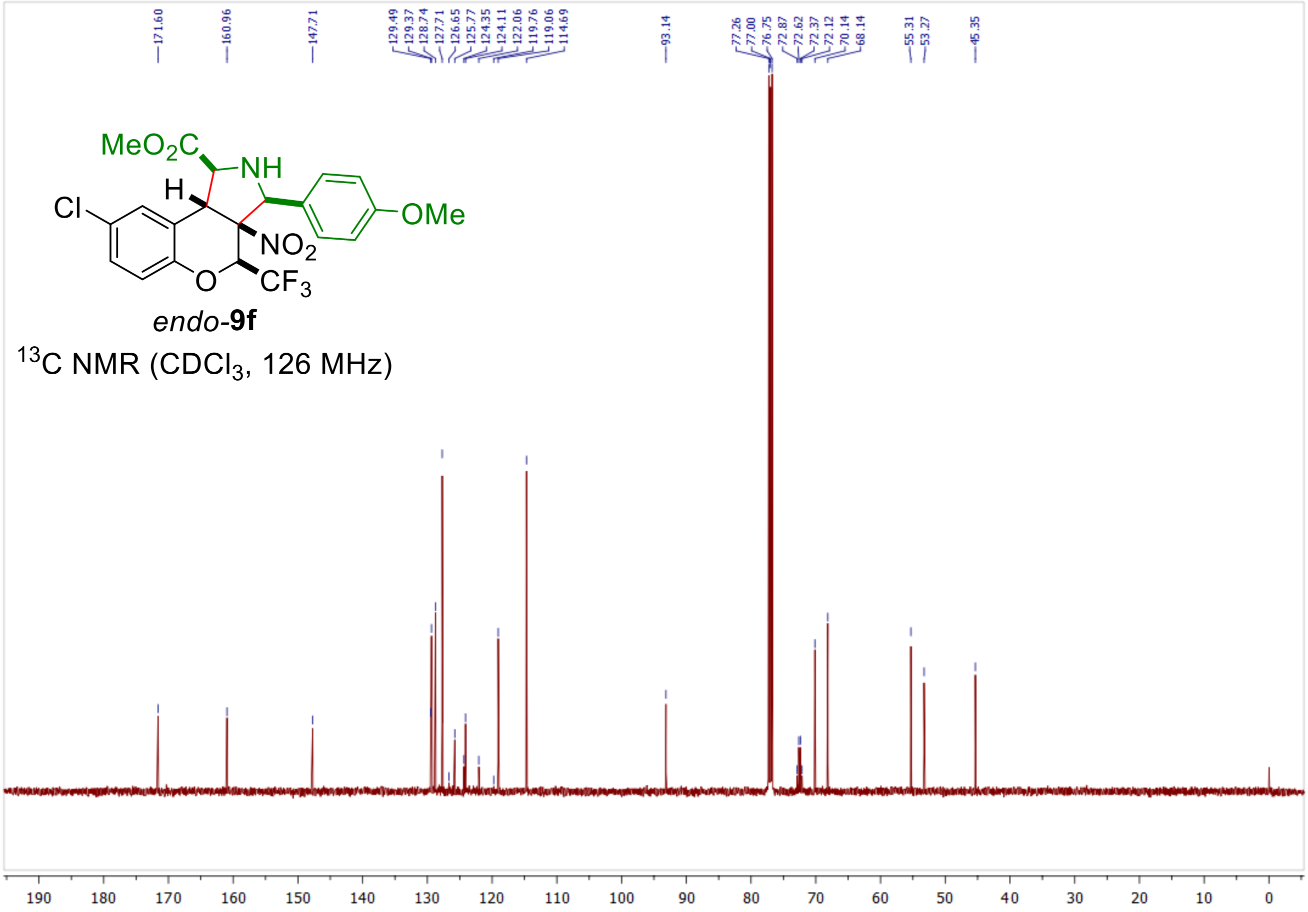
171.60
160.96
147.71
129.49
129.37
128.74
127.71
126.65
125.77
124.35
124.11
122.06
119.76
119.06
114.69
93.14
77.26
77.00
76.75
72.87
72.62
72.37
72.12
70.14
68.14
55.31
53.27
45.35
MeO2C
NH
H
Cl
OMe
NO2
O
CF3
endo-9f
13C NMR (CDCl3, 126 MHz)
190 180 170 160 150 140 130 120 110 100 90 80 70 60 50 40 30 20 10 0

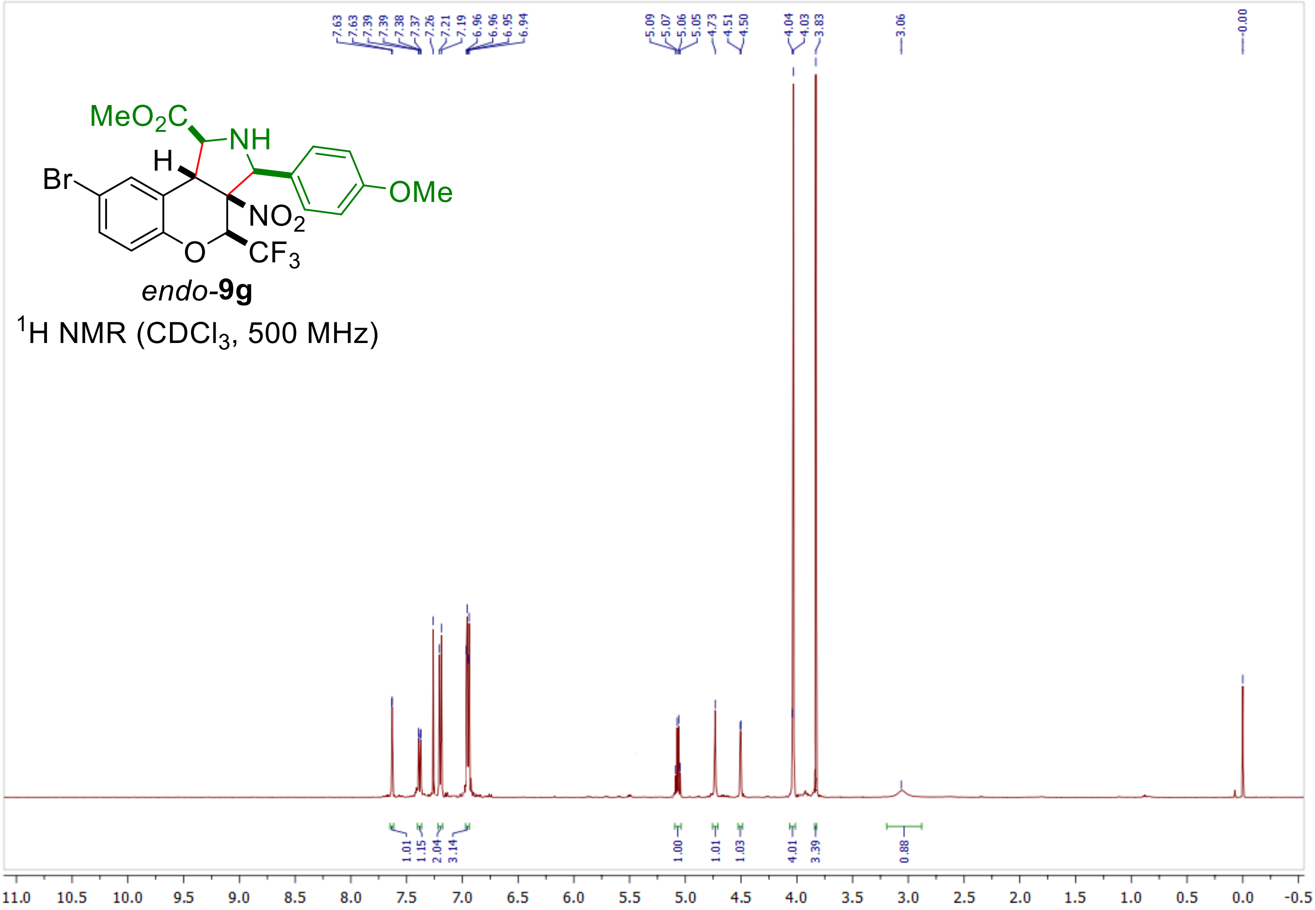
MeO2C
NH
H
Br
OMe
NO2
O
CF3
endo-9g
1H NMR (CDCl3, 500 MHz)
7.63
7.63
7.39
7.39
7.38
7.37
7.26
7.21
7.19
6.96
6.96
6.95
6.94
5.09
5.07
5.06
5.05
4.73
4.51
4.50
4.04
4.03
3.83
3.06
0.00
1.01
1.15
2.04
3.14
1.00
1.01
1.03
4.01
3.39
0.88
11.0
10.5
10.0
9.5
9.0
8.5
8.0
7.5
7.0
6.5
6.0
5.5
5.0
4.5
4.0
3.5
3.0
2.5
2.0
1.5
1.0
0.5
0.0
-0.5

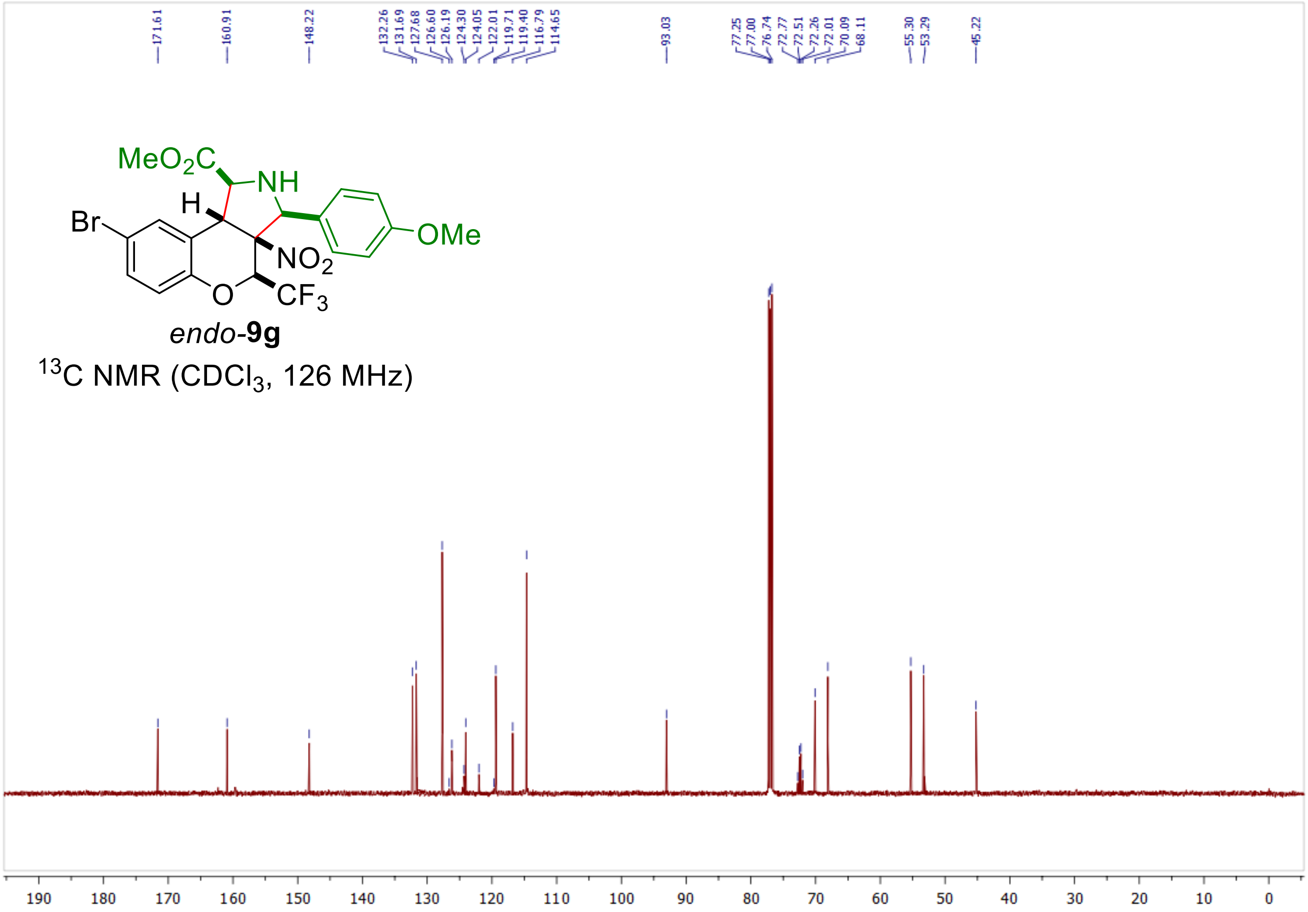
MeO2C
NH
H
Br
OMe
NO2
O
CF3
endo-9g
13C NMR (CDCl3, 126 MHz)
171.61
160.91
148.22
132.26
131.69
127.68
126.60
126.19
124.30
124.05
122.01
119.71
119.40
116.79
114.65
93.03
77.25
77.00
76.74
72.77
72.51
72.26
72.01
70.09
68.11
55.30
53.29
45.22
190
180
170
160
150
140
130
120
110
100
90
80
70
60
50
40
30
20
10
0

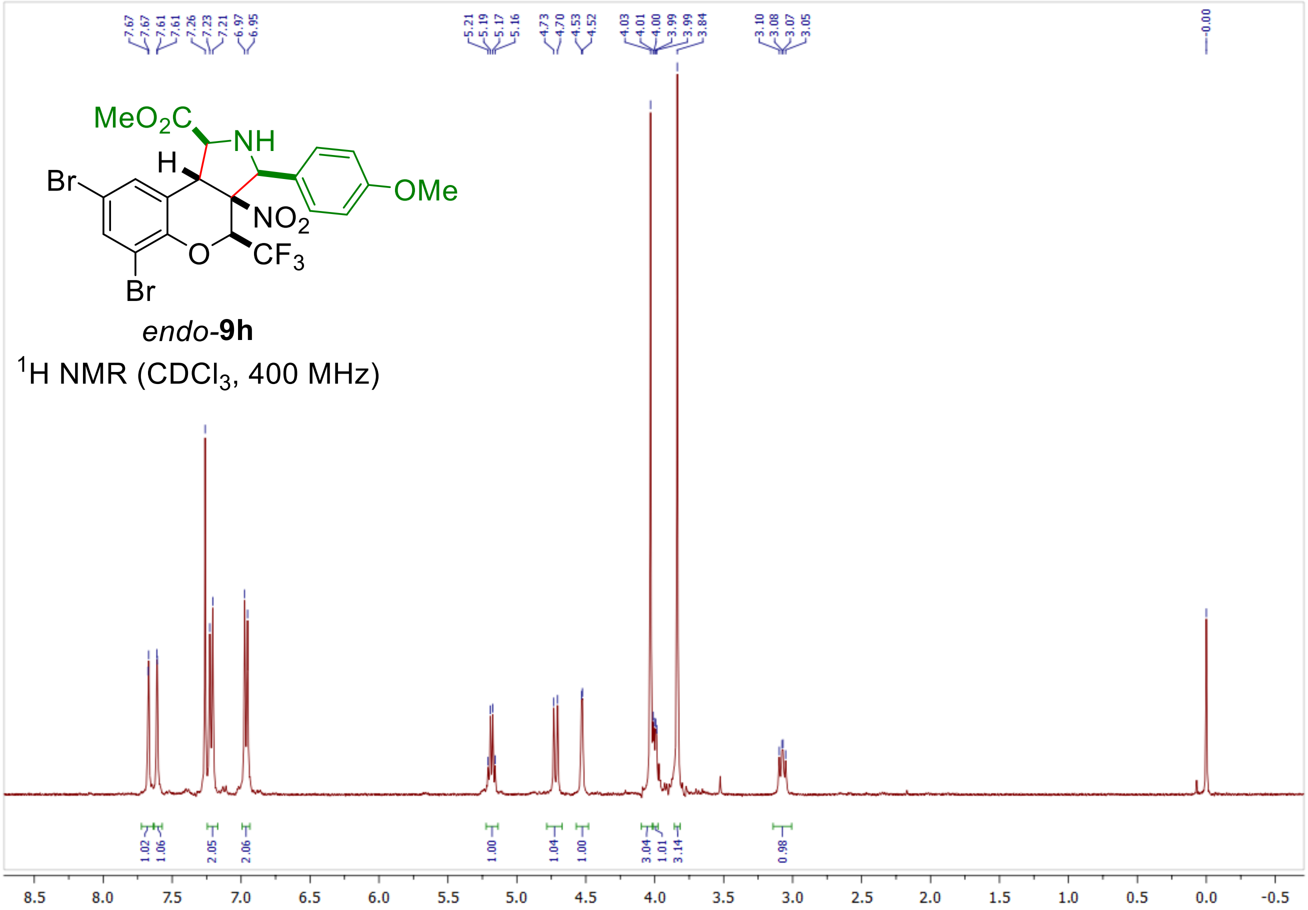

MeO2C
NH
H
Br
OMe
NO2
O
CF3
Br
endo-9h
1H NMR (CDCl3, 400 MHz)
7.67 7.67 7.61 7.61 7.26 7.23 7.21 6.97 6.95
5.21 5.19 5.17 5.16 4.73 4.70 4.53 4.52
4.03 4.01 4.00 3.99 3.99 3.84
3.10 3.08 3.07 3.05
0.00
1.02 1.06 2.05 2.06 1.00 1.04 1.00 3.04 1.01 3.14 0.98
8.5 8.0 7.5 7.0 6.5 6.0 5.5 5.0 4.5 4.0 3.5 3.0 2.5 2.0 1.5 1.0 0.5 0.0 -0.5

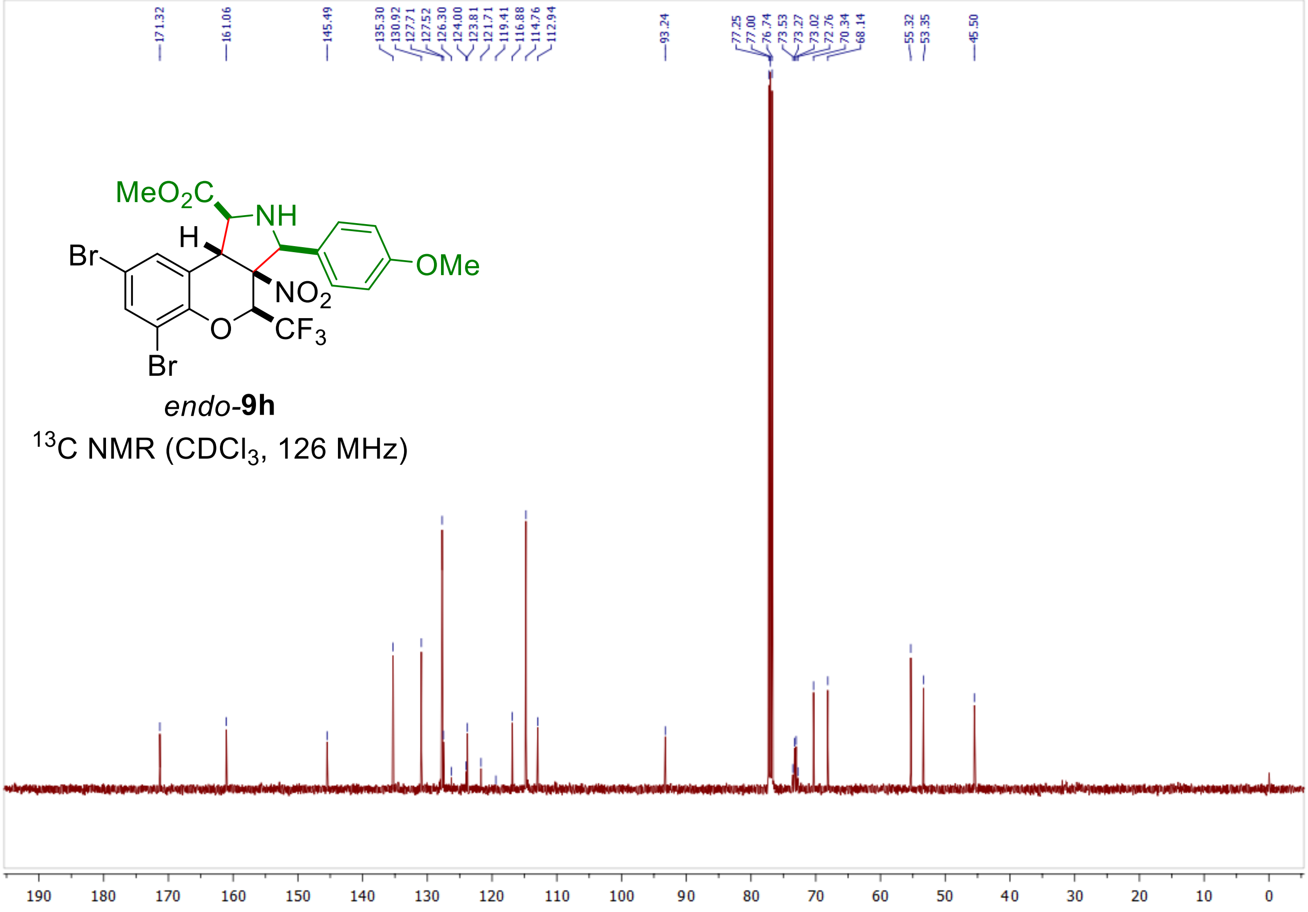

171.32
161.06
145.49
135.30 130.92 127.71 127.52 126.30 124.00 123.81 121.71 119.41 116.88 114.76 112.94
93.24
77.25 77.00 76.74 73.53 73.27 73.02 72.76 70.34 68.14
55.32 53.35
45.50
MeO2C
NH
H
Br
OMe
NO2
O
CF3
Br
endo-9h
13C NMR (CDCl3, 126 MHz)
190 180 170 160 150 140 130 120 110 100 90 80 70 60 50 40 30 20 10 0

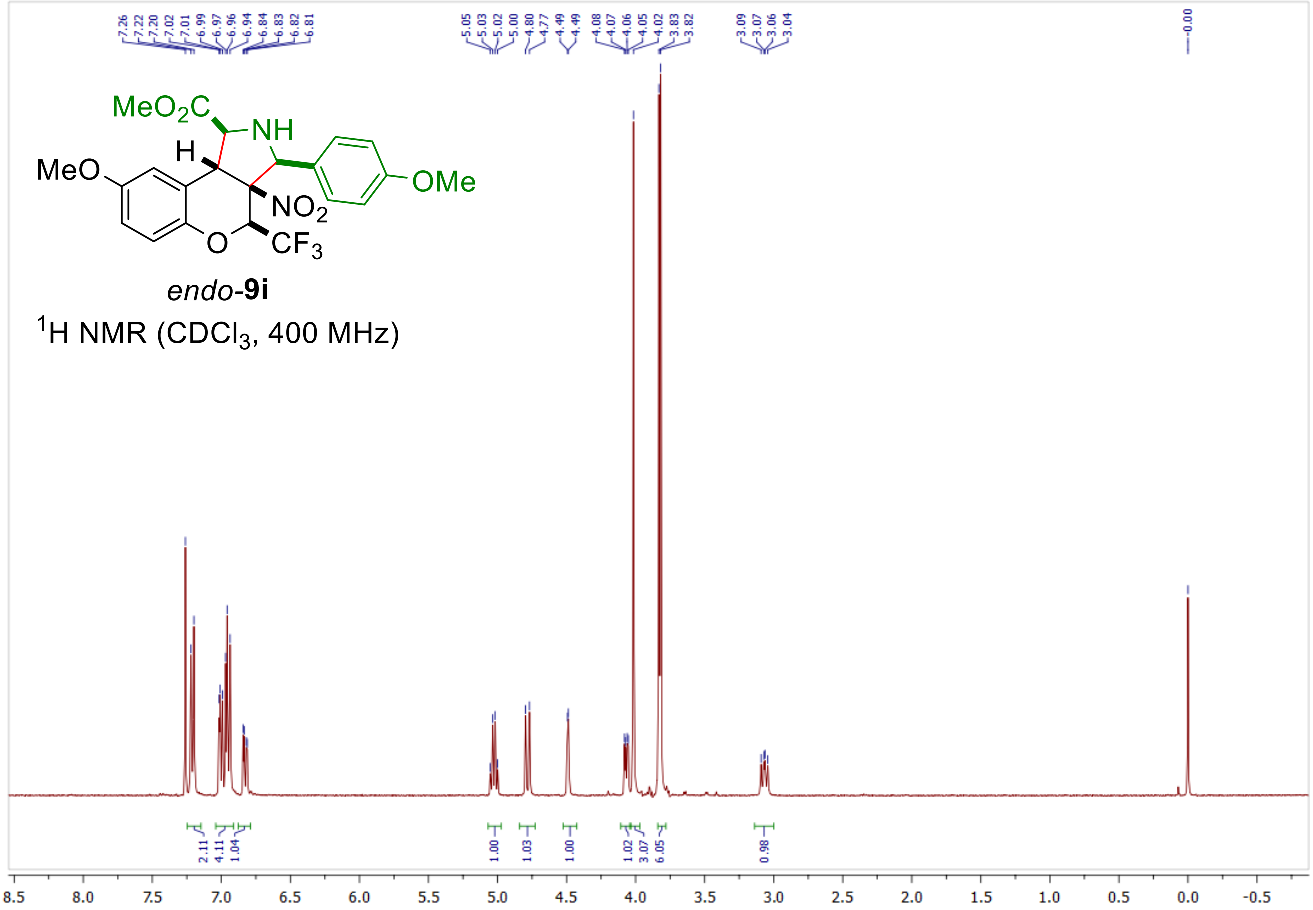
7.26 7.22 7.20 7.02 7.01 6.99 6.97 6.96 6.94 6.84 6.83 6.82 6.81
5.05 5.03 5.02 5.00 4.80 4.77 4.49 4.49 4.08 4.07 4.06 4.05 4.02 3.83 3.82
3.09 3.07 3.06 3.04
0.00
MeO$_2$C
NH
H
MeO
OMe
NO$_2$
O
CF$_3$
endo-9i
^{1}H NMR (CDCl$_3$, 400 MHz)
2.11 4.11 1.04 1.00 1.03 1.00 1.02 3.07 6.05 0.98
8.5 8.0 7.5 7.0 6.5 6.0 5.5 5.0 4.5 4.0 3.5 3.0 2.5 2.0 1.5 1.0 0.5 0.0 -0.5

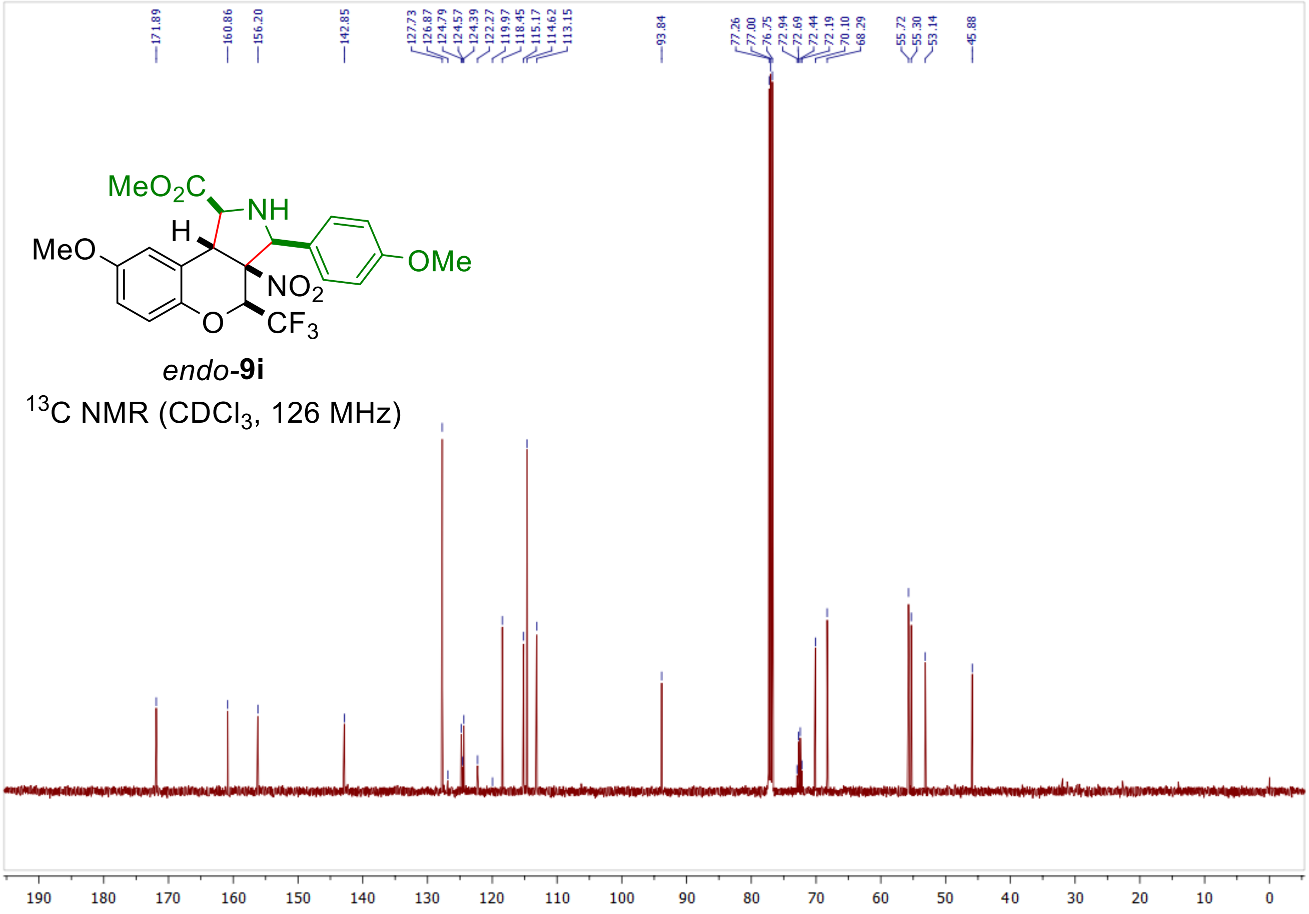
171.89
160.86 156.20
142.85
127.73 126.87 124.79 124.57 124.39 122.27 119.97 118.45 115.17 114.62 113.15
93.84
77.26 77.00 76.75 72.94 72.69 72.44 72.19 70.10 68.29
55.72 55.30 53.14
45.88
MeO$_2$C
NH
H
MeO
OMe
NO$_2$
O
CF$_3$
endo-9i
^{13}C NMR (CDCl$_3$, 126 MHz)
190 180 170 160 150 140 130 120 110 100 90 80 70 60 50 40 30 20 10 0

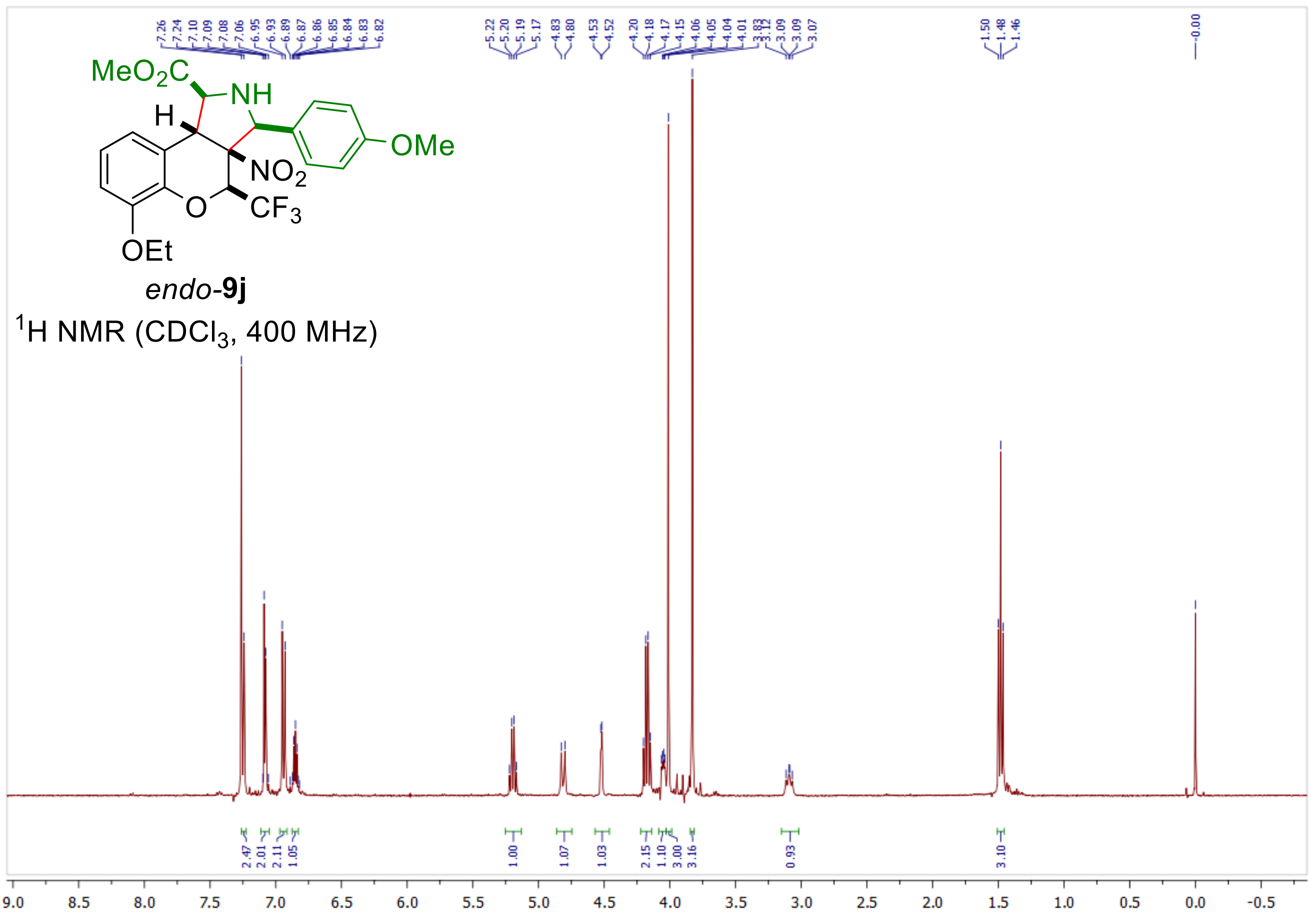
7.26 7.24 7.10 7.09 7.08 7.06 6.95 6.93 6.89 6.87 6.86 6.85 6.84 6.83 6.82
5.22 5.20 5.19 5.17 4.83 4.80 4.53 4.52 4.20 4.18 4.17 4.15 4.06 4.05 4.04 4.01 3.83 3.12 3.09 3.09 3.07
1.50 1.48 1.46
0.00
MeO2C
NH
H
OMe
NO2
O
CF3
OEt
endo-9j
1H NMR (CDCl3, 400 MHz)
2.47 2.01 2.11 1.05 1.00 1.07 1.03 2.15 1.10 3.00 3.16 0.93 3.10
9.0 8.5 8.0 7.5 7.0 6.5 6.0 5.5 5.0 4.5 4.0 3.5 3.0 2.5 2.0 1.5 1.0 0.5 0.0 -0.5

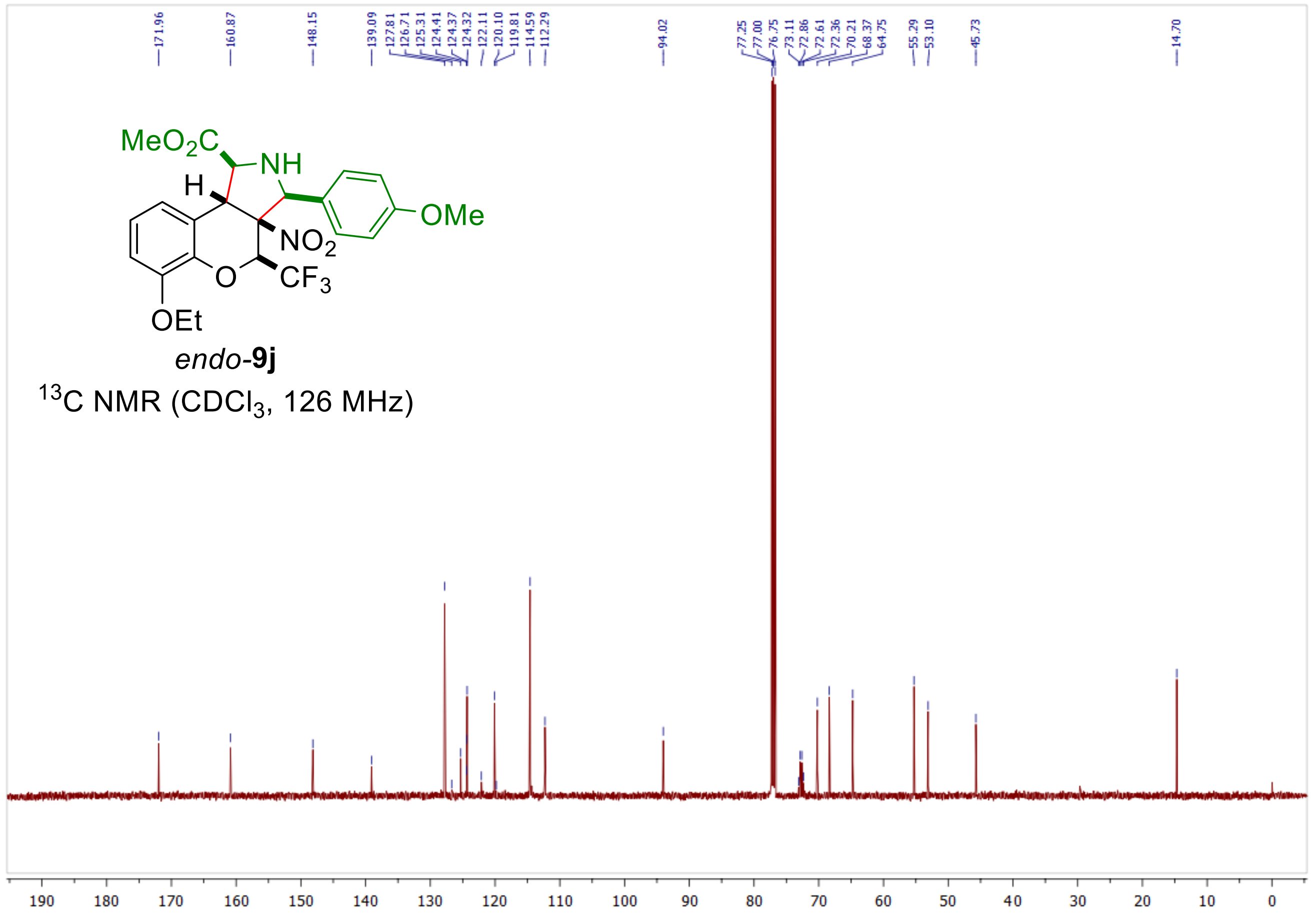
171.96
160.87
148.15
139.09 127.81 126.71 125.31 124.41 124.37 124.32 122.11 120.10 119.81 114.59 112.29
94.02
77.25 77.00 76.75 73.11 72.86 72.61 72.36 70.21 68.37 64.75
55.29 53.10
45.73
14.70
MeO2C
NH
H
OMe
NO2
O
CF3
OEt
endo-9j
13C NMR (CDCl3, 126 MHz)
190 180 170 160 150 140 130 120 110 100 90 80 70 60 50 40 30 20 10 0

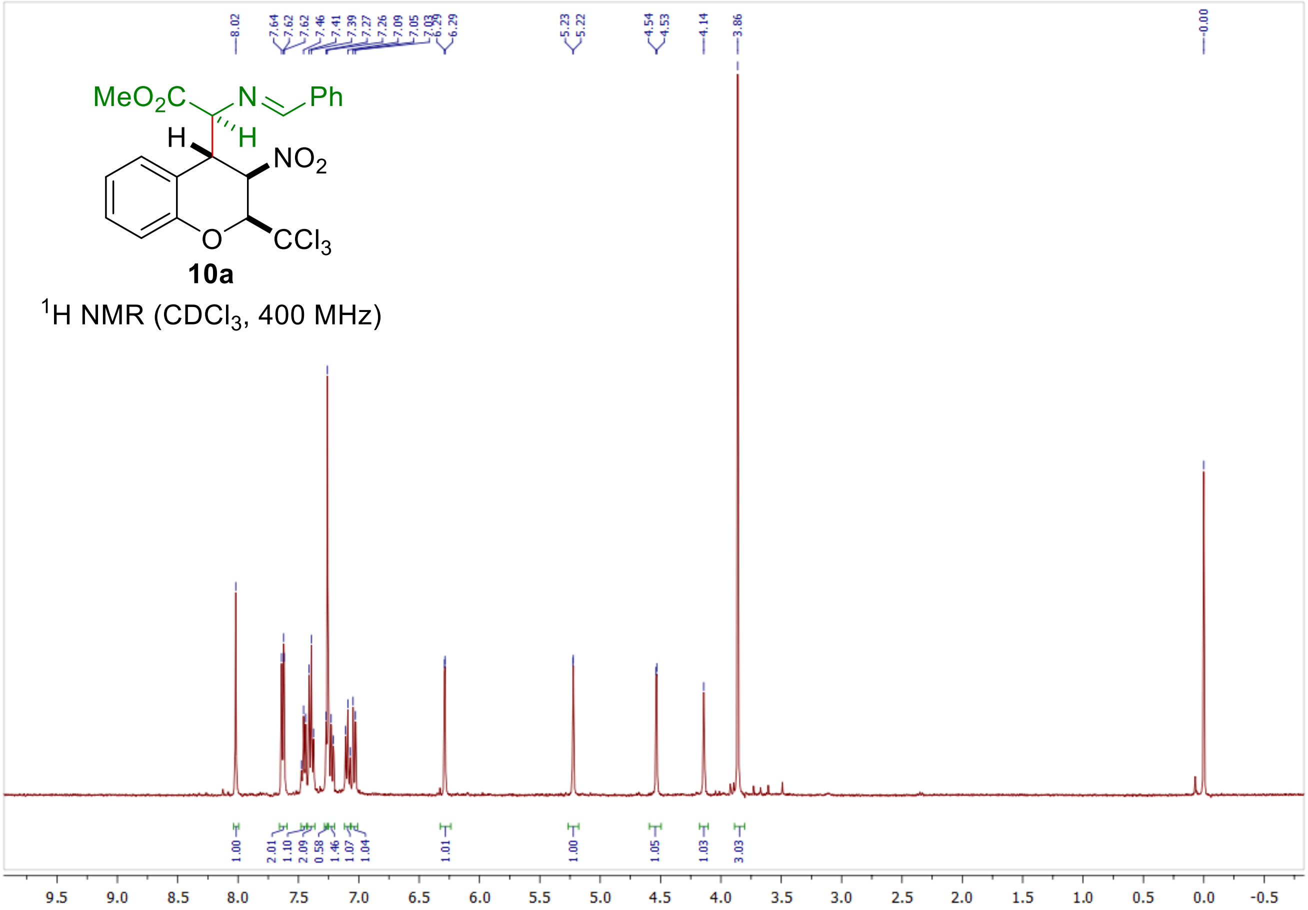
MeO2C
N
Ph
H
H
NO2
O
CCl3
10a
1H NMR (CDCl3, 400 MHz)

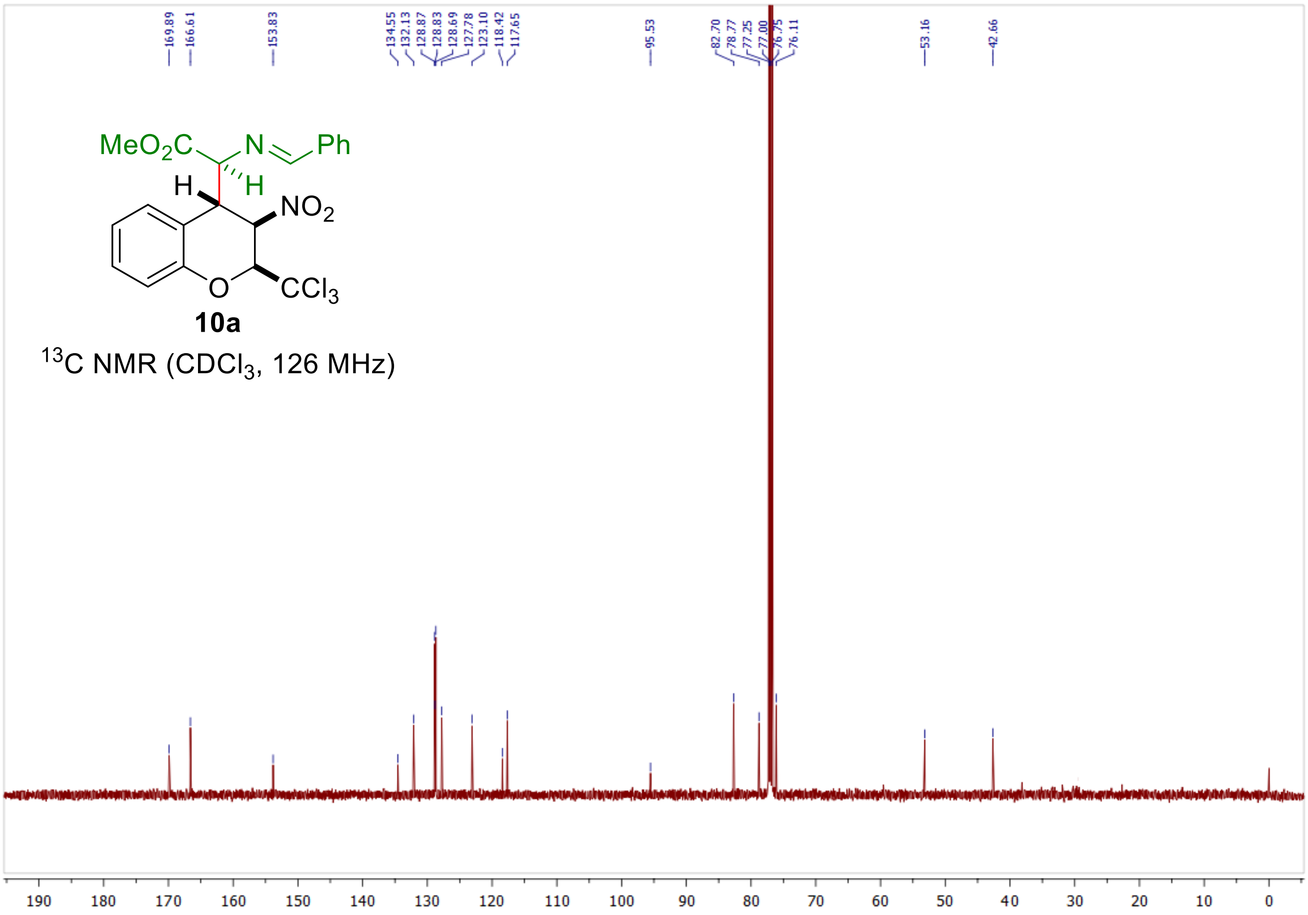
MeO2C
N
Ph
H
H
NO2
O
CCl3
10a
13C NMR (CDCl3, 126 MHz)

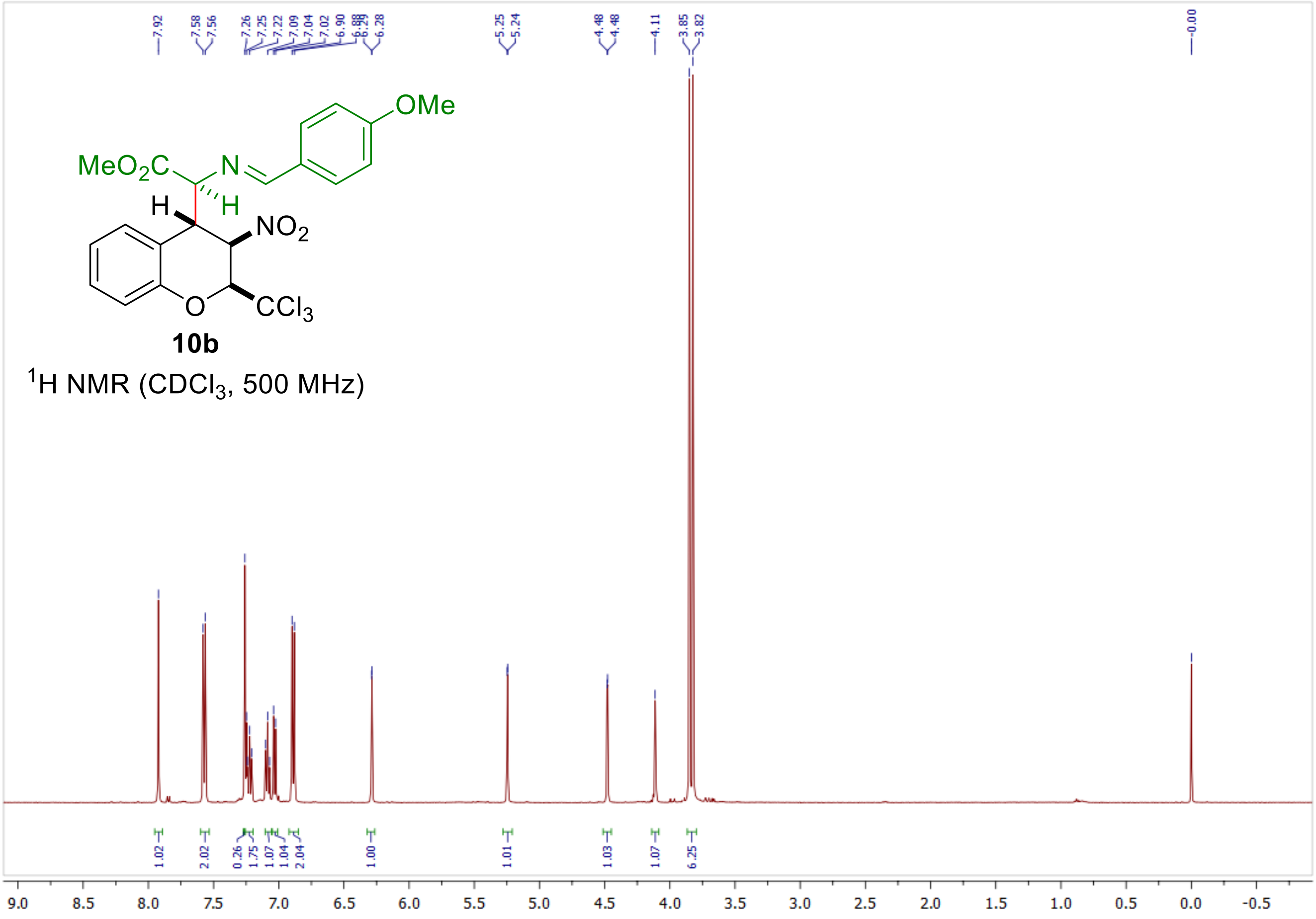
7.92
7.58
7.56
7.26
7.25
7.22
7.09
7.04
7.02
6.90
6.28
5.25
5.24
4.48
4.48
4.11
3.85
3.82
0.00
OMe
MeO2C
N
H
H
NO2
O
CCl3
10b
1H NMR (CDCl3, 500 MHz)
1.02
2.02
0.26
1.75
1.07
1.04
2.04
1.00
1.01
1.03
1.07
6.25
9.0
8.5
8.0
7.5
7.0
6.5
6.0
5.5
5.0
4.5
4.0
3.5
3.0
2.5
2.0
1.5
1.0
0.5
0.0
-0.5

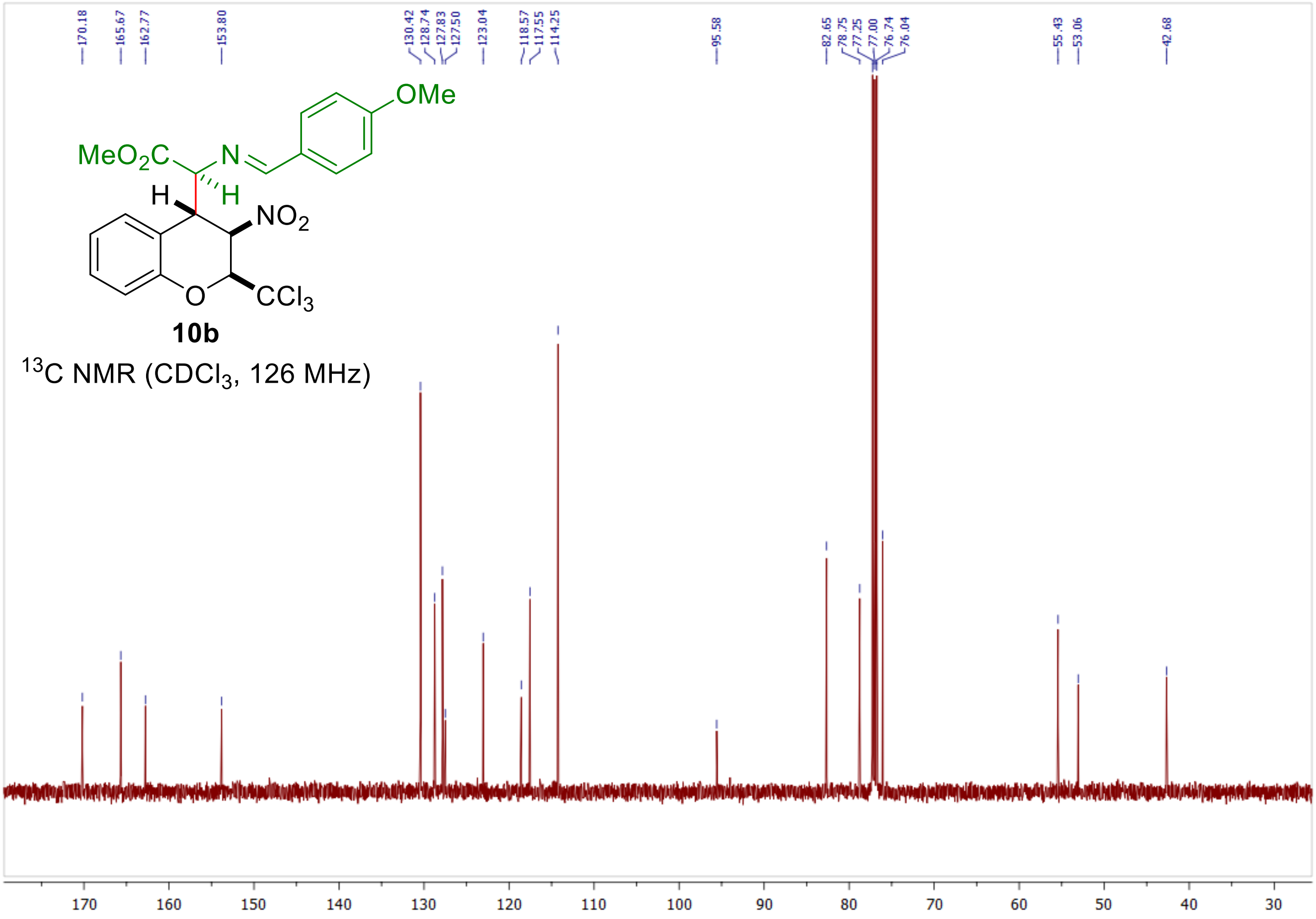
170.18
165.67
162.77
153.80
130.42
128.74
127.83
127.50
123.04
118.57
117.55
114.25
95.58
82.65
78.75
77.25
77.00
76.74
76.04
55.43
53.06
42.68
OMe
MeO2C
N
H
H
NO2
O
CCl3
10b
13C NMR (CDCl3, 126 MHz)
170
160
150
140
130
120
110
100
90
80
70
60
50
40
30

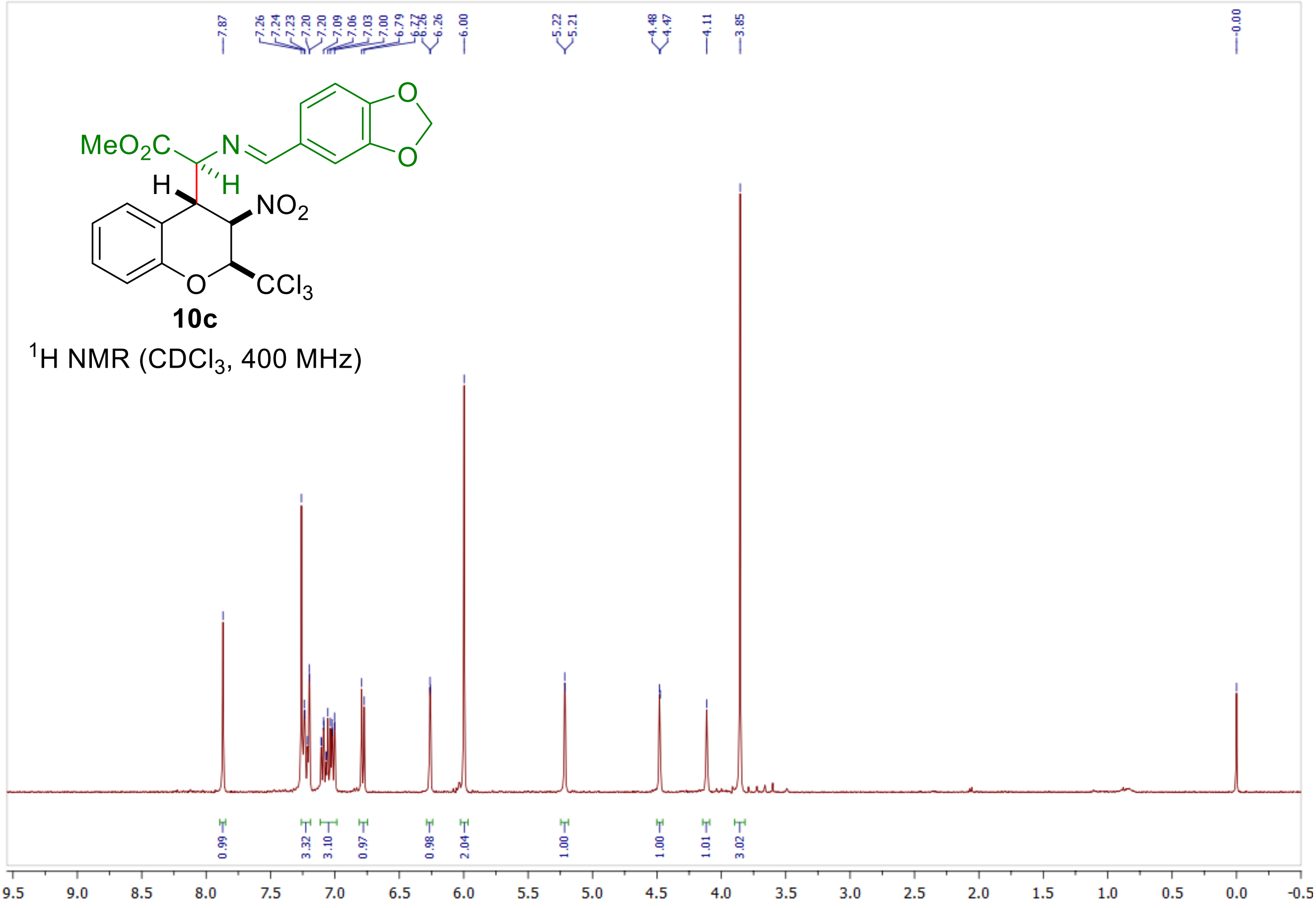

10c
^{1}H NMR (CDCl$_3$, 400 MHz)

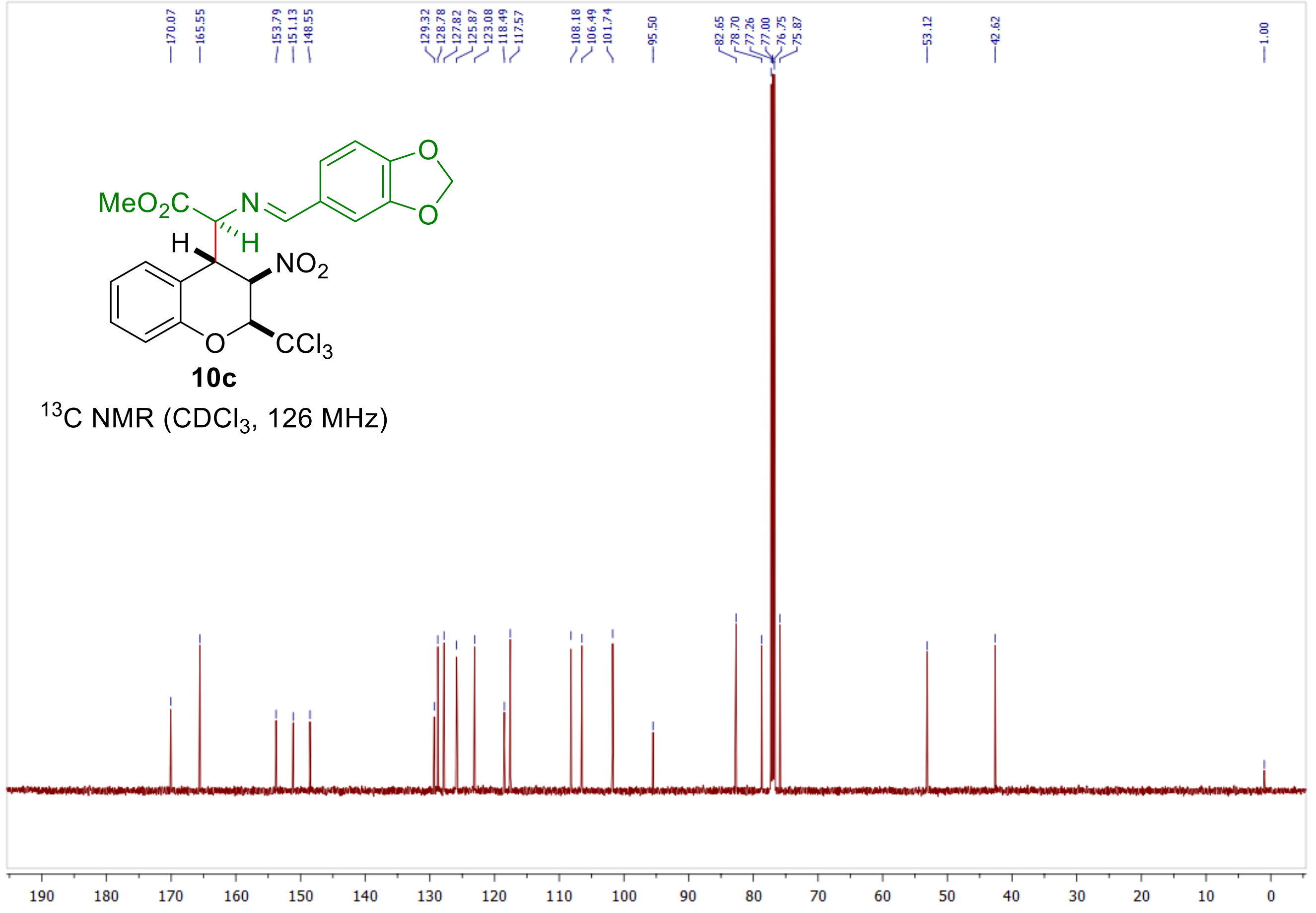

10c
^{13}C NMR (CDCl$_3$, 126 MHz)

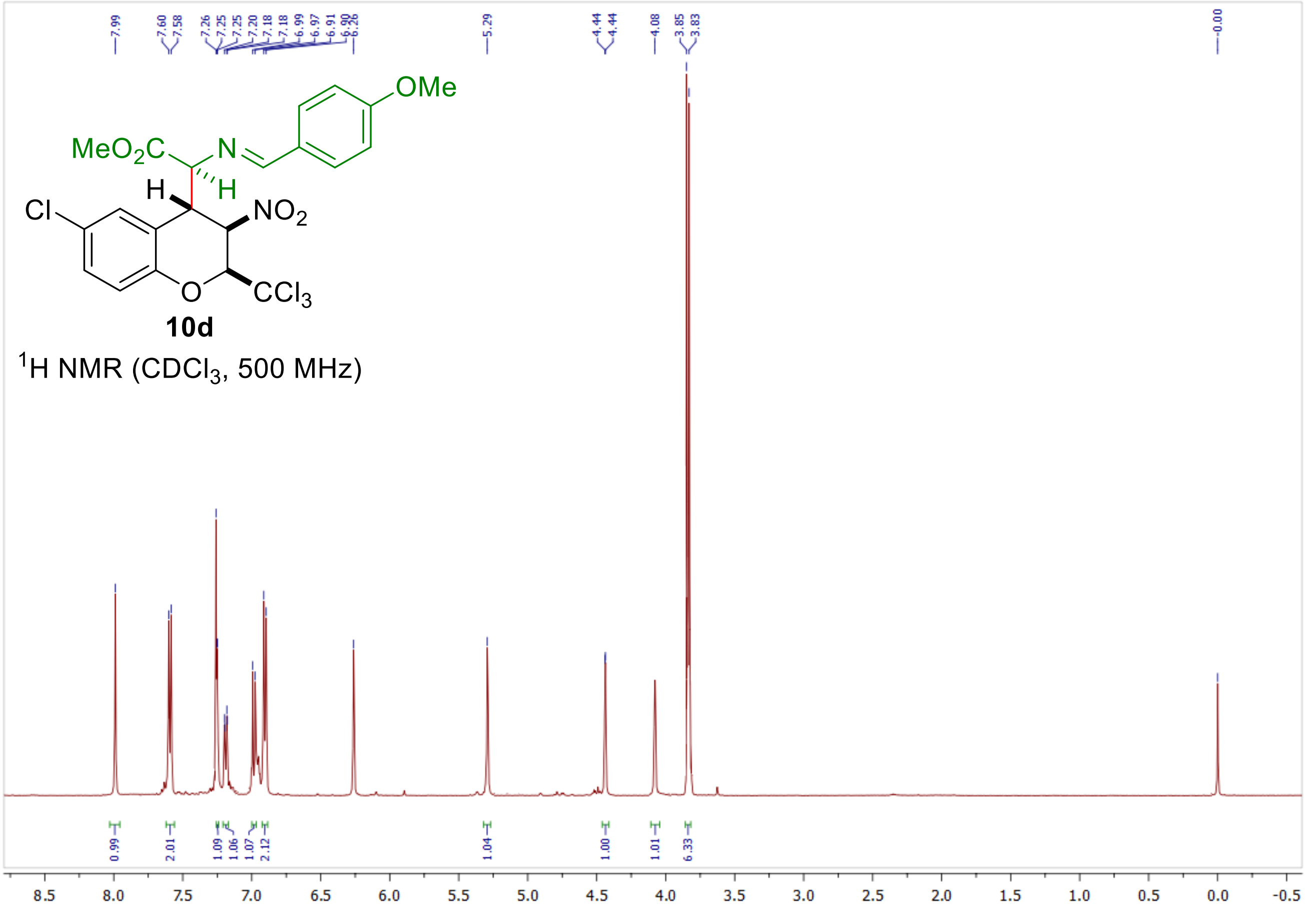
7.99
7.60
7.58
7.26
7.25
7.25
7.20
7.18
6.99
6.97
6.91
6.90
5.29
4.44
4.44
4.08
3.85
3.83
0.00
OMe
MeO2C
N
H
H
Cl
NO2
O
CCl3
10d
1H NMR (CDCl3, 500 MHz)
0.99
2.01
1.09
1.06
1.07
2.12
1.04
1.00
1.01
6.33
8.5
8.0
7.5
7.0
6.5
6.0
5.5
5.0
4.5
4.0
3.5
3.0
2.5
2.0
1.5
1.0
0.5
0.0
-0.5

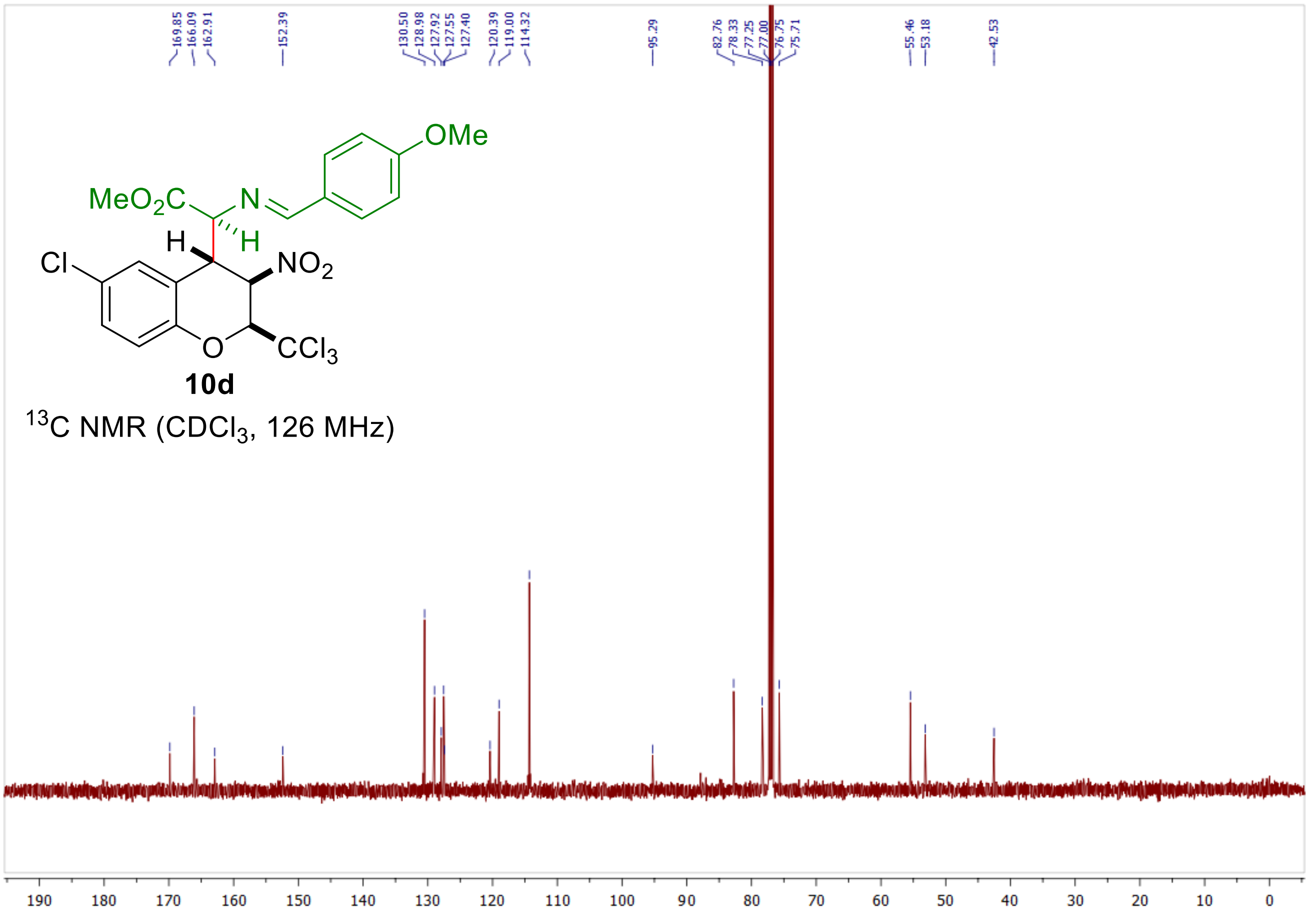
169.85
166.09
162.91
152.39
130.50
128.98
127.92
127.55
127.40
120.39
119.00
114.32
95.29
82.76
78.33
77.25
77.00
76.75
75.71
55.46
53.18
42.53
OMe
MeO2C
N
H
H
Cl
NO2
O
CCl3
10d
13C NMR (CDCl3, 126 MHz)
190
180
170
160
150
140
130
120
110
100
90
80
70
60
50
40
30
20
10
0

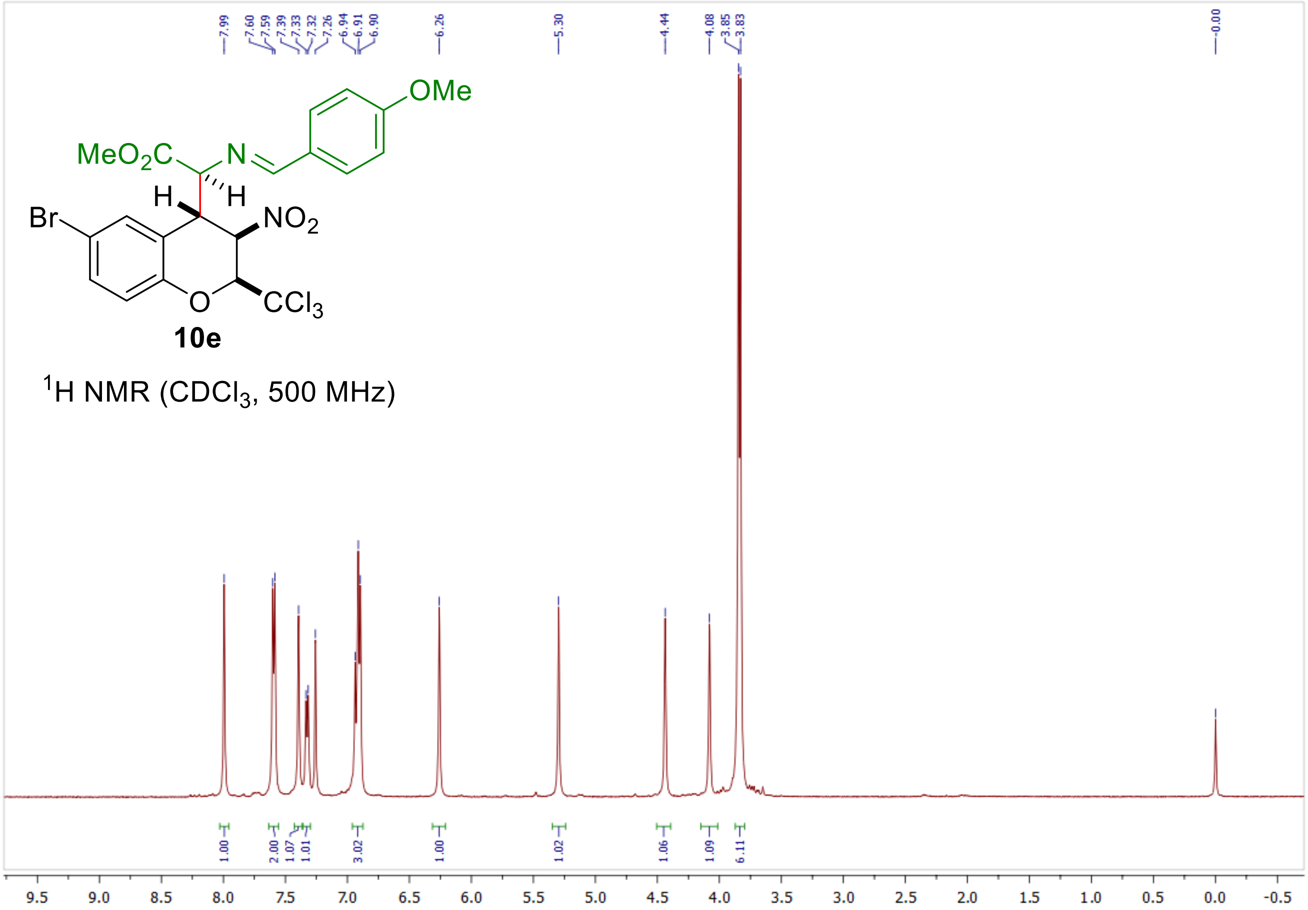
7.99
7.60
7.59
7.39
7.33
7.32
7.26
6.94
6.91
6.90
6.26
5.30
4.44
4.08
3.85
3.83
0.00
OMe
MeO2C
N
H
H
Br
NO2
O
CCl3
10e
1H NMR (CDCl3, 500 MHz)
1.00
2.00
1.07
1.01
3.02
1.00
1.02
1.06
1.09
6.11
9.5
9.0
8.5
8.0
7.5
7.0
6.5
6.0
5.5
5.0
4.5
4.0
3.5
3.0
2.5
2.0
1.5
1.0
0.5
0.0
-0.5

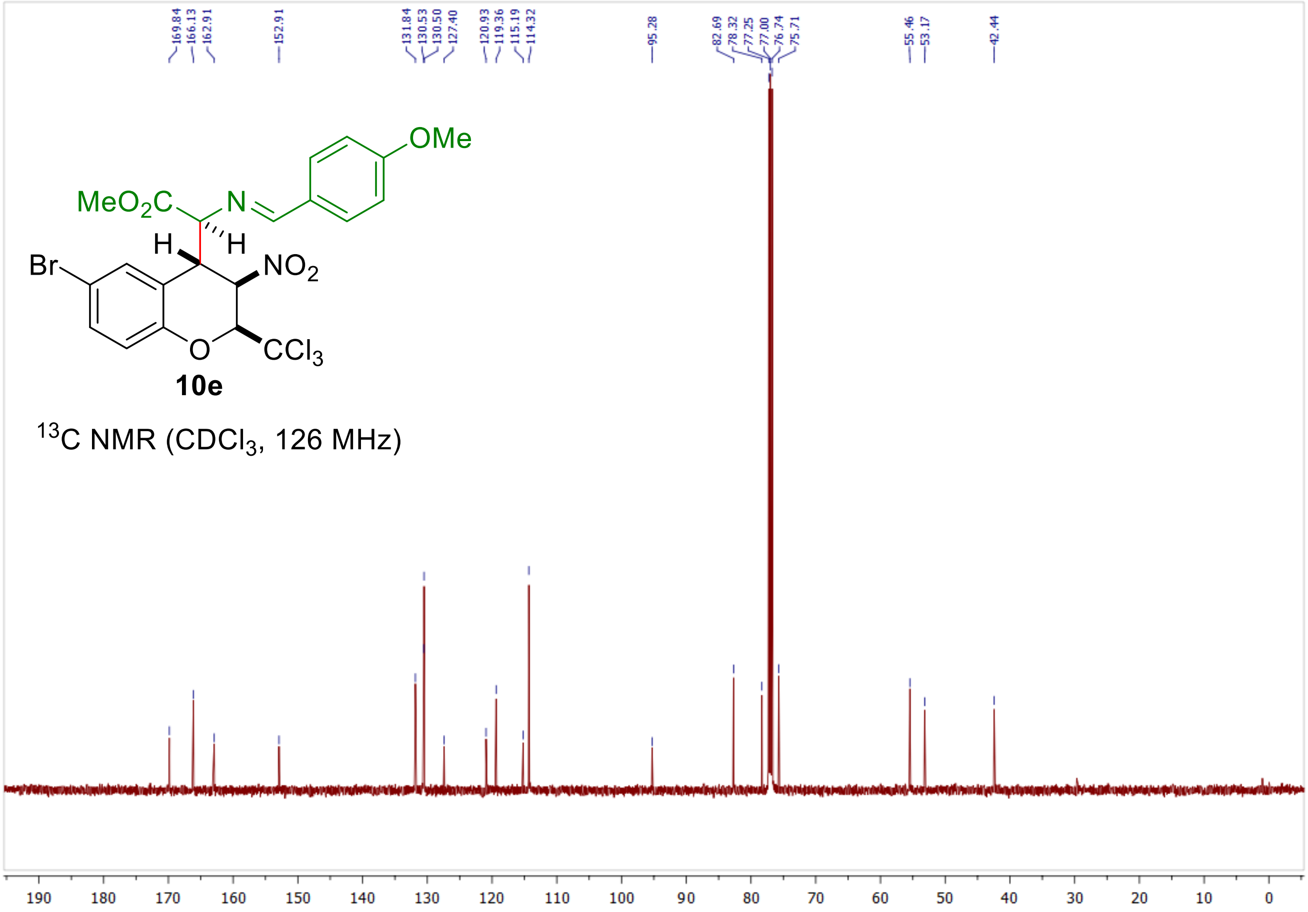
169.84
166.13
162.91
152.91
131.84
130.53
130.50
127.40
120.93
119.36
115.19
114.32
95.28
82.69
78.32
77.25
77.00
76.74
75.71
55.46
53.17
42.44
OMe
MeO2C
N
H
H
Br
NO2
O
CCl3
10e
13C NMR (CDCl3, 126 MHz)
190
180
170
160
150
140
130
120
110
100
90
80
70
60
50
40
30
20
10
0

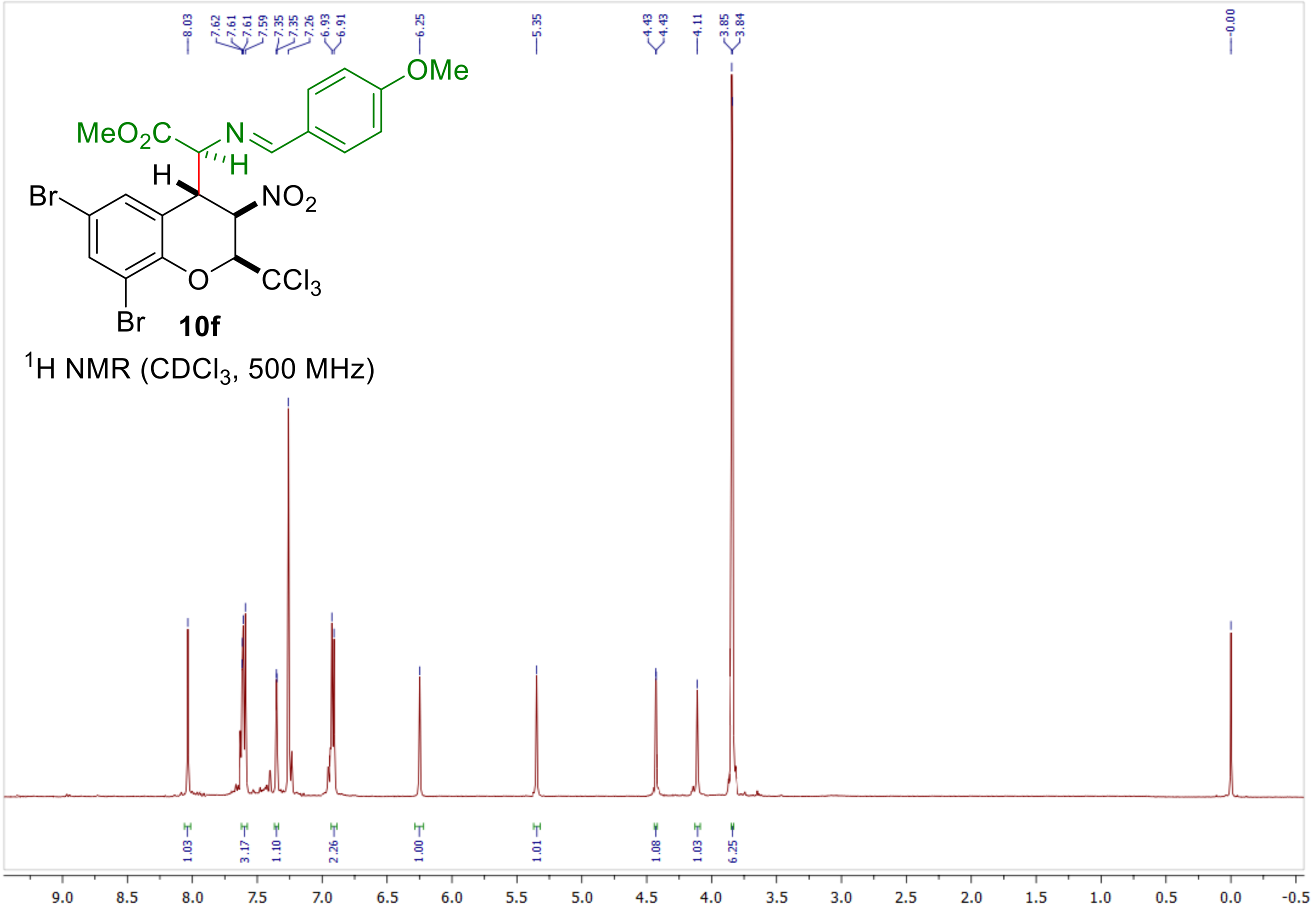
8.03
7.62
7.61
7.61
7.59
7.35
7.35
7.26
6.93
6.91
6.25
5.35
4.43
4.43
4.11
3.85
3.84
0.00
MeO2C
N
OMe
H
H
Br
NO2
O
CCl3
Br
10f
1H NMR (CDCl3, 500 MHz)
1.03
3.17
1.10
2.26
1.00
1.01
1.08
1.03
6.25
9.0
8.5
8.0
7.5
7.0
6.5
6.0
5.5
5.0
4.5
4.0
3.5
3.0
2.5
2.0
1.5
1.0
0.5
0.0
-0.5

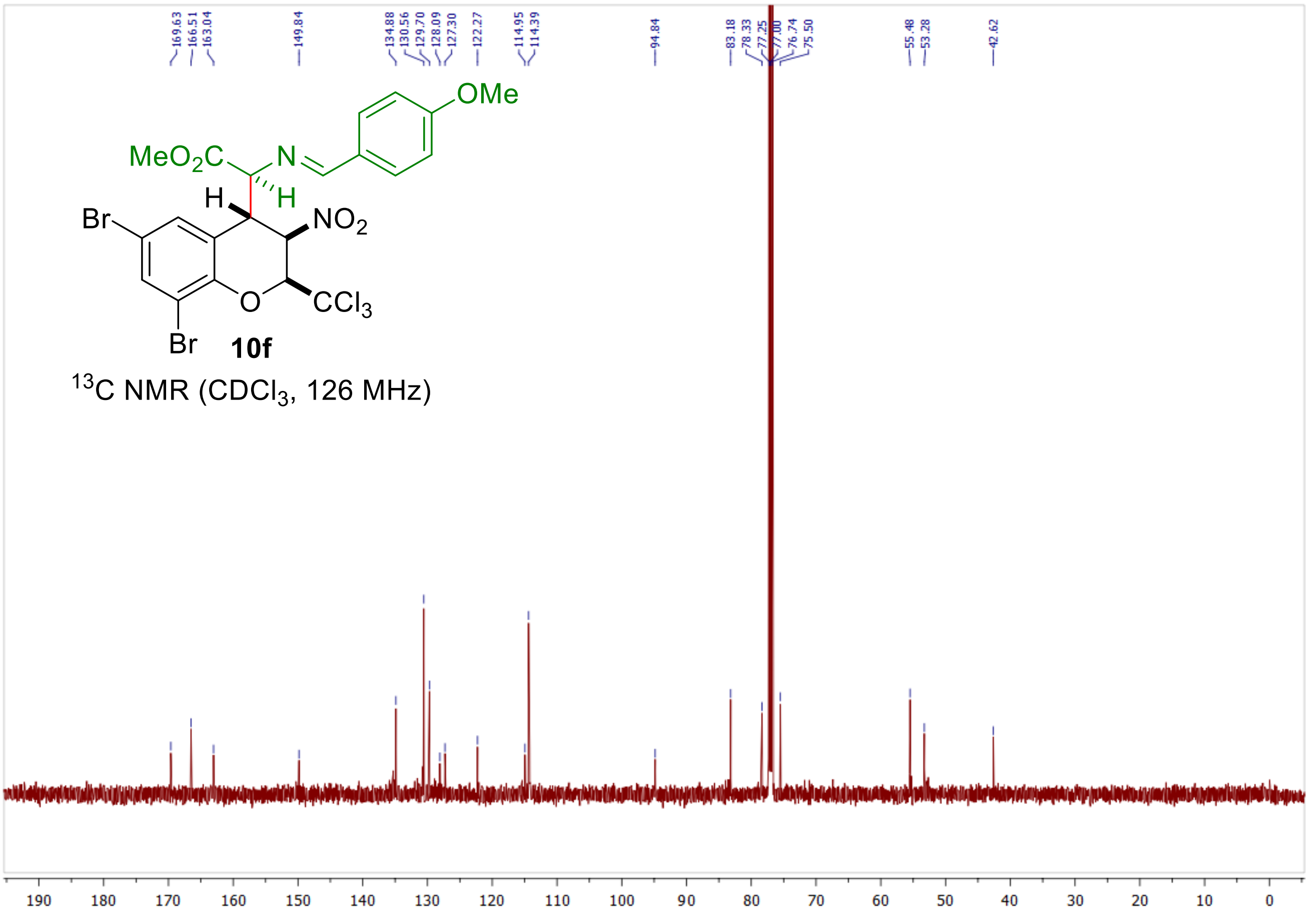
169.63
166.51
163.04
149.84
134.88
130.56
129.70
128.09
127.30
122.27
114.95
114.39
94.84
83.18
78.33
77.25
77.00
76.74
75.50
55.48
53.28
42.62
MeO2C
N
OMe
H
H
Br
NO2
O
CCl3
Br
10f
13C NMR (CDCl3, 126 MHz)
190
180
170
160
150
140
130
120
110
100
90
80
70
60
50
40
30
20
10
0

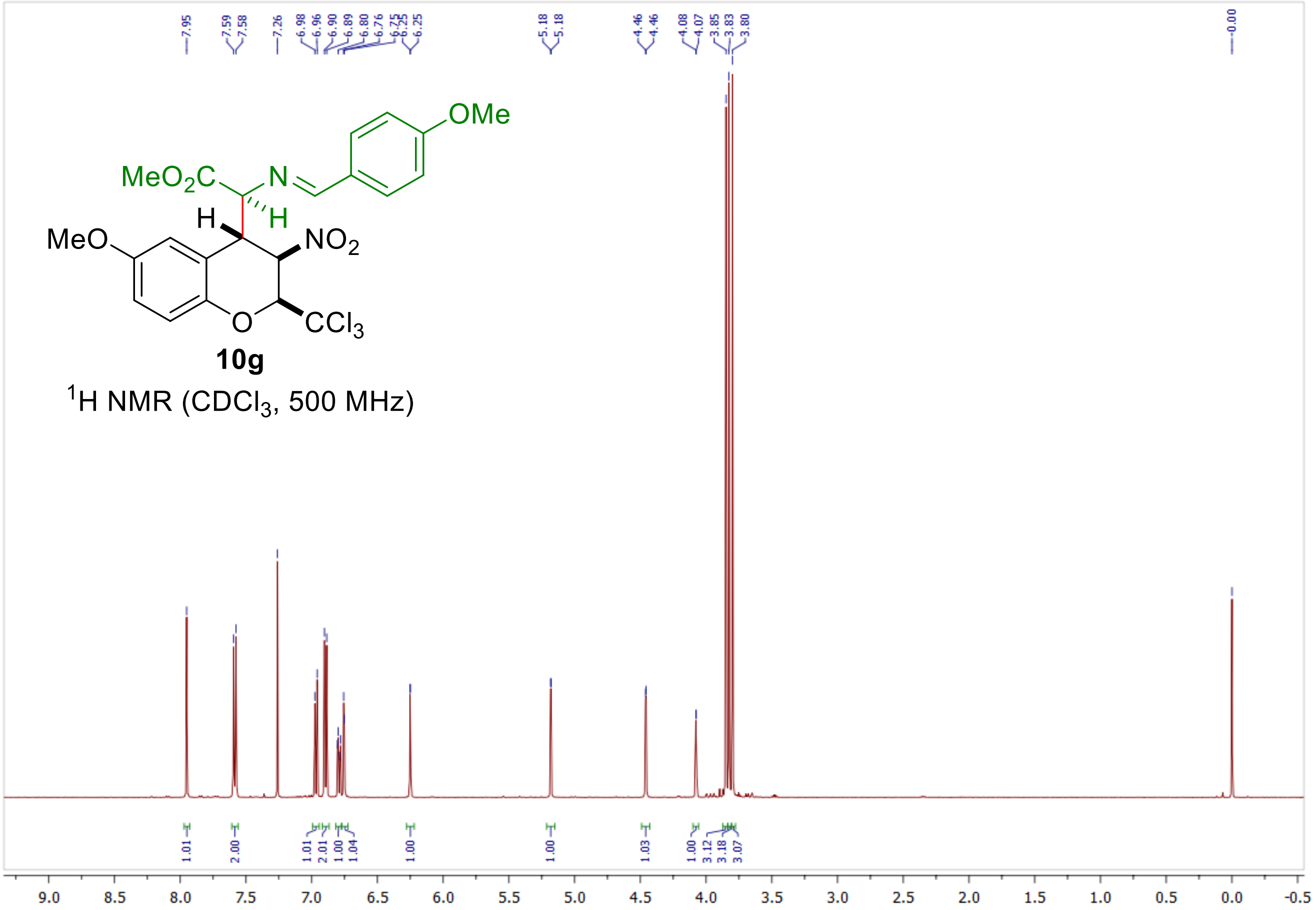

10g

^{1}H NMR ($CDCl_3$, 500 MHz)

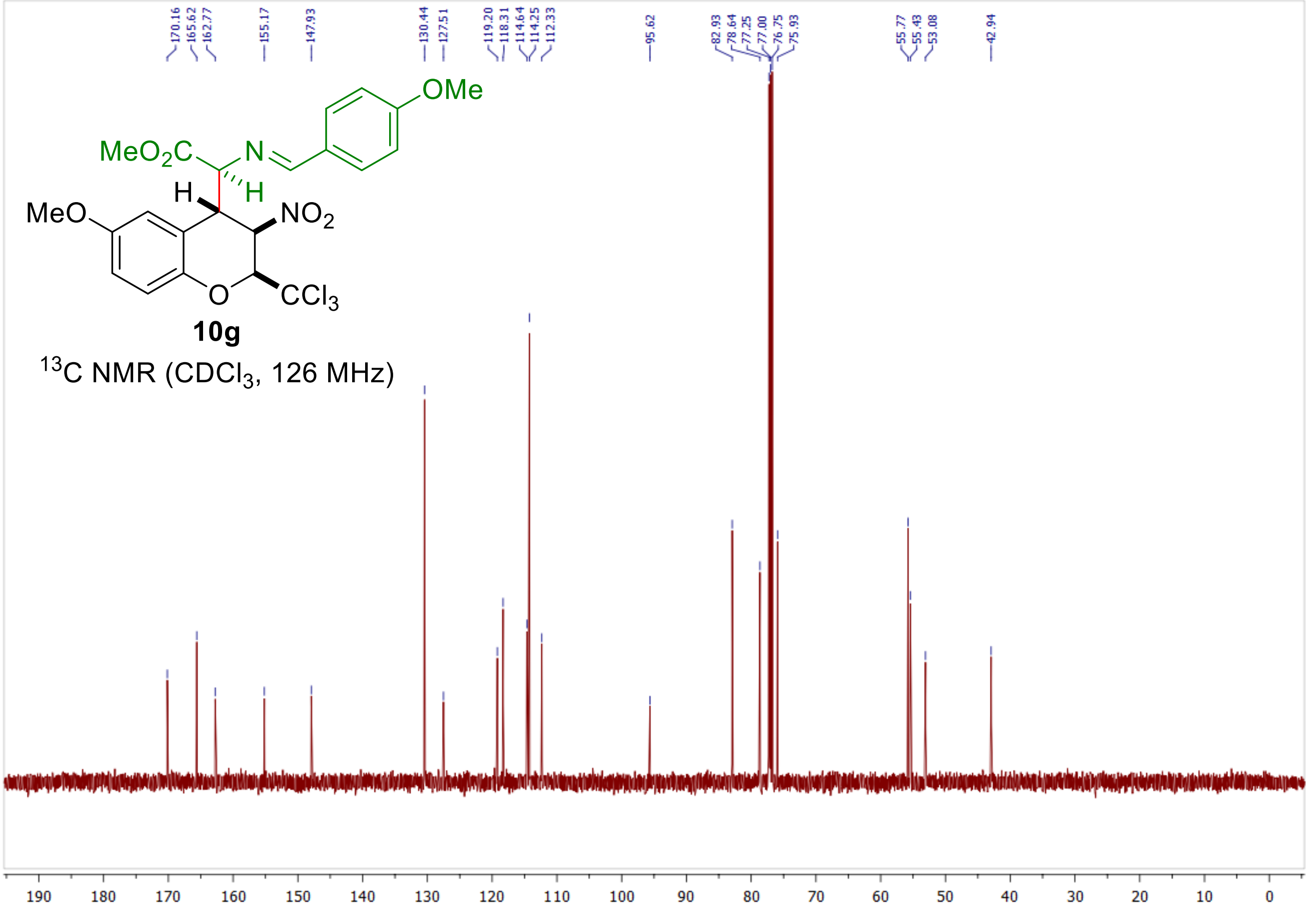

10g

^{13}C NMR ($CDCl_3$, 126 MHz)

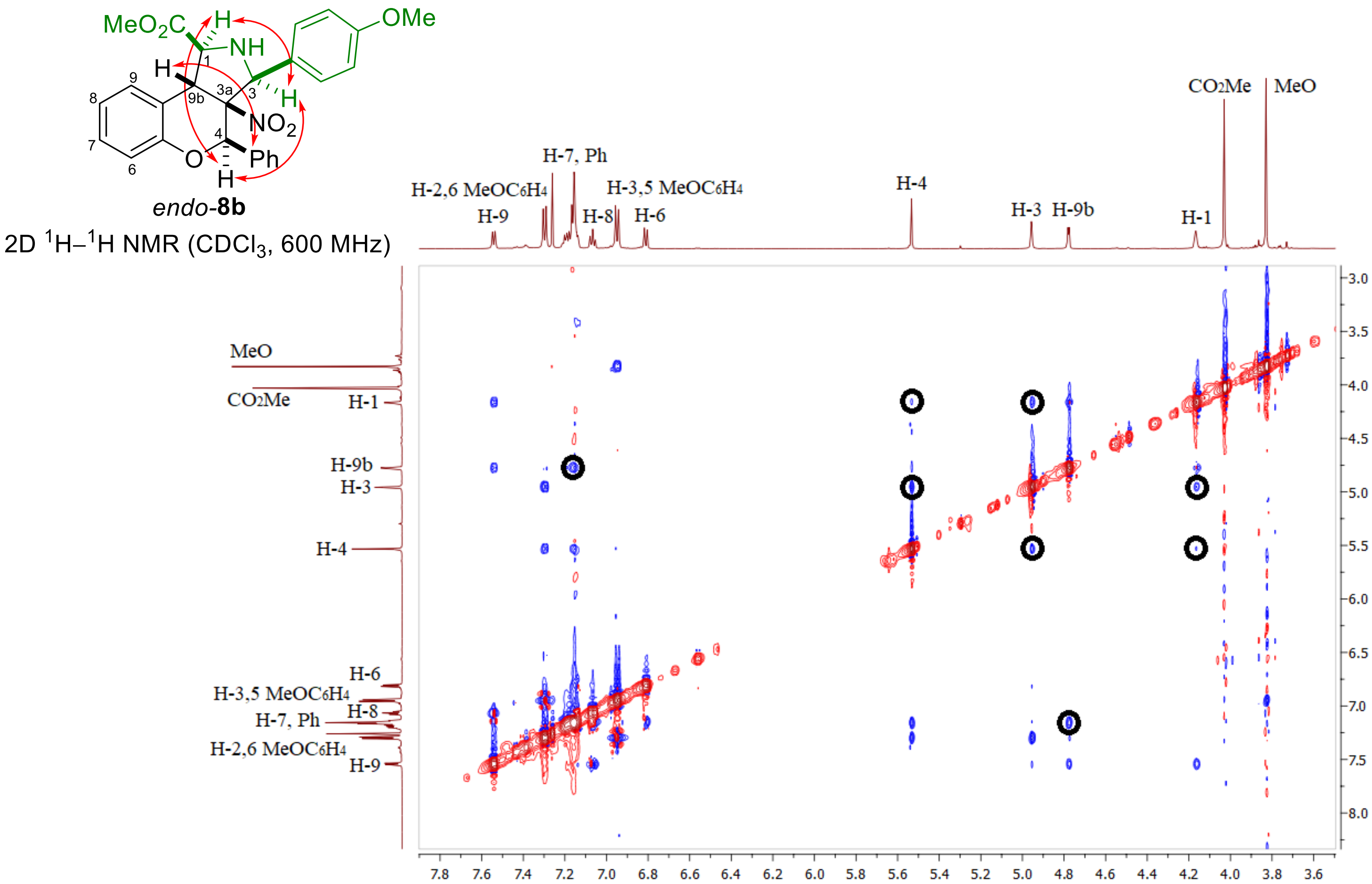
MeO2C
NH
OMe
NO2
Ph
endo-8b
2D 1H–1H NMR (CDCl3, 600 MHz)
CO2Me
MeO
H-7, Ph
H-2,6 MeOC6H4
H-3,5 MeOC6H4
H-4
H-3
H-9b
H-9
H-8
H-6
H-1

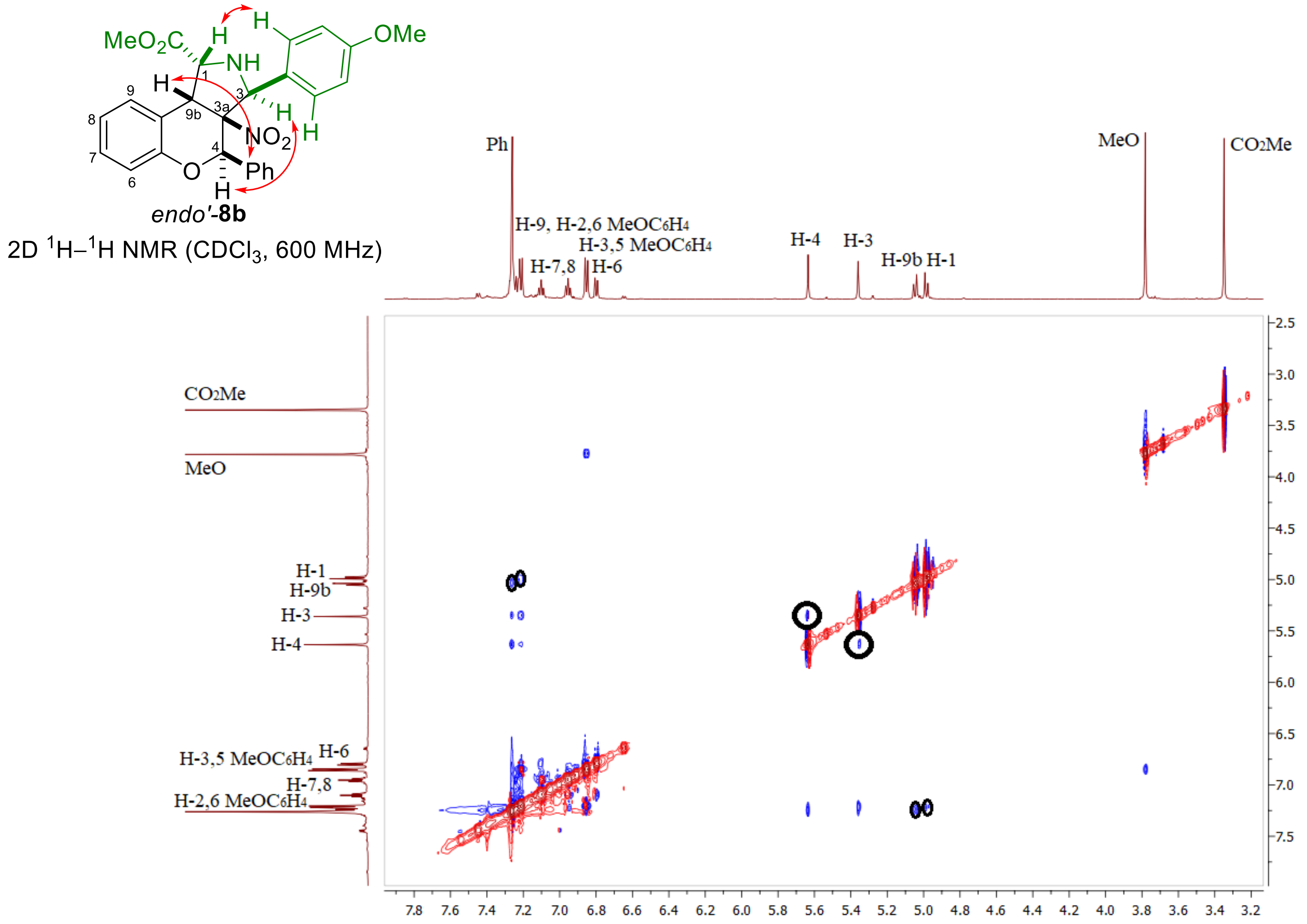
MeO2C
NH
OMe
NO2
Ph
endo'-8b
2D 1H–1H NMR (CDCl3, 600 MHz)
Ph
MeO
CO2Me
H-9, H-2,6 MeOC6H4
H-3,5 MeOC6H4
H-7,8
H-6
H-4
H-3
H-9b
H-1

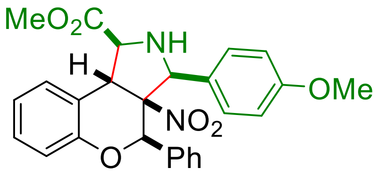

endo-**8b**

2D ^{1}H–^{13}C HSQC NMR

($CDCl_3$, 600 MHz)

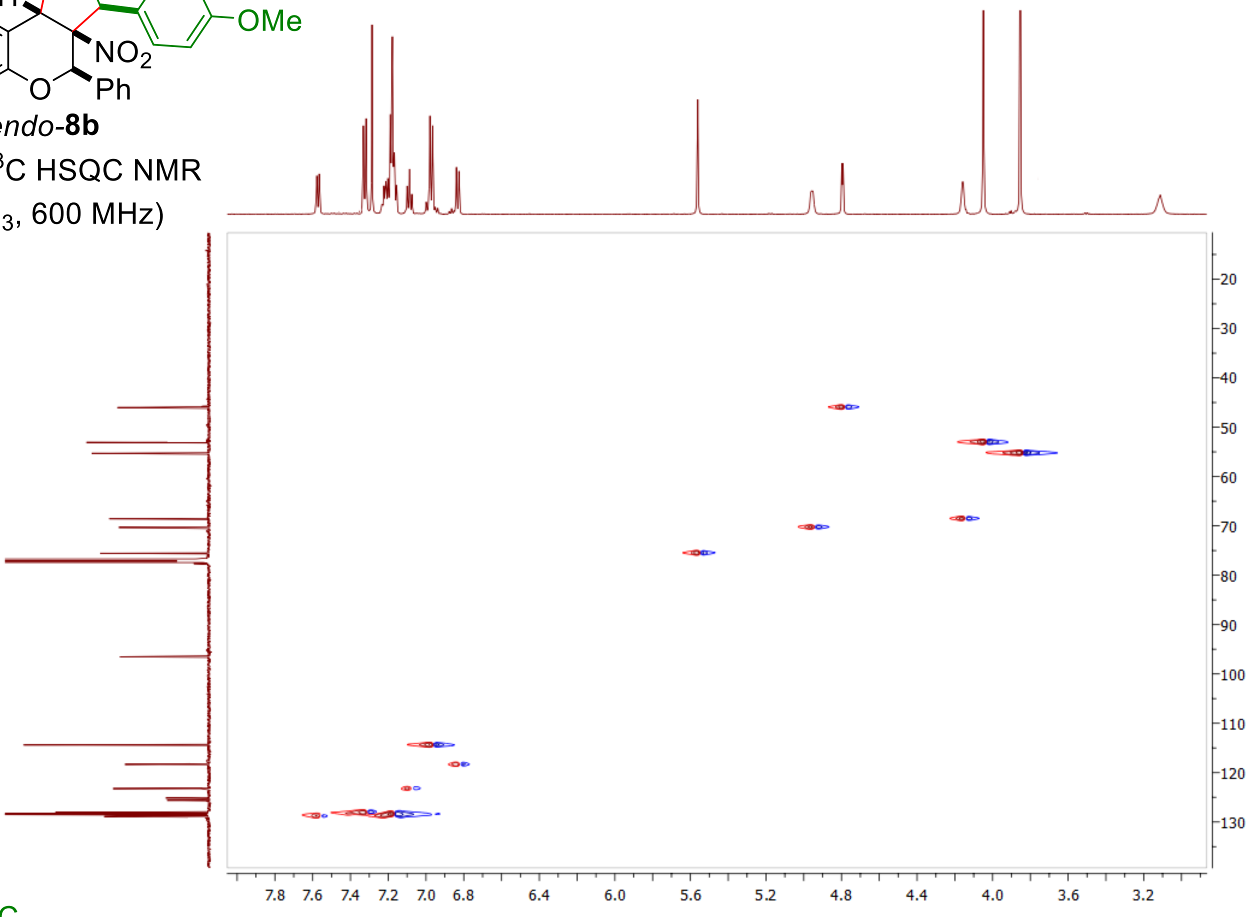

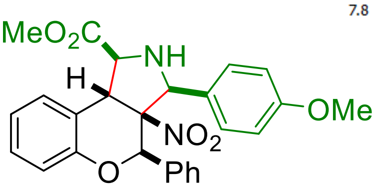

endo-**8b**

2D ^{1}H–^{13}C HMBC NMR

($CDCl_3$, 600 MHz)

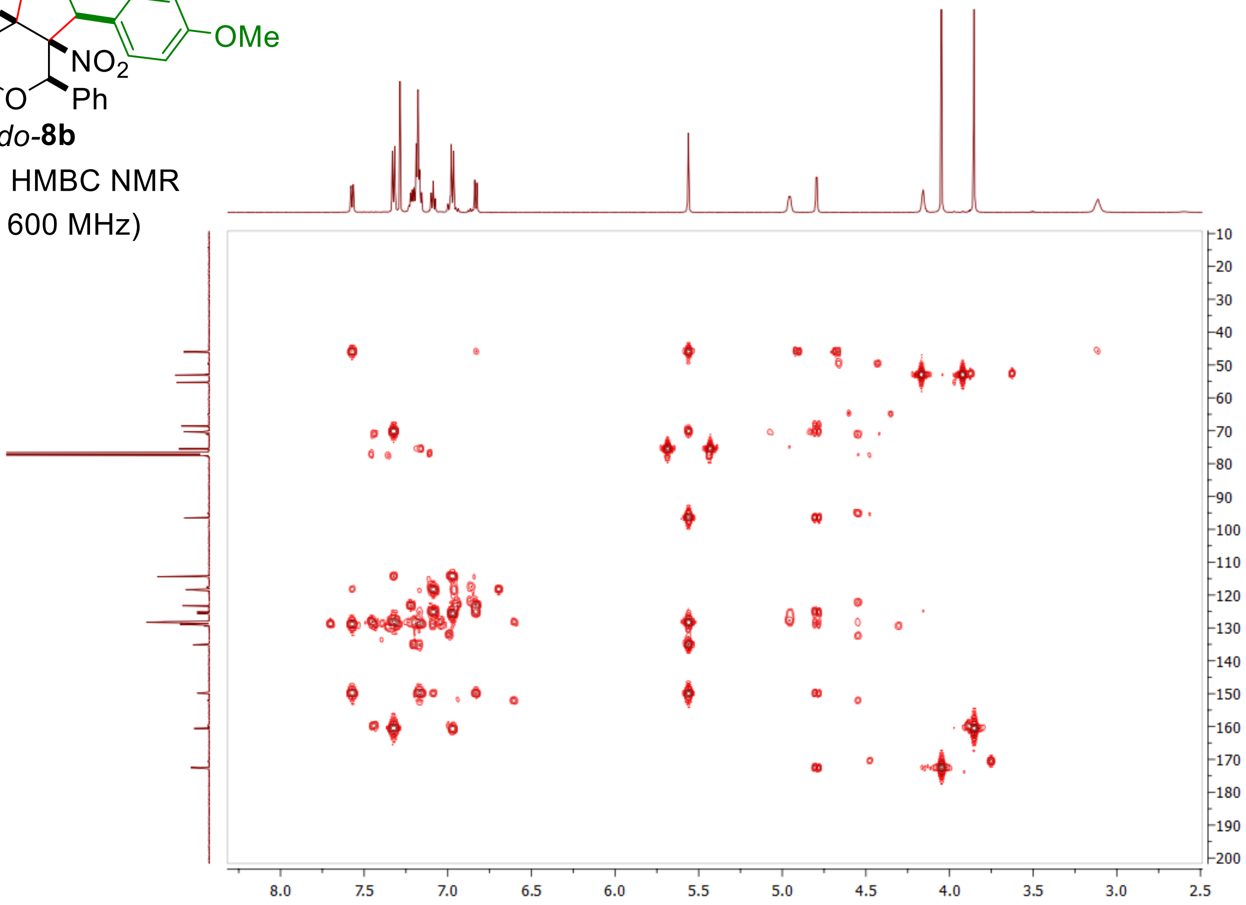

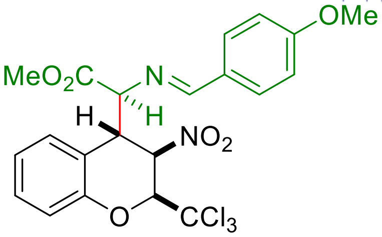

10b

^{13}C APT NMR ($CDCl_3$, 400 MHz)

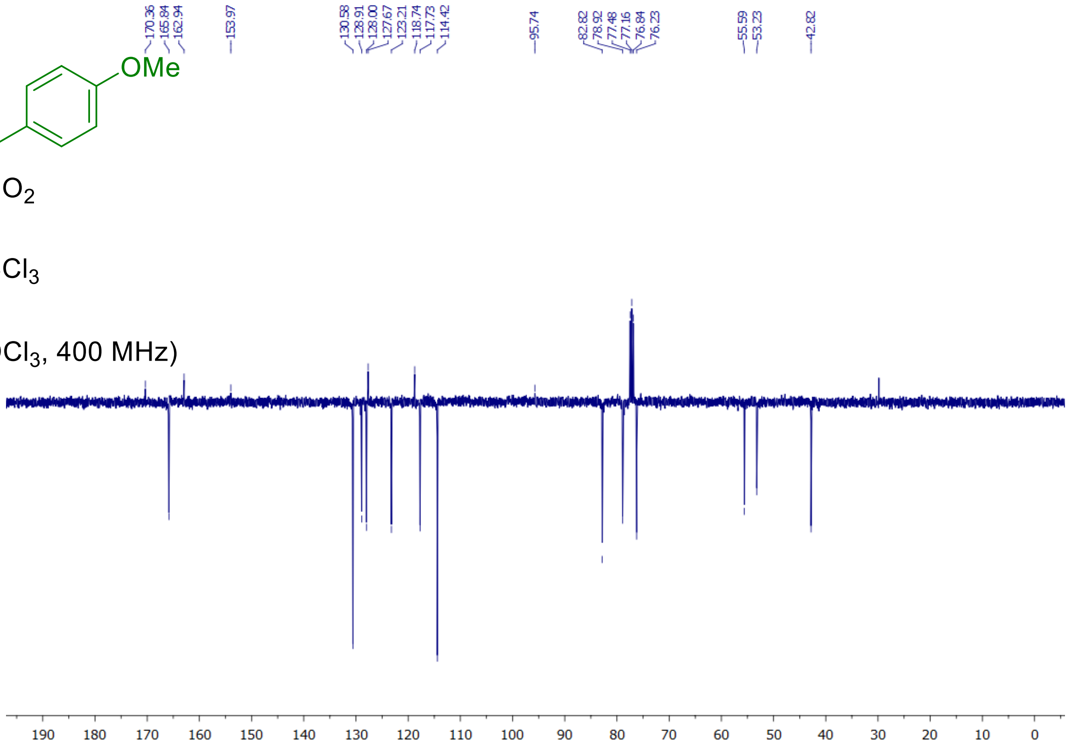

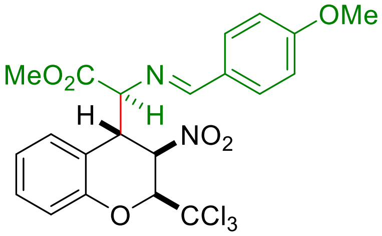

10b

2D ^{1}H–^{13}C HMBC NMR ($CDCl_3$, 400 MHz)

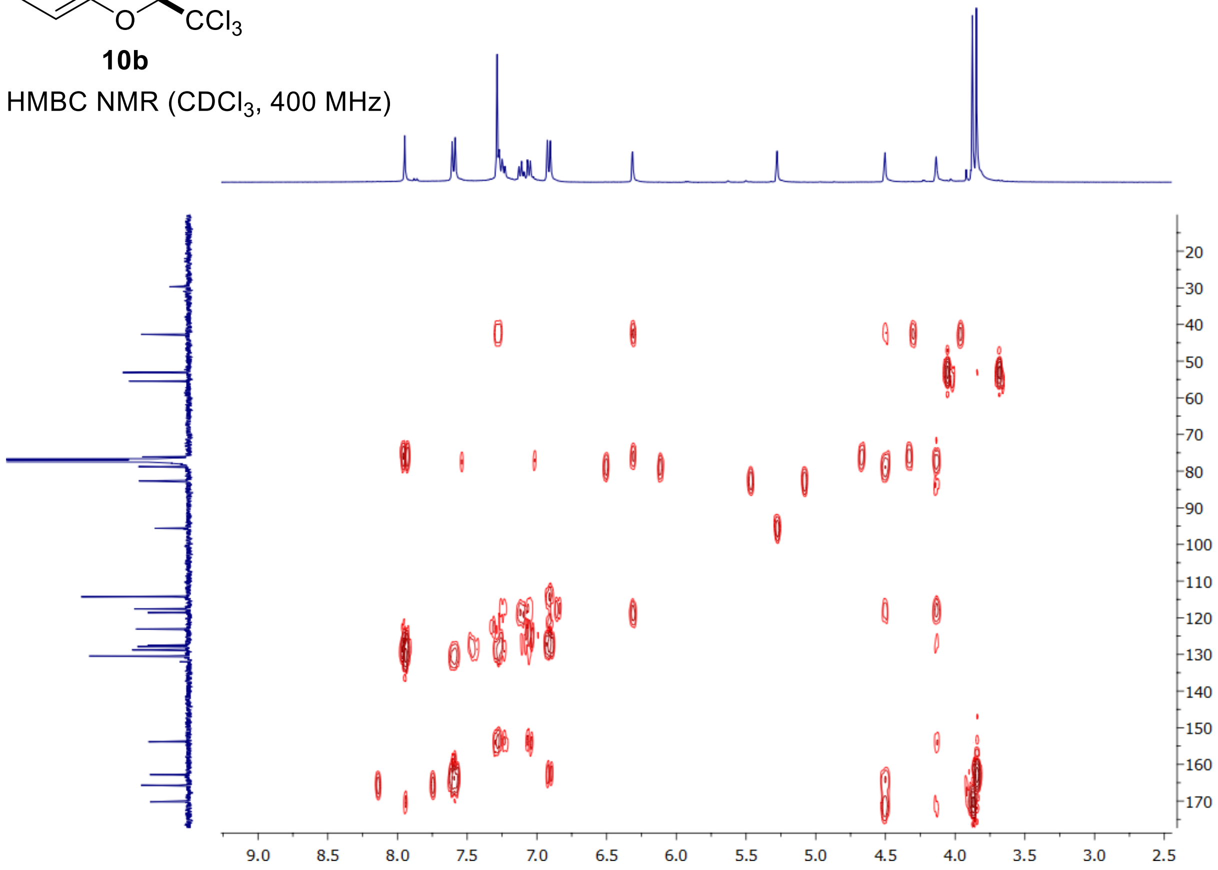

X-ray crystallography

Crystals of compounds *endo*-**8b**, *endo*-**9a** and **10c** were grown by slow evaporation of an ethyl acetate solution. Diffraction data for *endo*-**8b** and *endo*-**9a**, **10c** were collected on an Xcalibur Eos and Xcalibur 3 automatic diffractometers, respectively, using the standard procedure (Mo-Ka radiation (λ = 0.71073 Å), graphite monochromator, ω-scanning, 295(2) K). An empirical adjustment for absorption was introduced. The structures were solved by direct methods and refined by the full-matrix least-squares method using the SHELX-97 program package [1]. All non-hydrogen atoms were refined with anisotropic atomic displacement and hydrogen atoms were included at the calculated positions using a riding model. The geometrical parameters were analysed using the program OLEX2 [2].

Crystal data for *endo*-8b ($C_{26}H_{24}N_2O_6$, 460.49). Triclinic crystals, space group $P\bar{1}$, a = 9.9193(7), b = 11.0749(6) and c = 11.3940(6) Å, α = 88.132(4), β = 68.824(6) and γ = 75.357(5)°, V = 1126.86(11) Å^3, D_c = 1.357, absorption coefficient μ = 0.097 mm^{-1}, Z = 2. The intensities of 5100 independent reflections (R_{int} = 0.0185) were measured. The final discrepancy factors R_1 = 0.0492, wR_2 = 0.1034, GooF = 1.000 for 3339 reflections with $I > 2\sigma(I)$; R_1 = 0.0852, wR_2 = 0.1218 (all data). Largest different peaks and holes: 0.21 and –0.22 e Å^{-3}. Completeness to Θ = 26.32 (98.67%). Deposition number CCDC 2213942.

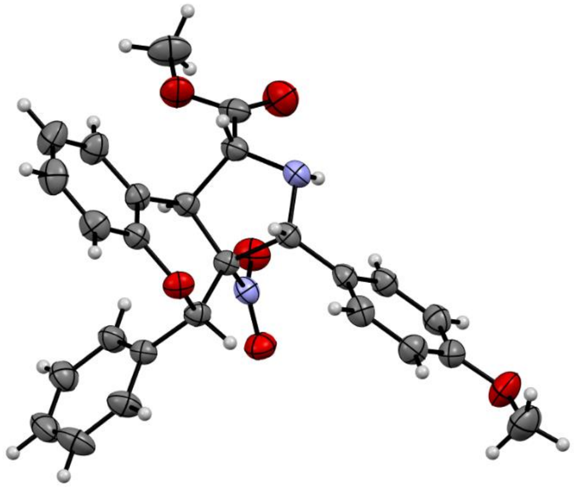

Molecular structure of compound *endo*-**8b** (ORTEP drawing, 50% probability level)

Crystal data for *endo*-9a ($C_{20}H_{17}F_3N_2O_5$, 422.36). Monoclinic crystals, space group $P2_1/n$, a = 16.1467(13), b = 12.4054(7) and c = 19.6639(17) Å, β = 106.101(8)°, V = 3784.3(5) Å^3, D_c = 1.483, absorption coefficient μ = 0.126 mm^{-1}, Z = 8. The intensities of 10373 independent reflections (R_{int} = 0.0544) were measured. The final discrepancy factors R_1 = 0.0524, wR_2 = 0.0978, GooF = 0.982 for

4180 reflections with $I > 2\sigma(I)$; R_1 = 0.1600, wR_2 = 0.1492 (all data). Largest different peaks and holes: 0.17 and –0.20 e Å^{-3}. Completeness to Θ = 25.24 (99.6%). Deposition number CCDC 2213944.

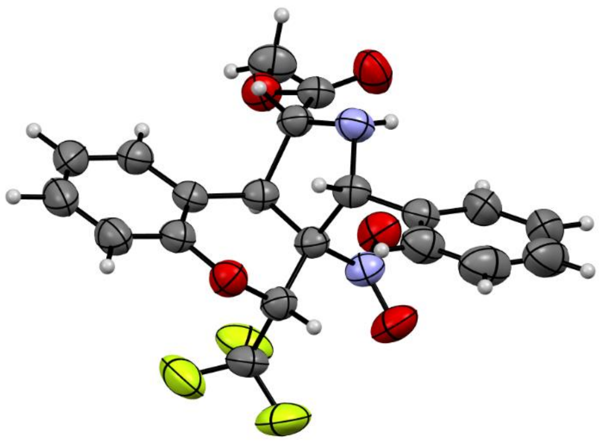

Molecular structure of compound *endo*-**9a** (ORTEP drawing, 50% probability level)

Crystal data for 10c ($C_{21}H_{17}Cl_3N_2O_7$, 515.72). Monoclinic crystals, space group $P2_1/n$, a = 10.7321(16), b = 15.9306(14) and c = 13.532(2) Å, β = 96.095(12)°, V = 2300.5(5) Å^3 , D_c = 1.489, absorption coefficient μ = 0.444 mm^{-1}, Z = 4. The intensities of 5629 independent reflections (R_{int} = 0.0779) were measured. The final discrepancy factors R_1 = 0.0664, wR_2 = 0.1626, GooF = 0.959 for 2034 reflections with $I > 2\sigma(I)$; R_1 = 0.1863, wR_2 = 0.2342 (all data). Largest different peaks and holes: 0.33 and –0.41 e Å^{-3}. Completeness to Θ = 25.24 (98.8%). Deposition number CCDC 2213943.

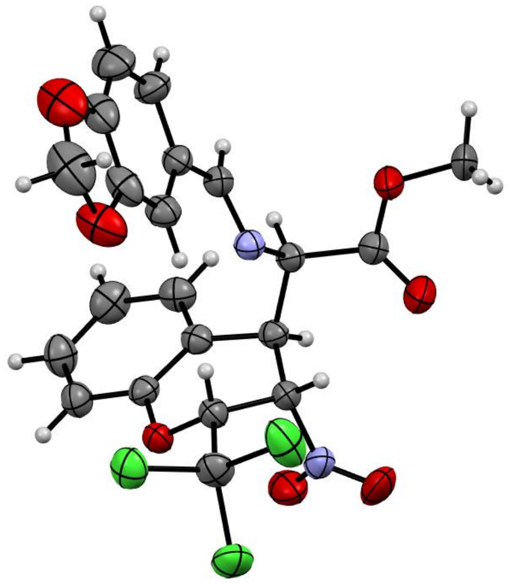

Molecular structure of compound **10c** (ORTEP drawing, 30% probability level)

References

44. Sheldrick, G.M. A short history of SHELX. *Acta Crystallogr.* **2008**, *64*, 112–122.

45. Dolomanov, O. V.; Bourhis, L. J.; Gildea, R. J.; Howard, J. A. K.; Puschmann, H. OLEX2: A complete structure solution, refinement and analysis program. *J. Appl. Crystallogr.* **2009**, *42*, 339–341.
